# Direct access to pyrido/pyrrolo[2,1-*b*]quinazolin-9(1*H*)-ones through silver-mediated intramolecular alkyne hydroamination reactions

**DOI:** 10.3762/bjoc.11.47

**Published:** 2015-03-30

**Authors:** Hengshuai Wang, Shengchao Jiao, Kerong Chen, Xu Zhang, Linxiang Zhao, Dan Liu, Yu Zhou, Hong Liu

**Affiliations:** 1 CAS Key Laboratory of Receptor Research, Shanghai Institute of Materia Medica, Chinese Academy of Sciences, 555 Zuchongzhi Road, Shanghai 201203, P. R. China; 2 Shenyang Pharmaceutical University, 103 Wenhua Road, Shengyang 110016, P. R. China

**Keywords:** heterocyclic molecules, intramolecular alkyne hydroamination, silver

## Abstract

We report a synthetic methodology for the construction of the fused heterocyclic compounds pyrido[2,1-*b*]quinazolin-9(1*H*)-ones and pyrrolo[2,1-*b*]quinazolin-9(1*H*)-ones through an AgOTf-catalyzed intramolecular alkyne hydroamination reaction. The methodology is applicable to a wide scope of substrates and produces a series of fused quinazolinone heterocycles in good to excellent yields.

## Introduction

**Figure 1:**
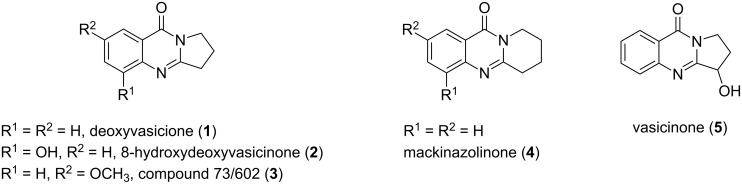
Selected structures of fused quinazolinones.

Quinazolinone is a core skeleton for naturally existing phytochemicals. They were extracted from a variety of plant families. Among the quinazolinone derivatives, such as the pyrrolo[2,1-*b*]quinazolinone alkaloids, are a multitude of biomedically active substances [1–2]. For example, deoxyvasicione (**1**), 8-hydroxydeoxyvasicinone (**2**), compound 73/602 (**3**), mackinazolinone (**4**) and vasicinone (**5**) have been proven to act as bronchodilatory, anti-inflammatory, antimicrobial and antidepressant agents (Figure 1) [2–8].

**Scheme 1:**
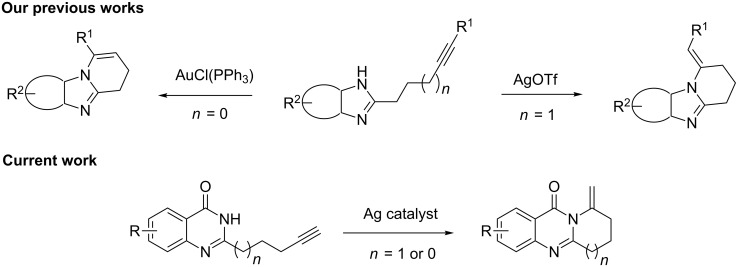
The intramolecular alkyne hydroamination of alkynes.

A variety of approaches have been employed to synthesize deoxyvasicione (**1**) and its derivatives, e.g., the Pd(OAc)_2_-catalyzed carbonyl-insertion reaction [7], the cycloaddition of anthranilic acid iminoketene to a methyl butyrolactam through a sulfinamide anhydride intermediate [9], the intramolecular aza-Wittig reaction with an azide substrate [10], and the cycloaddition of anthranilamide [11]. For the synthesis of vasicinone (**5**), deoxyvasicinone was subjected to a free-radical bromination using NBS and the subsequent treatment with NaOAc/AcOH as an acetoxylation reagent [12]. However, for most of these synthetic strategies harsh reaction conditions are a necessity, produce unstable sulfonamide anhydride intermediates [2,13], which are dangerous substrates bearing an azide group, and require a high reaction temperature and a long reaction time [2,10]. Recently, transition metal catalyzed hydroamination of alkynes [14–26], alkenes [15,27–31] and dienes [32–33] has been widely studied for the construction of heterocycles. We have reported on a highly efficient gold/silver-catalyzed intramolecular hydroamination of terminal alkynes in water for the synthesis of fused tricyclic xanthenes [34]. On the basis of this methodology, we have also afforded two fused benzimidazoles through silver-catalyzed intramolecular hydroamination from readily available starting materials with a long-chain alkyne [35–36]. Motivated by the unique structural properties and the biological activities characteristic of the vasicinone type alkaloids, we extended our work in this direction by elaborating the synthesis of fused quinazolinone derivatives. Herein, we present our recent findings of the synthesis of fused pyrrolo[2,1-*b*]quinazolin-9(1*H*)-ones by a silver-mediated chemoselective and regioselective intramolecular hydroamination cyclization (Scheme 1).

## Results and Discussion

To establish the overall best experimental conditions for the synthesis of pyrido/pyrrolo[2,1-*b*]quinazolin-9(1*H*)-ones, we chose 2-(4-pentynyl)-4(3*H*)-quinazolinone (**6A**) as a model substrate to prepare them by an intramolecular hydroamination cyclization. The results of these experiments are summarized in Table 1. Silver trifluoromethanesulfonate (AgOTf) seemed to be the most effective catalyst for this intramolecular hydroamination cyclization (Table 1, entries 1–6), whereas a product was not afforded in the absence of a catalyst (Table 1, entry 7). We also screened different solvents, and the results demonstrated that non-polar aprotic solvents could promote the reaction. Toluene was the most effective solvent for this cyclization (Table 1, entry 3 and entries 8–16). The concentration of the substrate in the reaction mixture also affected the product yield. When the concentration was changed from 0.1 M to 1 M, the yield dropped to 82% (Table 1, entry 17). Subsequently, we examined the influence of the reaction temperature, and no better yield could be obtained at a temperature either lower or higher than 80 °C (Table 1, entries 18 and 19). A prolongation of the reaction time to 12 h resulted in a slight decrease of the yield (Table 1, entry 20). Performing the reaction without inert gas (argon) atmosphere also led to a decrease of the yield (Table 1, entry 21). In summary, the optimum results were obtained when 2-(4-pentynyl)-4(3*H*)-quinazolinone (**6A**) in toluene was treated with 5 mol % of AgOTf in a sealed tube under argon protection at 80 °C for 3 h (Table 1, entry 3).

**Table 1:** Optimization of the reaction conditions.^a^

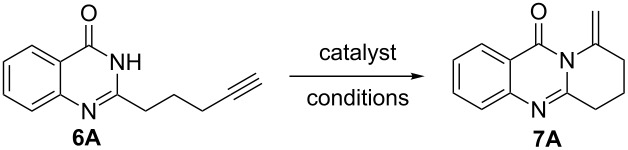			

Entry	Catalyst	Solvent	Yield (%)

1	AgBF_4_	toluene	80
2	AgSbF_6_	toluene	62
3	AgOTf	toluene	90
4	AgNO_3_	toluene	39
5	AgOCOCF_3_	toluene	85
6	AgOAc	toluene	41
7	–	toluene	0
8	AgOTf	1,2-dichloroethane	82
9	AgOTf	1,4-dioxane	70
10	AgOTf	DME	40
11	AgOTf	THF	47
12	AgOTf	DMF	37
13	AgOTf	DMSO	38
14	AgOTf	MeCN	73
15	AgOTf	MeOH	28
16	AgOTf	EtOH	28
17	AgOTf	toluene	82^b^
18	AgOTf	toluene	70^c^
19	AgOTf	toluene	83^d^
20	AgOTf	toluene	85^e^
21	AgOTf	toluene	71^f^

^a^**6A** (0.2 mmol) and catalyst (5 mol %) in the specified solvent (2 mL) were heated in a sealed vial under argon protection at 80 °C for 3 h; ^b^the concentration of **6A** is 1 M; ^c^the reaction temperature was 60 °C; ^d^the reaction temperature was 100 °C; ^e^the reaction time was 12 h; ^f^the reaction was performed without an argon inert gas atmosphere.

To evaluate the scope of the proposed silver-catalyzed intramolecular hydroamination cyclization reaction, we investigated its tolerance by probing changes in the substituted 2-(4-pentynyl)-4(3*H*)-quinazolinone (**6A**) under the optimum reaction conditions mentioned above (Table 2, entries 1–12). Various substituted 2-(4-pentynyl)-4(3*H*)-quinazolinones (**6A–L**) were tolerant of this transformation, and the desired products **7A–L** were afforded with moderate to excellent yields (53–91%). It seems that the position and type of substituents on the 2-(4-pentynyl)-4(3*H*)-quinazolinones (**6A**) only slightly affected the yields of the target compounds (Table 2, entries 1–9). Higher yields could be obtained when the 6- and 7-positions of the 2-(4-pentynyl)-4(3*H*)-quinazolinone were substituted by methyl and methoxy groups (Table 2, entries 2–4). The introduction of a fluorine, a chlorine and a bromine atom at 5-, 6- and 7-positions resulted in a slight reduction of the yield of the products (Table 2, entries 5–9). However, a bulky phenyl group introduced at the 7-position led to a good yield (Table 2, entry 10). When the benzene ring of the skeleton of the substrate was replaced by a naphthalene ring, the product was obtained at a comparable yield of 87% (Table 2, entry 11). However, with 2-(4-pentynyl)-thieno[2,3-*d*]pyrimidin-4(1*H*)-one (**6L**) as a substrate, the reaction was significantly different compared to the other substituted 2-(4-pentynyl)-4(3*H*)-quinazolinones (Table 2, entry 12). Although the thieno analogue **6L** was tolerated in the reaction, the cyclization required substantially longer (12 h), and the product was obtained in a relatively low yield (only 53%).

**Table 2:** Silver-mediated synthesis of target compounds **7A–L**.^a^

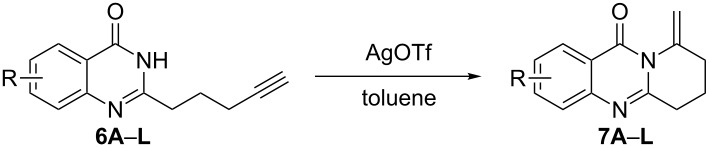			

Entry	Substrate	Product	Yield (%)

1	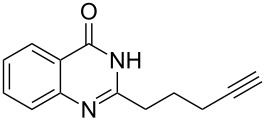	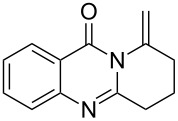 **7A**	90
2	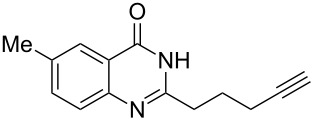	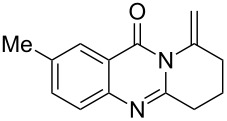 **7B**	91
3	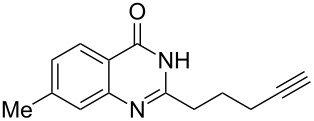	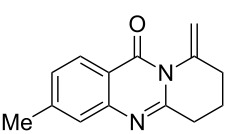 **7C**	89
4	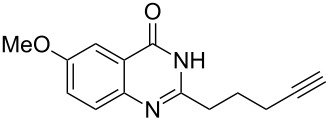	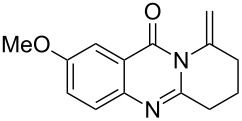 **7D**	93
5	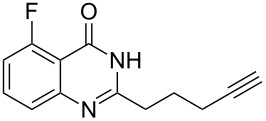	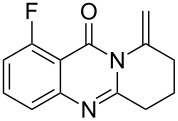 **7E**	82
6	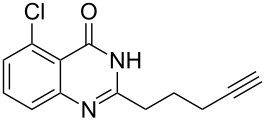	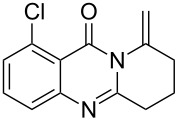 **7F**	83
7	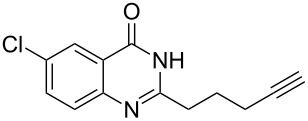	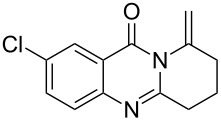 **7G**	84
8	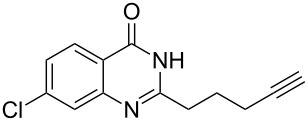	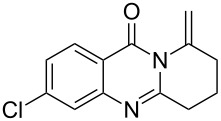 **7H**	85
9	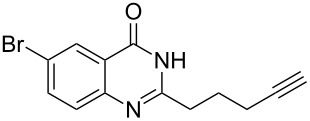	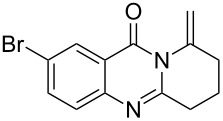 **7I**	86
10	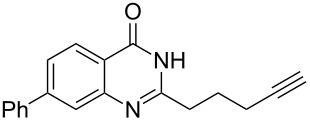	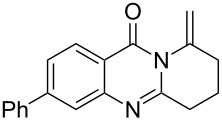 **7J**	89
11	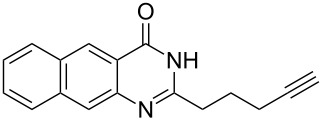	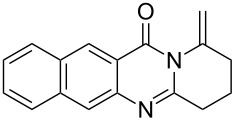 **7K**	87
12	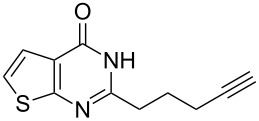	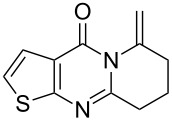 **7L**	53^b^

^a^Substrates **6A–L** (0.4 mmol) and catalyst (5 mol %) in anhydrous toluene (4 mL) were heated in a sealed vial under argon atmosphere at 80 °C for 3 h; ^b^the reaction time was 12 h.

Further studies indicated that 2,3-dihydropyrrolo[2,1-*b*]quinazolin-9(1*H*)-ones **9A–L** could be generated by the treatment of substituted 2-(3-butynyl)-4(3*H*)-quinazolinones **8A–L** with AgOTf under the optimized reaction conditions. As illustrated in Table 3, 2-(3-butynyl)-4(3*H*)-quinazolinones **8A–L** with different substituents were well-tolerated in this intramolecular cyclization reaction, and the expected products **9A–L** were obtained in good to excellent yields (80–93%, Table 3, entries 1–12).

**Table 3:** Silver-mediated synthesis of target compounds **9A–L**.^a^

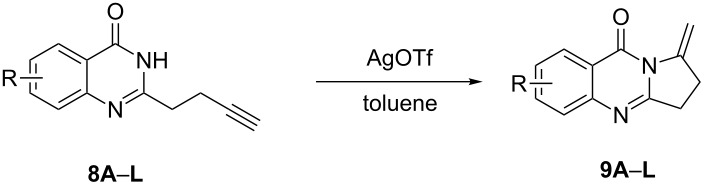			

Entry	Substrate	Product	Yield (%)

1	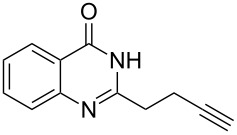	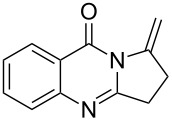 **9A**	93
2	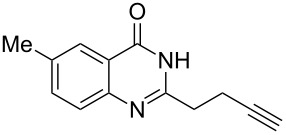	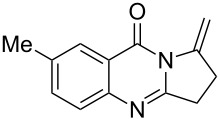 **9B**	89
3	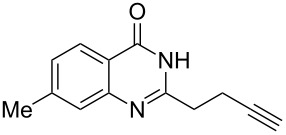	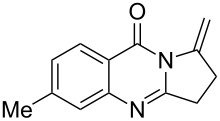 **9C**	90
4	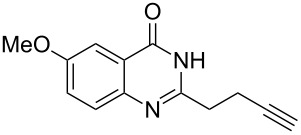	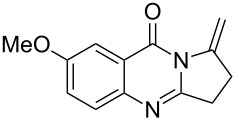 **9D**	92
5	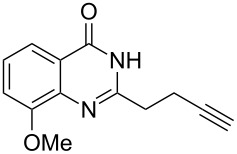	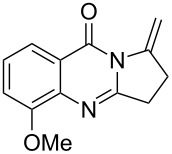 **9E**	90
6	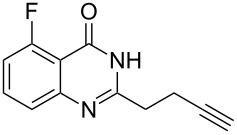	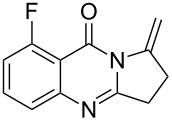 **9F**	80
7	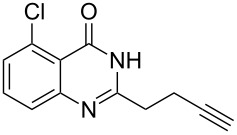	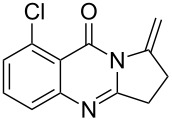 **9G**	82
8	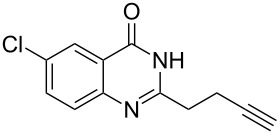	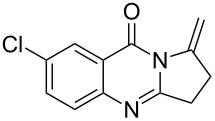 **9H**	84
9	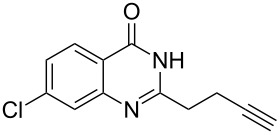	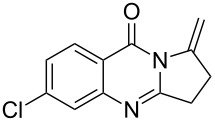 **9I**	83
10	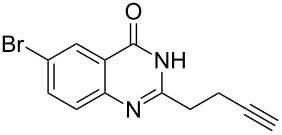	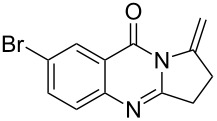 **9J**	83
11	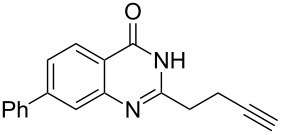	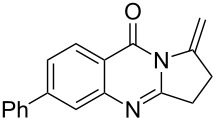 **9K**	91
12	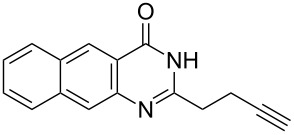	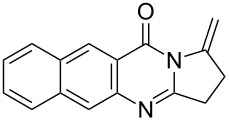 **9L**	86
13	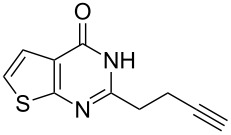	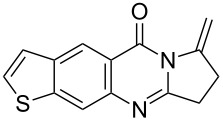 **9M**	—^b^

^a^Substrates **8A–L** (0.4 mmol) and catalyst (5 mol %) in anhydrous toluene (4 mL) were heated in a sealed vial under argon atmosphere at 80 °C for 3 h; ^b^the product could not be isolated due to large amounts of impurities formed during the reaction.

**Scheme 2:**
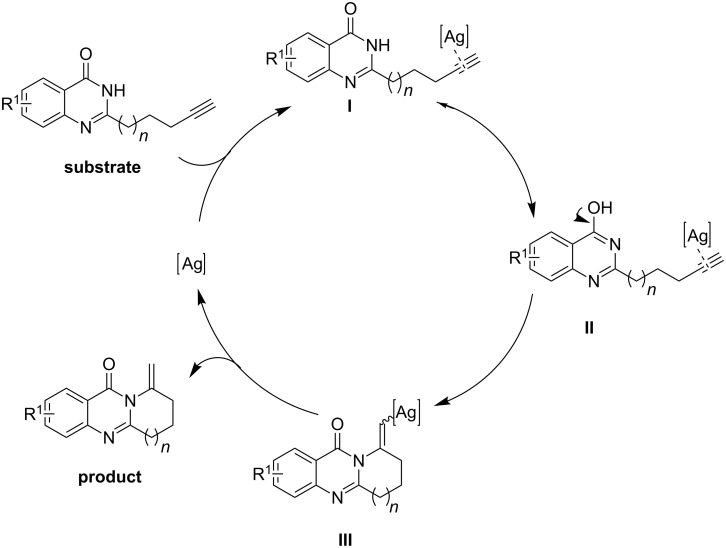
A plausible mechanism.

Based on the results of the present studies, we propose a plausible mechanism for the transformation. As depicted in Scheme 2, the intramolecular cyclization is initiated by the activation of the terminal alkyne moiety of the substrate with AgOTf to generate the Ag–alkyne π complex **I** (or its tautomer **II**). Subsequently, the Ag–alkyne π complex **I** or **II** is converted into complex **III** through a nucleophilic attack of the nitrogen atom of the amide, and then produces the final product. Products **7A** and **9G** were recrystallized and their structures were unambiguously confirmed by X-ray diffraction (XRD) studies (see Supporting Information File 1 for details).

## Conclusion

In conclusion, we have developed a chemical methodology for the synthesis of pyrido/pyrrolo[2,1-*b*]quinazolin-9(1*H*)-ones through an AgOTf-catalyzed intramolecular alkyne hydroamination cyclization reaction. The methodology is applicable to a wide scope of substrates and generates a series of fused quinazolinone heterocycles in good to excellent yields. It lends itself an alternative method to the construction of innovative molecules with polycyclic architectures. It may be worthwhile to investigate the biological activity of the synthesized structures.

## Experimental

Commercially available reagents and solvents were used without further purification. Column chromatography was performed on silica gel. TLC was performed on silica gel GF254 plates. ^1^H NMR and ^13^C NMR spectra were obtained on Varian 300, Bruker 400 and 500 spectrometers. The chemical shifts for ^1^H NMR were recorded in parts per million (ppm) downfield from tetramethylsilane (TMS) with the residual solvent resonance as the internal standard (7.26 ppm for CDCl_3_ or 2.50 ppm for DMSO-*d*_6_). The chemical shifts for ^13^C NMR were recorded in ppm by using the central peak of CDCl_3_ (77.23 ppm) or DMSO-*d*_6_ (39.52 ppm) as the internal standard. Coupling constants (*J*) are reported in Hz and refer to apparent peak multiplications. The abbreviations *s*, *d*, *t*, *q*, *p* and *m* stand for singlet, doublet, triplet, quartet, pentet and multiplet, respectively.

**General procedure for the synthesis of substrates 6A–6L and 8A–8L:** To a solution of 5-hexynoic acid (3.0 mmol) in dry CH_2_Cl_2_ (5 mL) was added EDCI (3.1 mmol) and HOBt (3.1 mmol). The resulting mixture was stirred at rt for 2 h. Then substituted or unsubstituted 2-aminobenzamide (3.0 mmol) was added, and the reaction mixture was stirred at rt for 12 h while being monitored by TLC. After the addition of H_2_O (10 mL) the mixture was extracted with ethyl acetate (3 × 20 mL). The organic layers were combined and concentrated under vacuum to give the amide intermediate.

The above intermediate was then dissolved in 95% EtOH (5 mL), and solid NaOH (6.0 mmol) was added. The mixture was heated under reflux for 2 h while being monitored by TLC. The solvent was evaporated under vacuum. Water (10 mL) was added, and the mixture was extracted with ethyl acetate (3 × 20 mL). The organic layers were combined and dried over anhydrous Na_2_SO_4_. After the removal of the solvent the crude product was purified by silica gel column chromatography with CH_2_Cl_2_/MeOH 50:1 (v/v) as an eluent to give the desired substrates **6A–6L**.

For **8A–8L**, the same procedure as described above was used, except that 4-pentynoic acid was used instead of 5-hexynoic acid. Compound **6A** as an example: ^1^H NMR (300 MHz, CDCl_3_) δ 11.75 (s, 1H), 8.29 (dd, *J* = 8.0, 1.0 Hz, 1H), 7.82–7.73 (m, 1H), 7.73–7.66 (m, 1H), 7.52–7.43 (m, 1H), 2.98–2.88 (m, 2H), 2.41 (td, *J* = 6.9, 2.6 Hz, 2H), 2.21–2.08 (m, 2H), 2.01 (t, *J* = 2.6 Hz, 1H); ^13^C NMR (100 MHz, CDCl_3_) δ 164.3, 155.8, 149.4, 134.8, 127.3, 126.5, 128.2, 120.5, 83.2, 69.4, 34.4, 25.8, 18.0. LRMS (ESI) *m*/*z*: 213 [M + H]^+^; HRMS–ESI (*m*/*z*): [M + H]^+^ calcd for C_13_H_13_N_2_O, 213.1028; found, 213.1024.

**General procedure for the synthesis of the target products 7A–7L and 9A–9L**: A vial equipped with a magnetic stir bar was charged with the corresponding substrate **6A–6L** or **8A–8L** (0.4 mmol) and the catalyst AgOTf (5 mol %) and capped with a septum. The vial was evacuated and backfilled with argon, and this process was repeated three times. Under argon, anhydrous toluene (4 mL) was injected to the vial with a syringe, and the resulting mixture was stirred at rt for 10 min. Afterwards, the vial was kept in a preheated oil bath at 80 °C for the appropriate time. After the reaction was complete, the reaction mixture was cooled to rt and the solvent was evaporated under vacuum. The residue was purified by silica gel column chromatography with petroleum ether/EtOAc 20:1 (v/v) as an eluent to give the desired target compounds **7A–7L** and **9A–9L**. Compound **7A** as an example: ^1^H NMR (400 MHz, CDCl_3_) δ 8.30 (dd, *J* = 8.0, 1.5 Hz, 1H), 7.82–7.66 (m, 1H), 7.61 (d, *J* = 8.1 Hz, 1H), 7.50–7.37 (m, 1H), 5.58 (s, 1H), 5.45 (s, 1H), 2.84 (t, *J* = 6.9 Hz, 2H), 2.76–2.56 (m, 2H), 2.00 (dt, *J* = 14.4, 7.1 Hz, 2H); ^13^C NMR (100 MHz, CDCl_3_) δ 160.3, 155.8, 146.9, 136.9, 134.4, 127.4, 126.6, 126.5, 121.3, 112.5, 31.8, 29.6, 18.3; LRMS (EI) *m/z*: 212 [M]^+^; HRMS–EI (*m*/*z*): [M]^+^ calcd for C_13_H_12_N_2_O, 212.0950; found, 212.0930.

## Supporting Information

File 1 Detailed experimental procedures for all compounds and precursors, copies of ^1^H/^13^C NMR spectra for all compounds.
