# Supplementary material for: Direct access to pyrido/pyrrolo[2,1-b]quinazolin-9(1H)-ones through silver-mediated intramolecular alkyne hydroamination reactions
Source: Beilstein J Org Chem. 2015 Mar 30;11:416–24. doi: 10.3762/bjoc.11.47 (PMC4419559; doi:10.3762/bjoc.11.47)

Supporting Information
for
Direct access to pyrido/pyrrolo[2,1-*b*]quinazolin-9(1*H*)-ones through silver-mediated intramolecular alkyne hydroamination reactions

Hengshuai Wang[1], Shengchao Jiao[1,2], Kerong Chen[1], Xu Zhang[1], Linxiang Zhao[2], Dan Liu[2], Yu Zhou* [1] and Hong Liu[1]

Address: [1]CAS Key Laboratory of Receptor Research, Shanghai Institute of Materia Medica, Chinese Academy of Sciences, 555 Zuchongzhi Road, Shanghai 201203, P. R. China and [2]Shenyang Pharmaceutical University, 103 Wenhua Road, Shengyang 110016, P. R. China

Email: Yu Zhou - zhouyu@simm.ac.cn

* Corresponding author

Detailed experimental procedures for all compounds and precursors, copies of $^{1}H/^{13}C$ NMR spectra for all compounds

Contents

X-ray crystallographic structure of **7A** and **9G** S3

Detailed experimental procedures for all compounds S9

Copies of ^{1}H NMR and ^{13}C NMR of compounds S31

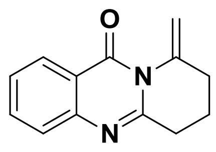

7A

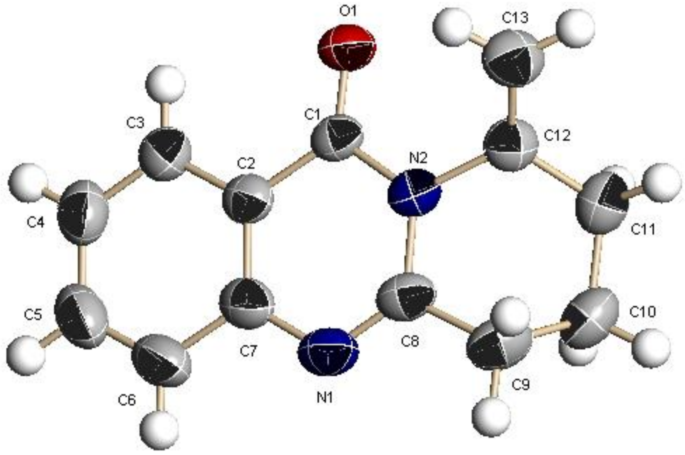

Figure S1: X-ray crystallographic structure of **7A**.

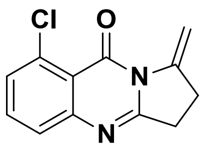

9G

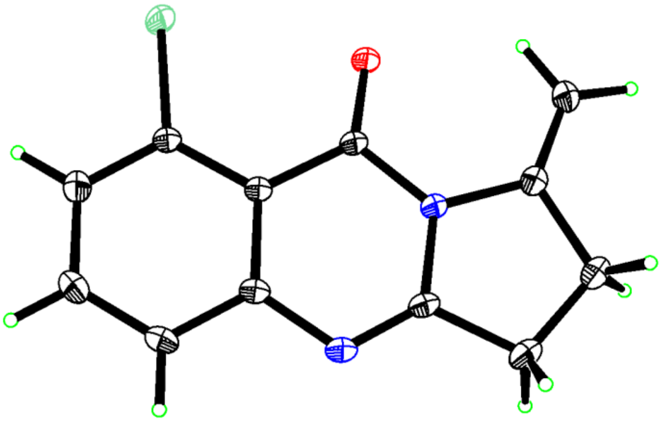

Figure S2: X-ray crystallographic structure of **9G**.

The Details of X-ray crystallographic structure of 7A

Bond precision:	C-C = 0.0021 A		Wavelength=0.71073
Cell:	a=11.846(5)	b=9.163(4)	c=10.019(4)
	alpha=90	beta=103.216(8)	gamma=90
Temperature:	293 K		

	Calculated	Reported
Volume	1058.7(8)	1058.7(8)
Space group	P21/c	P21/c
Hall group	-P 2ybc	?
Moiety formula	C13 H12 N2 O	?
Sum formula	C13 H12 N2 O	C13 H12 N2 O
Mr	212.25	212.25
Dx,g cm-3	1.332	1.332
Z	4	4
Mu (mm-1)	0.086	0.086
F000	448.0	448.0
F000'	448.17	
h,k,lmax	14,11,12	14,11,12
Nref	2082	2078
Tmin,Tmax	0.978,0.988	0.713,1.000
Tmin'	0.977	

Correction method= EMPIRICAL

Data completeness= 0.998 Theta(max)= 26.000

R(reflections)= 0.0396(1656) wR2(reflections)= 0.1167(2078)

S = 1.024 Npar= 145

CCDC 986414 contains the supplementary crystallographic data for this paper. These data can be also obtained free of charge from The Cambridge Crystallographic Data Centre *via* www.ccdc.cam.ac.uk/data_request/cif.

The Details of X-ray crystallographic structure of 9G

Bond precision: C-C = 0.0037 A Wavelength=0.71073

Cell:	a=7.6459(6)	b=6.7006(6)	c=10.4656(7)
	alpha=90	beta=106.548(5)	gamma=90

Temperature: 296 K

	Calculated	Reported
Volume	513.97(7)	513.97(7)
Space group	P21/m	P21/m
Hall group	-P 2yb	?
Moiety formula	C12 H9 Cl N2 O	?
Sum formula	C12 H9 Cl N2 O	C12 H9 Cl N2 O
Mr	232.66	232.66
Dx,g cm-3	1.503	1.503

Z	2	2
Mu (mm-1)	0.348	0.348
F000	240.0	290.0
F000'	240.38	
h,k,lmax	9,8,13	9,8,13
Nref	1288	1279
Tmin,Tmax	0.967,0.973	0.870,0.952
Tmin'	0.923	

Correction method= EMPIRICAL

Data completeness= 0.993 Theta(max)= 27.600

R(reflections)= 0.0472(912) wR2(reflections)= 0.1056(1279)

S = 1.310 Npar= 97

CCDC 986413 contains the supplementary crystallographic data for this paper. These data can be also obtained free of charge from The Cambridge Crystallographic Data Centre *via* www.ccdc.cam.ac.uk/data_request/cif.

Detailed experimental procedures for all compounds

Commercially available reagents and solvents were used without further purification. Column chromatography was performed on silica gel. TLC was performed on silica gel GF254 plates. ^{1}H NMR and ^{13}C NMR spectra were obtained on Varian 300, Bruker 400 and 500 spectrometers. The chemical shifts for ^{1}H NMR were recorded in parts per million (ppm) downfield from tetramethylsilane (TMS) with the residual solvent resonance as the internal standard (7.26 ppm for $CDCl_3$ or 2.50 ppm for DMSO-d_6). The chemical shifts for ^{13}C NMR were recorded in ppm using the central peak of $CDCl_3$ (77.23 ppm) or DMSO-d_6 (39.52 ppm) as the internal standard. Coupling constants (J) are reported in Hz and refer to apparent peak multiplications. The abbreviations *s*, *d*, *t*, *q*, *p* and *m* stand for singlet, doublet, triplet, quartet, pentet and multiplet in that order.

General Procedure for Synthesis of the Substrates (6A–6L) and (8A–8L): To a solution of 5-hexynoic acid (3.0 mmol) in dry CH_2Cl_2 (5 mL) was added EDCI (3.1 mmol) and HOBt (3.1 mmol). The resulting mixture was stirred at room temperature for 2 hours, and then substituted or unsubstituted 2-aminobenzamide (3.0 mmol) was added. The reaction mixture was stirred at room temperature for 12 h with being monitored by TLC. Water (10 mL) was added to the mixture and extracted with ethyl acetate (3 × 20 mL). The organic layers were combined and concentrated under vacuum to give the amide intermediate.

The intermediate above was dissolved in 95% EtOH (5 mL) and solid NaOH (6.0 mmol) was added. The mixture was refluxed for 2 hours with being monitored by TLC. The solvent was evaporated under vacuum. Water (10 mL) was added and the mixture was extracted with

ethyl acetate (3 × 20 mL). The organic layers were combined and dried over anhydrous Na_2SO_4. After removal of the solvent, the crude product was purified by silica gel column chromatography (CH_2Cl_2/MeOH = 50/1, v/v, as an eluent) to give the desired substrates **6A–6L**.

For **8A–8L**, the same procedure as described above was used with 4-pentynoic acid instead of 5-hexynoic acid.

General procedure for the synthesis of the target products 7A–7L and 9A–9L: A vial equipped with a magnetic stir bar was charged with a substrate **6A–6L** and **8A–8L** (0.4 mmol) and the catalyst silver trifluoromethanesulfonate (AgOTf, 5 mol %) and capped with a septum. The vial was evacuated and backfilled with argon and the process was repeated three times. Under argon, anhydrous toluene (4 mL) was injected to the vial with a syringe, and then the resulting mixture was stirred at room temperature for 10 min. Afterwards, the vial was kept in a preheated oil bath at 80 °C for the appropriate time. After the reaction was complete, the reaction mixture was cooled to room temperature and the solvent was evaporated under vacuum. The residue was purified by silica gel column chromatography (petroleum ether/EtOAc = 20/1, v/v, as an eluent) to give the desired target compounds **7A–7L** and **9A–9L**.

2-(4-Pentynyl)-4(3*H*)-quinazolinone (6A):

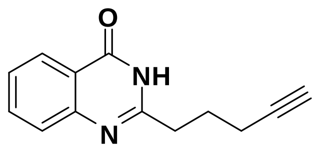

^{1}H NMR (300 MHz, $CDCl_3$) δ 11.75 (s, 1H), 8.29 (dd, *J* = 8.0, 1.0 Hz, 1H), 7.82 – 7.73 (m, 1H), 7.73 – 7.66 (m, 1H), 7.52 – 7.43 (m, 1H), 2.98 – 2.88 (m, 2H), 2.41 (td, *J* = 6.9, 2.6 Hz, 2H), 2.21 – 2.08 (m, 2H), 2.01 (t, *J* = 2.6 Hz, 1H). ^{13}C NMR (100 MHz, $CDCl_3$) δ 164.3, 155.8, 149.4, 134.8, 127.3, 126.5, 128.2, 120.5, 83.2, 69.4, 34.4, 25.8, 18.0. LRMS (ESI) *m/z* 213 $[M+H]^+$; HRMS (ESI) calcd for $C_{13}H_{13}N_2O$ $[M+H]^+$ 213.1028, found 213.1024.

2-(4-Pentynyl)-6-methyl-4(3*H*)-quinazolinone (6B):

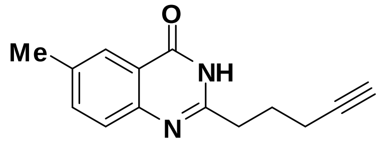

^{1}H NMR (400 MHz, DMSO-d_6) δ 12.10 (s, 1H), 7.86 (br s, 1H), 7.58 (dd, *J* = 8.3, 1.3 Hz, 1H), 7.50 (d, *J* = 8.3 Hz, 1H), 2.81 (t, *J* = 2.6 Hz, 1H), 2.68 (t, *J* = 7.6 Hz, 2H), 2.42 (s, 3H), 2.26 (td, *J* = 7.1, 2.6 Hz, 2H), 1.95 – 1.87 (m, 2H). ^{13}C NMR (100 MHz, DMSO-d_6) δ 161.7, 155.7, 146.8, 135.5, 126.7, 125.1, 120.6, 83.9, 71.8, 33.2, 25.3, 20.8, 17.3. LRMS (ESI) *m/z* 227 $[M+H]^+$; HRMS (ESI) calcd for $C_{14}H_{15}N_2O$ $[M+H]^+$ 227.1184, found 227.1189.

2-(4-Pentynyl)-7-methyl-4(3*H*)-quinazolinone (6C):

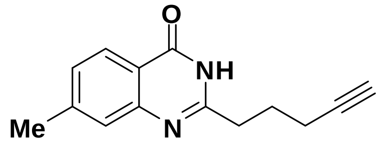

^{1}H NMR (300 MHz, DMSO-d_6) δ 12.08 (s, 1H), 7.95 (d, *J* = 8.1 Hz, 1H), 7.41 – 7.39 (m, 1H), 7.27 (dd, *J* = 8.1, 1.1 Hz, 1H), 2.81 (t, *J* = 2.6 Hz, 1H), 2.68 (t, *J* = 7.5 Hz, 2H), 2.43 (s, 3H), 2.26 (td, *J* = 7.1, 2.6 Hz, 2H), 2.01 – 1.81 (m, 2H). ^{13}C NMR (125 MHz, DMSO-d_6) δ 161.7, 156.7,

149.0, 144.7, 127.4, 126.6, 125.6, 118.5, 83.9, 71.8, 33.2, 25.3, 21.4, 17.3. LRMS (ESI) *m/z* 227 $[M+H]^+$; HRMS (ESI) calcd for $C_{14}H_{15}N_2O$ $[M+H]^+$ 227.1184, found 227.1186.

2-(4-Pentynyl)-6-methoxy-4(3*H*)-quinazolinone (6D):

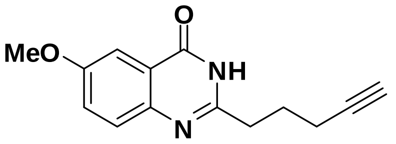

^{1}H NMR (400 MHz, DMSO-d_6) δ 12.12 (s, 1H), 7.54 (d, *J* = 8.9 Hz, 1H), 7.46 (d, *J* = 2.9 Hz, 1H), 7.36 (dd, *J* = 8.9, 3.0 Hz, 1H), 3.85 (s, 3H), 2.81 – 2.77 (m, 1H), 2.67 (t, *J* = 7.6 Hz, 2H), 2.26 (td, *J* = 7.0, 2.5 Hz, 2H), 1.90 (p, *J* = 7.2 Hz, 2H). ^{13}C NMR (125 MHz, DMSO-d_6) δ 161.6, 157.3, 154.3, 143.3, 128.5, 123.7, 121.6, 105.7, 84.0, 71.8, 55.6, 33.1, 25.4, 17.3. LRMS (ESI) *m/z* 243 $[M+H]^+$; HRMS (ESI) calcd for $C_{14}H_{15}N_2O_2$ $[M+H]^+$ 243.1134, found 243.1128.

2-(4-Pentynyl)-5-fluoro-4(3*H*)-quinazolinone (6E):

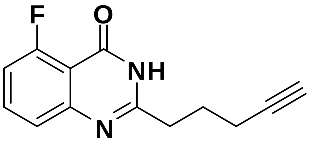

^{1}H NMR (400 MHz, DMSO-d_6) δ 12.22 (s, 1H), 7.73 (td, *J* = 8.2, 5.7 Hz, 1H), 7.40 (d, *J* = 8.2 Hz, 1H), 7.18 (ddd, *J* = 10.9, 8.2, 0.8 Hz, 1H), 2.81 (t, *J* = 2.5 Hz, 1H), 2.67 (t, *J* = 7.6 Hz, 2H), 2.26 (td, *J* = 7.1, 2.6 Hz, 2H), 1.90 (p, *J* = 7.2 Hz, 2H). ^{13}C NMR (125 MHz, DMSO-d_6) δ 160.5 (d, *J* = 262.3 Hz), 159.1 (d, *J* = 2.7 Hz), 157.9, 151.1, 134.8 (d, *J* = 10.6 Hz), 122.9 (d, *J* = 3.4 Hz), 112.4 (d, *J* = 20.4 Hz), 110.4 (d, *J* = 6.1 Hz), 83.9, 71.8, 33.1, 25.2, 17.3. LRMS (ESI) *m/z* 231 $[M+H]^+$; HRMS (ESI) calcd for $C_{13}H_{11}N_2OFNa$ $[M+Na]^+$ 253.0753, found 253.0757.

2-(4-Pentynyl)-5-chloro-4(3*H*)-quinazolinone (6F):

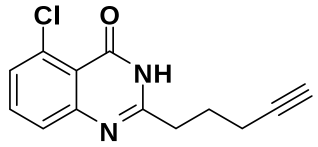

^{1}H NMR (400 MHz, DMSO-d_6) δ 12.23 (s, 1H), 7.70 – 7.62 (m, 1H), 7.55 – 7.49 (m, 1H), 7.46 – 7.41 (m, 1H), 2.82 – 2.81 (m, 1H), 2.66 (t, *J* = 7.6 Hz, 2H), 2.26 (td, *J* = 7.1, 2.6 Hz, 2H), 1.90 (p, *J* = 7.2 Hz, 2H). ^{13}C NMR (100 MHz, DMSO-d_6) δ 159.9, 157.6, 151.4, 134.0, 132.3, 128.4, 126.5, 117.9, 83.9, 71.8, 33.0, 25.1, 17.3. LRMS (EI) *m/z* 246 [M]$^+$; HRMS (EI) calcd for $C_{13}H_{11}ClN_2O$ [M]$^+$ 246.0560, found 246.0563.

2-(4-Pentynyl)-6-chloro-4(3*H*)-quinazolinone (6G):

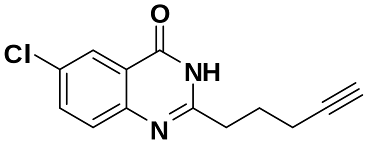

^{1}H NMR (500 MHz, DMSO-d_6) δ 12.38 (s, 1H), 7.99 (br s, 1H), 7.78 (dd, *J* = 8.7, 2.5 Hz, 1H), 7.62 (d, *J* = 8.7 Hz, 1H), 2.83 – 2.80 (m, 1H), 2.69 (t, *J* = 7.6 Hz, 2H), 2.26 (td, *J* = 7.0, 2.6 Hz, 2H), 1.91 (p, *J* = 7.2 Hz, 2H). ^{13}C NMR (125 MHz, DMSO-d_6) δ 160.8, 157.4, 147.6, 134.4, 130.2, 129.2, 124.7, 122.19, 83.9, 71.8, 33.3, 25.2, 17.3. LRMS (ESI) *m/z* 247 [M+H]$^+$; HRMS (ESI) calcd for $C_{13}H_{12}N_2OCl$ [M+H]$^+$ 247.0638, found 247.0641.

2-(4-Pentynyl)-7-chloro-4(3*H*)-quinazolinone (6H):

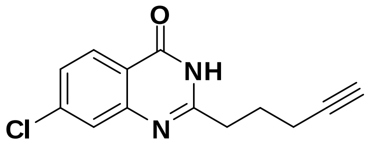

^{1}H NMR (500 MHz, DMSO-d_6) δ 12.34 (s, 1H), 8.06 (d, *J* = 8.0 Hz, 1H), 7.65 (s, 1H), 7.48 (d, *J* = 7.8 Hz, 1H), 2.82 (br s, 1H), 2.76 – 2.62 (m, 2H), 2.31 – 2.22 (m, 2H), 1.99 – 1.81 (m, 2H). ^{13}C NMR (125 MHz, DMSO-d_6) δ 161.2, 158. 5, 150.0, 138.9, 127.8, 126.3, 126.0, 119.8, 83.9,

71.8, 33.3, 25.3, 17.3. LRMS (ESI) *m/z* 247 $[M+H]^+$; HRMS (ESI) calcd for $C_{13}H_{12}N_2OCl$ $[M+H]^+$ 247.0638, found 247.0641.

2-(4-Pentynyl)-6-bromo-4(3*H*)-quinazolinone (6I):

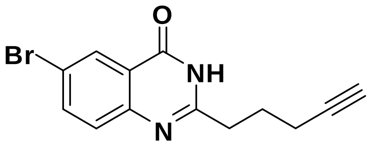

^1H NMR (500 MHz, DMSO-d_6) δ 12.38 (s, 1H), 8.19 – 8.08 (m, 1H), 7.89 (dd, *J* = 8.5, 1.5 Hz, 1H), 7.54 (d, *J* = 8.6 Hz, 1H), 2.81 (d, *J* = 2.2 Hz, 1H), 2.69 (t, *J* = 7.6 Hz, 2H), 2.26 (td, *J* = 7.0, 2.6 Hz, 2H), 1.91 (p, *J* = 7.2 Hz, 2H). ^{13}C NMR (125 MHz, DMSO-d_6) δ 160.7, 157.5, 147.8, 137.1, 129.3, 127.8, 122. 6, 118.3, 83.9, 71.8, 33.3, 25.2, 17.3. LRMS (EI) *m/z* 290 $[M]^+$; HRMS (EI) calcd for $C_{13}H_{11}BrN_2O$ $[M]^+$ 290.0055, found 290.0016.

2-(4-Pentynyl)-7-phenyl-4(3*H*)-quinazolinone (6J):

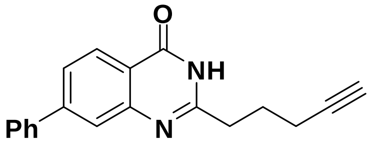

^1H NMR (400 MHz, DMSO-d_6) δ 12.23 (s, 1H), 8.14 (d, *J* = 8.3 Hz, 1H), 7.85 (d, *J* = 1.6 Hz, 1H), 7.83 – 7.78 (m, 2H), 7.77 (dd, *J* = 8.3, 1.7 Hz, 1H), 7.54 – 7.48 (m, 2H), 7.48 – 7.41 (m, 1H), 2.84 (t, *J* = 2.6 Hz, 1H), 2.72 (t, *J* = 7.6 Hz, 2H), 2.29 (td, *J* = 7.0, 2.6 Hz, 2H), 1.94 (p, *J* = 7.1 Hz, 2H). ^{13}C NMR (100 MHz, DMSO-d_6) δ 161.6, 157.2, 149.4, 145.9, 138.9, 129.2, 128.6, 127.2, 126.5, 124.7, 124.4, 119.8, 84.0, 71.9, 33.4, 25.4, 17.4. LRMS (ESI) *m/z* 289 $[M+H]^+$; HRMS (ESI) calcd for $C_{19}H_{17}N_2O$ $[M+H]^+$ 289.1341, found 289.1346.

2-(4-Pentynyl)-4(3*H*)-benzo[*g*]quinazolinone (6K):

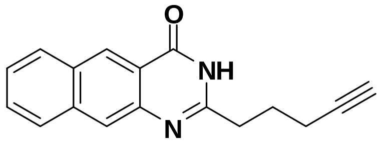

^{1}H NMR (400 MHz, DMSO-d_6) δ 12.02 (s, 1H), 8.78 (s, 1H), 8.15 – 8.19 (m, 2H), 8.06 (d, *J* = 8.4 Hz, 1H), 7.69 – 7.60 (m, 1H), 7.59 – 7.51 (m, 1H), 2.83 (t, *J* = 2.5 Hz, 1H), 2.73 (t, *J* = 7.5 Hz, 2H), 2.31 (td, *J* = 7.0, 2.6 Hz, 2H), 1.97 (p, *J* = 7.2 Hz, 2H). ^{13}C NMR (125 MHz, DMSO-d_6) δ 162.2, 155.7, 144.3, 136.2, 130.6, 129.2, 128.4, 127.8, 127.1, 126.0, 124.1, 120.2, 84.0, 71.8, 33.4, 25.1, 17.4. LRMS (ESI) *m/z* 263 $[M+H]^+$; HRMS (ESI) calcd for $C_{17}H_{15}N_2O$ $[M+H]^+$ 263.1184, found 263.1190.

2-(4-Pentynyl)-thieno[2,3-*d*]pyrimidin-4(1*H*)-one (6L):

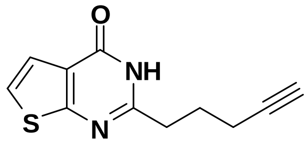

^{1}H NMR (400 MHz, DMSO-d_6) δ 12.39 (s, 1H), 7.48 (d, *J* = 5.7 Hz, 1H), 7.33 (d, *J* = 5.7 Hz, 1H), 2.81 (br s, 1H), 2.72 (t, *J* = 7.5 Hz, 2H), 2.25 (td, *J* = 7.2, 2.6 Hz, 1H), 1.89 (p, *J* = 7.1 Hz, 2H). ^{13}C NMR (100 MHz, DMSO-d_6) δ 164.8, 158.4, 157.6, 122.8, 122.4, 121.5, 83.8, 71.9, 32.8, 25.4, 17.3. LRMS (EI) *m/z* 218 $[M]^+$; HRMS (EI) calcd for $C_{11}H_{10}N_2OS$ $[M+H]^+$ 218.0514, found 218.0510.

6,7,8,9-Tetrahydro-9-methylene-11*H*-pyrido[2,1-*b*]quinazolin-11-one (7A):

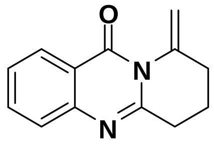

76 mg, 90% yield, white solid, m. p. 78.1-81.5 °C. ^{1}H NMR (400 MHz, $CDCl_3$) δ 8.30 (dd, *J* = 8.0, 1.5 Hz, 1H), 7.82 – 7.66 (m, 1H), 7.61 (d, *J* = 8.1 Hz, 1H), 7.50 – 7.37 (m, 1H), 5.58 (s, 1H), 5.45

(s, 1H), 2.84 (t, *J* = 6.9 Hz, 2H), 2.76 – 2.56 (m, 2H), 2.00 (dt, *J* = 14.4, 7.1 Hz, 2H). ^{13}C NMR (100 MHz, $CDCl_3$) δ 160.3, 155.8, 146.9, 136.9, 134.4, 127.4, 126.6, 126.5, 121.3, 112.5, 31.8, 29.6, 18.3. LRMS (EI) *m/z* 212 $[M]^+$. HRMS (EI) calcd for $C_{13}H_{12}N_2O$ $[M]^+$ 212.0950, found 212.0930.

6,7,8,9-Tetrahydro-2-methyl-9-methylene-11*H*-pyrido[2,1-*b*]quinazolin-11-one (7B):

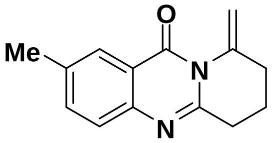

82 mg, 91% yield. ^{1}H NMR (400 MHz, $CDCl_3$) δ 8.10 – 8.06 (m, 1H), 7.56 – 7.48 (m, 2H), 5.56 (s, 1H), 5.43 (s, 1H), 2.82 (t, *J* = 6.9 Hz, 2H), 2.66 – 2.61 (m, 2H), 2.47 (s, 3H), 2.02 – 1.95 (m, 2H). ^{13}C NMR (125 MHz, $CDCl_3$) δ 160.5, 155.2, 144.9, 137.2, 136.8, 136.0, 127.0, 126.6, 121.2, 112.7, 31.83, 29.9, 21.5, 18.5. LRMS (EI) *m/z* 226 $[M]^+$. HRMS (EI) calcd for $C_{14}H_{14}N_2O$ $[M]^+$ 226.1106, found 226.1099.

6,7,8,9-Tetrahydro-3-methyl-9-methylene-11*H*-pyrido[2,1-*b*]quinazolin-11-one (7C):

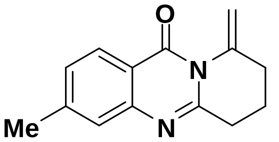

81 mg, 89% yield. ^{1}H NMR (400 MHz, $CDCl_3$) δ 8.17 (d, *J* = 8.2 Hz, 1H), 7.44 (s, 1H), 7.28 – 7.25 (m, 1H), 5.57 (d, *J* = 0.5 Hz, 1H), 5.44 (d, *J* = 0.5 Hz, 1H), 2.86 (t, *J* = 6.9 Hz, 2H), 2.66 – 2.61 (m, 2H), 2.48 (s, 3H), 1.99 (ddd, *J* = 11.4, 10.3, 7.1 Hz, 2H). ^{13}C NMR (100 MHz, $CDCl_3$) δ160.2, 156.4, 145.8, 136.9, 128.4, 127.5, 126.2, 118.9, 112.8, 31.6, 29.8, 22.1, 18.4. LRMS (EI) *m/z* 226 $[M]^+$. HRMS (EI) calcd for $C_{14}H_{14}N_2O$ $[M]^+$ 226.1106, found 226.1094.

6,7,8,9-Tetrahydro-2-methoxy-9-methylene-11*H*-pyrido[2,1-*b*]quinazolin-11-one (7D):

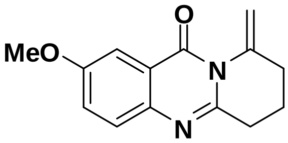

90 mg, 93% yield. ^{1}H NMR (500 MHz, $CDCl_3$) δ 7.65 (d, *J* = 2.9 Hz, 1H), 7.53 (d, *J* = 8.9 Hz, 1H), 7.31 (dd, *J* = 8.9, 2.9 Hz, 1H), 5.57 (s, 1H), 5.44 (s, 1H), 3.90 (s, 3H), 2.81 (t, *J* = 6.9 Hz, 2H), 2.67 – 2.61 (m, 2H), 2.02 – 1.94 (m, 2H). ^{13}C NMR (125 MHz, $CDCl_3$) δ 160.4, 158.3, 153.7, 141.6, 137.3, 128.4, 124.7, 122.2, 112.7, 106.9, 56.0, 31.7, 29.8, 18.5. LRMS (EI) *m/z* 242 $[M]^+$. HRMS (EI) calcd for $C_{14}H_{14}N_2O_2$ $[M]^+$ 242.1055, found 242.1046.

6,7,8,9-Tetrahydro-1-fluoro-9-methylene-11*H*-pyrido[2,1-*b*]quinazolin-11-one (7E):

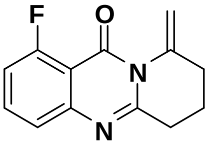

76 mg, 82% yield, off-white solid, m. p. 85.1-87.9 °C. ^{1}H NMR (400 MHz, $CDCl_3$) δ 7.63 (td, *J* = 8.2, 5.4 Hz, 1H), 7.39 (d, *J* = 8.2 Hz, 1H), 7.08 (ddd, *J* = 10.7, 8.2, 0.9 Hz, 1H), 5.58 (s, 1H), 5.44 (s, 1H), 2.80 (t, *J* = 7.0 Hz, 2H), 2.68 – 2.59 (m, 2H), 1.99 (ddd, *J* = 11.5, 10.4, 7.1 Hz, 2H). ^{13}C NMR (100 MHz, $CDCl_3$) δ 161.9 (d, *J* = 266.2 Hz), 157.5 (d, *J* = 4.3 Hz), 157.1, 149.1, 136.4, 135.0 (d, *J* = 10.6 Hz), 122.7 (d, *J* = 4.1 Hz), 113.4 (d, *J* = 21.2 Hz), 113.1, 111.2 (d, *J* = 5.4 Hz), 31.8, 29.8, 18.4. LRMS (EI) *m/z* 230 $[M]^+$. HRMS (EI) calcd for $C_{13}H_{11}FN_2O$ $[M]^+$ 230.0855, found 230.0839.

6,7,8,9-Tetrahydro-1-chloro-9-methylene-11*H*-pyrido[2,1-*b*]quinazolin-11-one (7F):

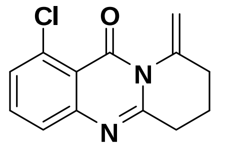

82 mg, 83% yield, off-white solid, m. p. 87.9-89 °C. ^{1}H NMR (400 MHz, $CDCl_3$) δ 7.58 – 7.53 (m, 1H), 7.50 (dd, *J* = 8.1, 1.4 Hz, 1H), 7.43 (dd, *J* = 7.5, 1.5 Hz, 1H), 5.58 (s, 1H), 5.45 (s, 1H), 2.79 (t, *J* = 7.0 Hz, 2H), 2.65 – 2.60 (m, 2H), 2.02 – 1.94 (m, 2H). ^{13}C NMR (100 MHz, $CDCl_3$) δ 158.4, 156.8, 149.4, 136.6, 134.9, 133.9, 129.6, 126.1, 118.6, 113.2, 31.7, 29.9, 18.4. LRMS (EI) *m/z* 246 $[M]^+$. HRMS (EI) calcd for $C_{13}H_{11}ClN_2O$ $[M]^+$ 246.0560, found 246.0547.

6,7,8,9-Tetrahydro-2-chloro-9-methylene-11*H*-pyrido[2,1-*b*]quinazolin-11-one (7G):

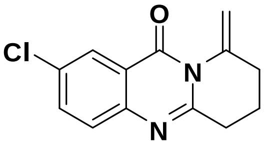

83 mg, 84% yield, off-white solid. ^{1}H NMR (500 MHz, $CDCl_3$) δ 8.22 (t, *J* = 2.4 Hz, 1H), 7.62 (dt, *J* = 8.6, 2.2 Hz, 1H), 7.52 (dd, *J* = 8.7, 1.8 Hz, 1H), 5.56 (s, 1H), 5.44 (s, 1H), 2.80 (td, *J* = 6.9, 1.4 Hz, 2H), 2.65 – 2.60 (m, 2H), 2.01 – 1.95 (m, 2H). ^{13}C NMR (125 MHz, $CDCl_3$) δ 159.5, 156.2, 145.53, 136.9, 134.9, 132.3, 128.5, 126.8, 122.5, 112.9, 31.9, 29.7, 18.4. LRMS (EI) *m/z* 246 $[M]^+$. HRMS (EI) calcd for $C_{13}H_{11}ClN_2O$ $[M]^+$ 246.0560, found 246.0543.

6,7,8,9-Tetrahydro-3-chloro-9-methylene-11*H*-pyrido[2,1-*b*]quinazolin-11-one (7H):

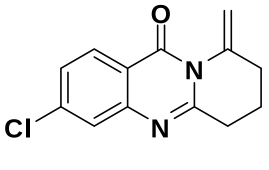

84 mg, 85% yield, off-white solid. ^{1}H NMR (500 MHz, $CDCl_3$) δ 8.21 (d, *J* = 8.5 Hz, 1H), 7.60 (d, *J* = 1.9 Hz, 1H), 7.38 (dd, *J* = 8.5, 2.0 Hz, 1H), 5.57 (s, 1H), 5.45 (s, 1H), 2.82 (t, *J* = 6.9 Hz, 2H), 2.67 – 2.61 (m, 2H), 2.00 (dt, *J* = 14.6, 7.1 Hz, 2H). ^{13}C NMR (125 MHz, $CDCl_3$) δ 159.9, 157.3,

148.0, 140.7, 136.9, 129.1, 127.3, 126.5, 120.0, 112.9, 32.0, 29.8, 18.4. LRMS (EI) *m/z* 246 $[M]^+$. HRMS (EI) calcd for $C_{13}H_{11}ClN_2O$ 246.0560, found 246.0552.

6,7,8,9-Tetrahydro-2-bromo-9-methylene-11*H*-pyrido[2,1-*b*]quinazolin-11-one (7I):

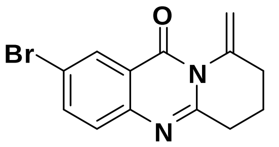

100 mg, 86% yield, off-white solid. ^{1}H NMR (400 MHz, $CDCl_3$) δ 8.39 (d, *J* = 2.3 Hz, 1H), 7.77 (ddd, *J* = 8.7, 2.3, 0.9 Hz, 1H), 7.46 (d, *J* = 8.7 Hz, 1H), 5.57 – 5.55 (m, 1H), 5.46 – 5.44 (m, 1H), 2.80 (t, *J* = 6.9 Hz, 2H), 2.67 – 2.61 (m, 2H), 2.03 – 1.95 (m, 2H). ^{13}C NMR (125 MHz, $CDCl_3$) δ 159.3, 156.4, 145.9, 137.7, 136.9, 130.1, 128.7, 122.9, 120.0, 113.0, 32.0, 29.7, 18.4. LRMS (EI) *m/z* 290 $[M]^+$. HRMS (EI) calcd for $C_{13}H_{11}BrN_2O$ $[M]^+$ 290.0055, found 290.0050.

6,7,8,9-Tetrahydro-3-phenyl-9-methylene-11*H*-pyrido[2,1-*b*]quinazolin-11-one (7J):

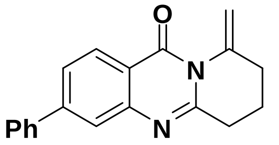

103 mg, 89% yield, off-white solid. ^{1}H NMR (500 MHz, $CDCl_3$) δ 8.32 – 8.24 (m, 1H), 7.83 – 7.74 (m, 1H), 7.68 – 7.58 (m, 3H), 7.47 – 7.38 (m, 2H), 7.38 – 7.31 (m, 1H), 5.59 – 5.52 (m, 1H), 5.43 – 5.37 (m, 1H), 2.83 – 2.75 (m, 2H), 2.66 – 2.54 (m, 2H), 2.02 – 1.88 (m, 2H). ^{13}C NMR (125 MHz, $CDCl_3$) δ 160.1, 156.1, 147.2, 147.0, 139.5, 136.8, 129.0, 128.4, 127.9, 127.3, 125.4, 124.6, 120.0, 112.5, 31.8, 29.6, 18.3. LRMS (EI) *m/z* 288 $[M]^+$. HRMS (EI) calcd for $C_{19}H_{16}N_2O$ $[M]^+$ 288.1263, found 288.1235.

1,2,3,4-Tetrahydro-1-methylene-12*H*-benzo[*g*]pyrido[2,1-*b*]quinazolin-12-one (7K):

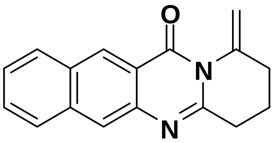

91 mg, 87% yield, off-white solid. ^{1}H NMR (500 MHz, $CDCl_3$) δ 8.92 (s, 1H), 8.09 (s, 1H), 8.05 (d, *J* = 8.3 Hz, 1H), 7.96 (d, *J* = 8.3 Hz, 1H), 7.60 (t, *J* = 7.5 Hz, 1H), 7.52 (t, *J* = 7.4 Hz, 1H), 5.59 (s, 1H), 5.46 (s, 1H), 2.88 (t, *J* = 6.9 Hz, 2H), 2.72 – 2.66 (m, 2H), 2.04 (p, *J* = 7.1 Hz, 2H). ^{13}C NMR (125 MHz, $CDCl_3$) δ 160.9, 155.2, 142.3, 137.2, 136.9, 131.6, 129.6, 129.3, 128.7, 128.1, 126.4, 124.4, 120.3, 112.6, 32.1, 29.9, 18.5. LRMS (EI) *m/z* 262 $[M]^+$. HRMS (EI) calcd for $C_{17}H_{14}N_2O$ $[M]^+$ 262.1106, found 262.1091.

6,7,8,9-Tetrahydro-6-methylene-4*H*-pyrido[1,2-*a*]thieno[2,3-*d*]pyrimidin-4-one (7L):

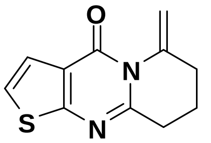

103 mg, 89% yield, pale yellow solid. ^{1}H NMR (500 MHz, $CDCl_3$) δ 7.48 (d, *J* = 5.8 Hz, 1H), 7.16 (d, *J* = 5.8 Hz, 1H), 5.57 (s, 1H), 5.47 (s, 1H), 2.83 (t, *J* = 7.0 Hz, 2H), 2.66 – 2.61 (m, 2H), 2.03 – 1.95 (m, 2H). ^{13}C NMR (125 MHz, $CDCl_3$) δ 162.9, 157.1, 156.7, 136.8, 123.3, 123.1, 122.5, 113.4, 31.8, 29.9, 18.5. LRMS (EI) *m/z* 218 $[M]^+$; HRMS (EI) calcd for $C_{11}H_{10}N_2OS$ $[M]^+$ 218.0514, found 218.0500.

2-(3-Butynyl)-4(3*H*)-quinazolinone (8A):

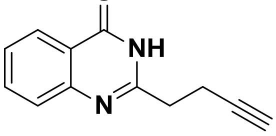

^{1}H NMR (500 MHz, DMSO-d_6) δ 12.23 (s, 1H), 8.08 (dd, *J* = 7.9, 1.2 Hz, 1H), 7.81 – 7.74 (m, 1H), 7.61 (d, *J* = 8.1 Hz, 1H), 7.50 – 7.44 (m, 1H), 2.84 – 2.79 (m, 3H), 2.65 (td, *J* = 7.5, 2.6 Hz,

2H).^{13}C NMR (125 MHz, DMSO-d_6) δ 161.7, 155.6, 148.7, 134.4, 126.9, 126.2, 125.7, 120.9, 83.2, 72.0, 33.3, 15.5. LRMS (EI) *m/z* 198 $[M]^+$; HRMS (EI) calcd for $C_{12}H_{10}N_2O$ $[M]^+$198.0793, found 198.0787.

2-(3-Butynyl)-6-methyl-4(3*H*)-quinazolinone (8B):

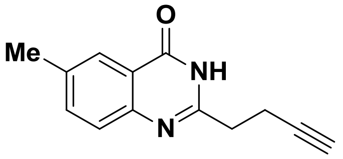

^{1}H NMR (500 MHz, DMSO-d_6) δ 12.15 (s, 1H), 7.87 (br s, 1H), 7.59 (dd, *J* = 8.3, 1.8 Hz, 1H), 7.50 (d, *J* = 8.3 Hz, 1H), 2.82 (t, *J* = 2.6 Hz, 1H), 2.79 (t, *J* = 7.4 Hz, 2H), 2.63 (td, *J* = 7.4, 2.4 Hz, 2H), 2.41 (s, 3H). ^{13}C NMR (125 MHz, DMSO-d_6) δ 161.7, 154.7, 146.8, 135.8, 135.7, 126.8, 125.2, 120.7, 83.3, 72.0, 33.2, 20.8, 15.5. LRMS (ESI) *m/z* 213 $[M+H]^+$; HRMS (ESI) calcd for $C_{13}H_{13}N_2O$ $[M+H]^+$ 213.1028, found 213.1022.

2-(3-Butynyl)-7-methyl-4(3*H*)-quinazolinone (8C):

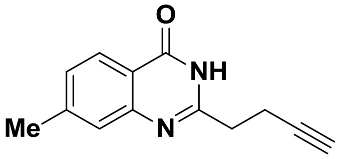

^{1}H NMR (500 MHz, DMSO-d_6) δ 12.13 (s, 1H), 7.96 (d, *J* = 8.1 Hz, 1H), 7.42 (s, 1H), 7.29 (d, *J* = 8.0 Hz, 1H), 2.82 – 2.77 (m, 3H), 2.64 (td, *J* = 7.3, 2.2 Hz, 2H), 2.43 (s, 3H).^{13}C NMR (125 MHz, DMSO-d_6) δ 161.6, 155.6, 148.9, 144.9, 127.6, 126.6, 125.6, 118.5, 83.2, 72.0, 33.3, 21.4, 15.5. LRMS (EI) *m/z* 212 $[M]^+$; HRMS (EI) calcd for $C_{13}H_{12}N_2O$ $[M]^+$ 212.0950, found 212.0943.

2-(3-Butynyl)-6-methoxy-4(3*H*)-quinazolinone (8D):

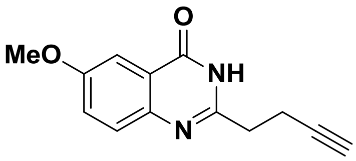

^{1}H NMR (400 MHz, DMSO-d_6) δ 12.18 (s, 1H), 7.56 (d, *J* = 8.9 Hz, 1H), 7.46 (d, *J* = 2.9 Hz, 1H), 7.37 (dd, *J* = 8.9, 2.9 Hz, 1H), 3.85 (s, 3H), 2.83 – 2.74 (m, 3H), 2.63 (td, *J* = 7.4, 2.2 Hz, 2H).^{13}C NMR (100 MHz, DMSO-d_6) δ 161.6, 157.4, 153.2, 143.2, 128.6, 123.9, 121.7, 105.8, 83.3, 72.0, 55.6, 33.1, 15.5. LRMS (ESI) *m/z* 229 [M+H]$^+$; HRMS (ESI) calcd for $C_{13}H_{13}N_2O_2$ [M+H]$^+$ 229.0977, found 229.0972.

2-(3-Butynyl)-8-methoxy-4(3*H*)-quinazolinone (8E):

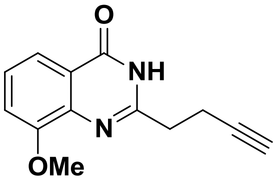

^{1}H NMR (500 MHz, DMSO-d_6) δ 12.24 (s, 1H), 7.63 (dd, *J* = 7.9, 1.3 Hz, 1H), 7.38 (d, *J* = 7.9 Hz, 1H), 7.32 (dd, *J* = 8.0, 1.0 Hz, 1H), 3.89 (s, 3H), 2.84 – 2.77 (m, 3H), 2.64 (td, *J* = 7.5, 2.6 Hz, 2H). ^{13}C NMR (125 MHz, DMSO-d_6) δ 161.6, 154.3, 139.3, 126.3, 121.9, 116.8, 115.0, 83.1, 72.0, 55.9, 33.5, 15.8. LRMS (ESI) *m/z* 229 [M+H]$^+$; HRMS (ESI) calcd for $C_{13}H_{12}N_2O_2$ [M+Na]$^+$ 251.0796, found 251.0802.

2-(3-Butynyl)-5-fluoro-4(3*H*)-quinazolinone (8F):

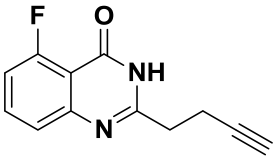

^{1}H NMR (400 MHz, DMSO-d_6) δ 12.28 (s, 1H), 7.74 (td, *J* = 8.2, 5.7 Hz, 1H), 7.41 (d, *J* = 8.2 Hz, 1H), 7.26 – 7.14 (m, 1H), 2.83 (t, *J* = 2.7 Hz, 1H), 2.79 (t, *J* = 7.5 Hz, 2H), 2.63 (td, *J* = 7.4, 2.7 Hz, 2H). ^{13}C NMR (125 MHz, DMSO-d_6) δ 160.5 (d, *J* = 262.4 Hz), 159.0, 156.8, 150.9, 135.0 (d,

J = 10.6 Hz), 123.0, 112.6 (d, J = 20.4 Hz), 110.4 (d, J = 6.1 Hz), 83.1, 72.0, 33.1, 15.3. LRMS (EI) m/z 216 $[M]^+$; HRMS (EI) calcd for $C_{12}H_9FN_2O$ $[M]^+$ 216.0699, found 216.0691.

2-(3-Butynyl)-5-chloro-4(3*H*)-quinazolinone (8G):

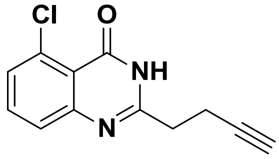

^{1}H NMR (500 MHz, DMSO-d_6) δ 12.28 (s, 1H), 7.69 (t, J = 8.0 Hz, 1H), 7.54 (dd, J = 8.2, 1.1 Hz, 1H), 7.47 (dd, J = 7.8, 1.1 Hz, 1H), 2.82 (t, J = 2.6 Hz, 1H), 2.79 (t, J = 7.4 Hz, 2H), 2.63 (td, J = 7.4, 2.4 Hz, 2H). ^{13}C NMR (125 MHz, DMSO-d_6) δ 159.8, 156.5, 151.3, 134.2, 132.4, 128.6, 126.5, 117.9, 83.0, 72.0, 33.0, 15.3. LRMS (EI) m/z 232 $[M]^+$; HRMS (EI) calcd for $C_{12}H_9ClN_2O$ $[M]^+$ 232.0403, found 232.0397.

2-(3-Butynyl)-6-chloro-4(3*H*)-quinazolinone (8H):

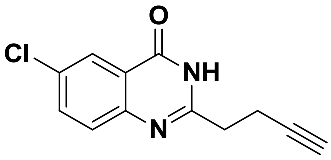

^{1}H NMR (500 MHz, DMSO-d_6) δ 12.41 (s, 1H), 8.00 (d, J = 2.5 Hz, 1H), 7.79 (dd, J = 8.7, 2.5 Hz, 1H), 7.63 (d, J = 8.7 Hz, 1H), 2.84 – 2.79 (m, 3H), 2.64 (td, J = 7.4, 2.6 Hz, 2H). ^{13}C NMR (125 MHz, DMSO-d_6) δ 160.7, 156.3, 147.4, 134.5, 130.4, 129.2, 124.8, 122.2, 83.1, 72.0, 33.3, 15.4.LRMS (ESI) m/z 233 $[M+H]^+$; HRMS (ESI) calcd for $C_{12}H_{10}N_2OCl$ $[M+H]^+$ 233.0482, found 233.0475.

2-(3-Butynyl)-7-chloro-4(3*H*)-quinazolinone (8I):

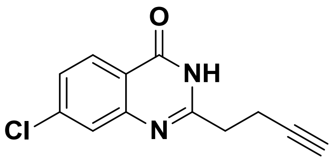

^{1}H NMR (500 MHz, DMSO-d_6) δ 12.38 (s, 1H), 8.07 (d, *J* = 8.5 Hz, 1H), 7.66 (d, *J* = 2.0 Hz, 1H), 7.50 (dd, *J* = 8.5, 2.1 Hz, 1H), 2.85 – 2.79 (m, 3H), 2.64 (td, *J* = 7.4, 2.5 Hz, 2H). ^{13}C NMR (125 MHz, DMSO-d_6) δ 161.1, 157.3, 149.8, 139.0, 127.9, 126.5, 126.1, 119.7, 83.0, 72.1, 33.3, 15.4.LRMS (EI) *m/z* 232 [M]$^+$; HRMS (EI) calcd for $C_{12}H_9ClN_2O$ [M]$^+$ 232.0403, found 232.0391.

2-(3-Butynyl)-6-bromo-4(3*H*)-quinazolinone (8J):

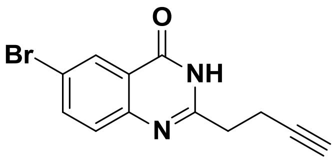

^{1}H NMR (400 MHz, DMSO-d_6) δ 12.43 (s, 1H), 8.15 (d, *J* = 2.3 Hz, 1H), 7.91 (dd, *J* = 8.7, 2.4 Hz, 1H), 7.56 (d, *J* = 8.7 Hz, 1H), 2.84 – 2.78 (m, 3H), 2.64 (td, *J* = 7.4, 2.6 Hz, 2H). ^{13}C NMR (100 MHz, DMSO-d_6) δ 160.6, 156.4, 147.7, 137.2, 129.4, 127.9, 122.6, 118.6, 83.1, 72.1, 33.3, 15.4. LRMS (ESI) *m/z* 277 [M+H]$^+$; HRMS (ESI) calcd for $C_{12}H_9N_2OBrNa$ [M+Na]$^+$ 298.9796, found 298.9792.

2-(3-Butynyl)-7-phenyl-4(3*H*)-quinazolinone (8K):

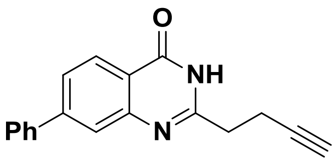

^{1}H NMR (400 MHz, DMSO-d_6) δ 12.26 (s, 1H), 8.15 (d, *J* = 8.3 Hz, 1H), 7.89 – 7.83 (m, 1H), 7.83 – 7.65 (m, 3H), 7.52 (t, *J* = 7.4 Hz, 2H), 7.44 (t, *J* = 7.2 Hz, 1H), 2.88 – 2.80 (m, 3H), 2.68 (td, *J* = 7.3, 2.1 Hz, 1H). ^{13}C NMR (100 MHz, DMSO- d_6) δ 161.5, 156.1, 149.3, 146.0, 138.8,

129.2, 128.6, 127.2, 126.5, 124.9, 124.4, 119.8, 83.2, 72.1, 33.4, 15.5. LRMS (ESI) *m/z* 275 $[M+H]^+$; HRMS (ESI) calcd for $C_{18}H_{15}N_2O$ $[M+H]^+$ 275.1184, found 275.1187.

2-(3-Butynyl)-4(3*H*)-benzo[*g*]quinazolinone (8L):

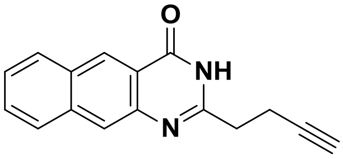

^{1}H NMR (400 MHz, DMSO-d_6) δ 12.06 (s, 1H), 8.79 (s, 1H), 8.23 – 8.12 (m, 2H), 8.06 (d, *J* = 7.7 Hz, 1H), 7.69 – 7.60 (m, 1H), 7.60 – 7.51 (m, 1H), 2.89 – 2.79 (m, 3H), 2.69 (t, *J* = 7.7 Hz, 2H). ^{13}C NMR (100 MHz, DMSO-d_6) δ 162.1, 154.6, 144.1, 136.3, 130.7, 129.3, 128.5, 127.8, 127.2, 126.1, 124.2, 120.2, 83.3, 72.0, 33.4, 15.3. LRMS (ESI) *m/z* 249 $[M+H]^+$; HRMS (ESI) calcd for $C_{16}H_{13}N_2O$ $[M+H]^+$ 249.1028, found 249.1021.

2,3-Dihydro-1-methylene-pyrrolo[2,1-*b*]quinazolin-9(1*H*)-one (9A):

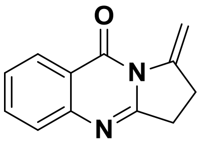

74 mg, 93% yield, white solid. ^{1}H NMR (500 MHz, $CDCl_3$) δ 8.31 (d, *J* = 8.0 Hz, 1H), 7.75 – 7.69 (m, 1H), 7.62 (d, *J* = 8.2 Hz, 1H), 7.46 (t, *J* = 7.6 Hz, 1H), 6.43 (s, 1H), 5.02 (s, 1H), 3.14 – 3.09 (m, 2H), 2.92 – 2.86 (m, 2H). ^{13}C NMR (125 MHz, $CDCl_3$) δ 161.1, 159.2, 147.6, 143.5, 134.6, 127.1, 126.9, 126.9, 121.4, 100.8, 29.2, 26.1. LRMS (EI) *m/z* 198 $[M]^+$. HRMS (EI) calcd for $C_{12}H_{10}N_2O$ $[M]^+$ 198.0793, found 198.0789.

2,3-Dihydro-1-methylene-7-methyl-pyrrolo[2,1-*b*]quinazolin-9(1*H*)-one (9B):

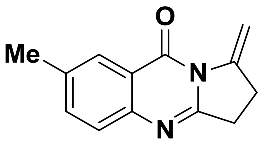

76 mg, 89% yield, off-white solid. ^{1}H NMR (400 MHz, $CDCl_3$) δ 8.09 (s, 1H), 7.55 – 7.48 (m, 2H), 6.43 – 6.40 (m, 1H), 5.01 – 4.98 (m, 1H), 3.12 – 3.05 (m, 2H), 2.91 – 2.84 (m, 2H), 2.47 (s, 3H). ^{13}C NMR (100 MHz, $CDCl_3$) δ 161.1, 158.3, 145.5, 143.5, 137.0, 136.0, 126.6, 126.5, 121.1, 100.6, 29.0, 26.1, 21.5. LRMS (EI) *m/z* 212 $[M]^+$. HRMS (EI) calcd for $C_{13}H_{12}N_2O$ $[M]^+$ 212.0950, found 212.0947.

2,3-Dihydro-1-methylene-6-methyl-pyrrolo[2,1-*b*]quinazolin-9(1*H*)-one (9C):

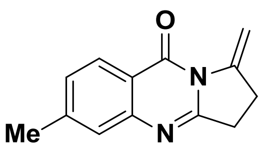

77 mg, 90% yield, off-white solid. ^{1}H NMR (500 MHz, $CDCl_3$) δ 8.20 (d, *J* = 8.1 Hz, 1H), 7.41 (s, 1H), 7.28 (d, *J* = 8.2 Hz, 1H), 6.42 (s, 1H), 5.00 (s, 1H), 3.17 – 3.05 (m, 2H), 2.95 – 2.82 (m, 2H), 2.49 (s, 3H). ^{13}C NMR (125 MHz, $CDCl_3$) δ 161.1, 159.3, 147.7, 145.7, 143.5, 128.46, 127.0, 126.7, 119.0, 100.6, 29.2, 26.1, 22.1. LRMS (EI) *m/z* 212 $[M]^+$. HRMS (EI) calcd for $C_{13}H_{12}N_2O$ $[M]^+$ 212.0950, found 212.0950.

2,3-Dihydro-1-methylene-7-methoxy-pyrrolo[2,1-*b*]quinazolin-9(1*H*)-one (9D):

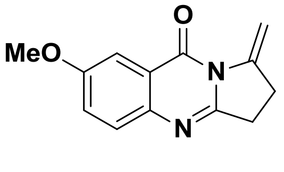

84 mg, 84% yield, off-white solid. ^{1}H NMR (500 MHz, $CDCl_3$) δ 7.68 (d, *J* = 2.9 Hz, 1H), 7.55 (d, *J* = 8.9 Hz, 1H), 7.31 (dd, *J* = 8.9, 3.0 Hz, 1H), 6.43 (s, 1H), 5.02 (s, 1H), 3.91 (s, 3H), 3.12 – 3.07 (m, 2H), 2.92 – 2.86 (m, 2H). ^{13}C NMR (125 MHz, $CDCl_3$) δ 160.9, 158.5, 156.9, 143.6, 142.0,

128.3, 124.6, 122.1, 106.6, 100.8, 56.0, 28.9, 26.2. LRMS (EI) *m/z* 228 $[M]^+$. HRMS (EI) calcd for $C_{13}H_{12}N_2O_2$ $[M]^+$ 228.0899, found 228.0899.

2,3-Dihydro-1-methylene-5-methoxy-pyrrolo[2,1-*b*]quinazolin-9(1*H*)-one (9E):

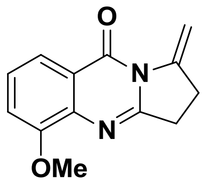

82 mg, 90% yield, off-white solid, m. p. 187.3-190.5 °C. ^1H NMR (500 MHz, $CDCl_3$) δ 7.90 (dd, *J* = 8.1, 0.9 Hz, 1H), 7.39 (t, *J* = 8.0 Hz, 1H), 7.19 (d, *J* = 7.9 Hz, 1H), 6.44 (s, 1H), 5.02 (s, 1H), 4.01 (s, 3H), 3.20 – 3.16 (m, 2H), 2.88 (dt, *J* = 14.5, 4.8 Hz, 2H). ^{13}C NMR (125 MHz, $CDCl_3$) δ 160.9, 158.6, 154.2, 143.6, 138.0, 127.0, 122.6, 118.4, 114.4, 101.0, 56.5, 29.5, 26.1. LRMS (EI) *m/z* 228 $[M]^+$. HRMS (EI) calcd for $C_{13}H_{12}N_2O_2$ $[M]^+$ 228.0899, found 228.0902.

2,3-Dihydro-1-methylene-8-fluoro-pyrrolo[2,1-*b*]quinazolin-9(1*H*)-one (9F):

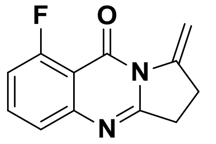

69 mg, 80% yield, off-white solid. ^1H NMR (500 MHz, $CDCl_3$) δ 7.64 (td, *J* = 8.2, 5.4 Hz, 1H), 7.41 (d, *J* = 8.2 Hz, 1H), 7.10 (dd, *J* = 10.7, 8.3 Hz, 1H), 6.43 (s, 1H), 5.01 (s, 1H), 3.13 – 3.05 (m, 2H), 2.93 – 2.85 (m, 2H). ^{13}C NMR (125 MHz, $CDCl_3$) δ 162.2 (d, *J* = 266.0 Hz), 160.2, 158.2 (d, *J* = 3.8 Hz), 149.8, 143.1, 135.0 (d, *J* = 10.7 Hz), 122.8 (d, *J* = 4.1 Hz), 113.7 (d, *J* = 21.2 Hz), 111.0 (d, *J* = 5.6 Hz), 101.2, 29.1, 26.0. LRMS (EI) *m/z* 216 $[M]^+$. HRMS (EI) calcd for $C_{12}H_9FN_2O$ $[M]^+$ 216.0699, found 216.0701.

2,3-Dihydro-1-methylene-8-chloro-pyrrolo[2,1-*b*]quinazolin-9(1*H*)-one (9G):

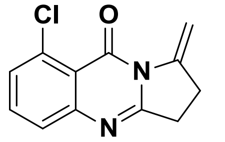

76 mg, 82% yield, off-white solid, m. p. 178.9-181.3 °C. ^{1}H NMR (500 MHz, $CDCl_3$) δ 7.55 (t, *J* = 7.9 Hz, 1H), 7.50 (d, *J* = 7.3 Hz, 1H), 7.43 (d, *J* = 7.6 Hz, 1H), 6.43 (s, 1H), 5.00 (s, 1H), 3.10 – 3.03 (m, 2H), 2.88 (t, *J* = 8.1 Hz, 2H). ^{13}C NMR (125 MHz, $CDCl_3$) δ 159.8, 159.1, 150.1, 143.2, 134.8, 133.9, 129.8, 126.3, 118.4, 101.3, 29.0, 26.1. LRMS (ESI) *m/z* 233 ([M+H]$^+$). HRMS (ESI) calcd for $C_{12}H_9ClN_2ONa$ [M+Na]$^+$ 255.0301, found 255.0303.

2,3-Dihydro-1-methylene-7-chloro-pyrrolo[2,1-*b*]quinazolin-9(1*H*)-one (9H):

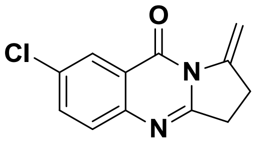

78 mg, 84% yield, off-white solid. ^{1}H NMR (500 MHz, $CDCl_3$) δ 8.43 (d, *J* = 2.3 Hz, 1H), 7.79 (dd, *J* = 8.6, 2.3 Hz, 1H), 7.49 (d, *J* = 8.6 Hz, 1H), 6.42 (s, 1H), 5.04 (s, 1H), 3.12 – 3.07 (m, 2H), 2.89 (t, *J* = 8.1 Hz, 2H). ^{13}C NMR (125 MHz, $CDCl_3$) δ 159.8, 159.6, 146.5, 143.3, 137.7, 129.66, 128.7, 122.9, 120.4, 101.3, 29.2, 26.0. LRMS (EI) *m/z* 232 [M]$^+$. HRMS (EI) calcd for $C_{12}H_9ClN_2O$ [M]$^+$ 232.0403, found 232.0396.

2,3-Dihydro-1-methylene-6-chloro-pyrrolo[2,1-*b*]quinazolin-9(1*H*)-one (9I):

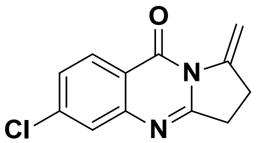

77 mg, 83% yield, off-white solid. ^{1}H NMR (400 MHz, $CDCl_3$) δ 8.23 (d, *J* = 8.5 Hz, 1H), 7.61 (d, *J* = 1.9 Hz, 1H), 7.41 (dd, *J* = 8.5, 1.9 Hz, 1H), 6.41 (d, *J* = 0.8 Hz, 1H), 5.03 (d, *J* = 0.9 Hz, 1H), 3.15 – 3.08 (m, 2H), 2.93 – 2.86 (m, 2H). ^{13}C NMR (125 MHz, $CDCl_3$) δ 160.6, 160.4, 148.6,

143.3, 140.8, 128.5, 127.5, 126.6, 119.9, 101.2, 29.3, 26.0. LRMS (EI) *m/z* 232 $[M]^+$. HRMS (EI) calcd for $C_{12}H_9ClN_2O$ $[M]^+$ 232.0403, found 232.0395.

2,3-Dihydro-1-methylene-7-bromo-pyrrolo[2,1-*b*]quinazolin-9(1*H*)-one (9J):

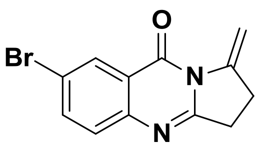

92 mg, 83% yield, off-white solid, m. p. 182.1-184.7 °C. ^1H NMR (400 MHz, $CDCl_3$) δ 8.44 (d, *J* = 2.3 Hz, 1H), 7.80 (dd, *J* = 8.6, 2.3 Hz, 1H), 7.49 (d, *J* = 8.6 Hz, 1H), 6.42 (s, 1H), 5.05 (s, 1H), 3.13 – 3.07 (m, 2H), 2.94 – 2.86 (m, 2H). ^{13}C NMR (100 MHz, $CDCl_3$) δ 159.8, 159.6, 146.5, 143.3, 137.7, 129.7, 128.7, 122.9, 120.4, 101.3, 29.2, 26.0. LRMS (EI) *m/z* 276 $[M]^+$. HRMS (EI) calcd for $C_{12}H_9BrN_2O$ $[M]^+$ 275.9898, found 275.9891.

2,3-Dihydro-1-methylene-6-phenyl-pyrrolo[2,1-*b*]quinazolin-9(1*H*)-one (9K):

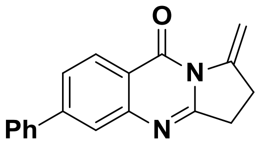

100 mg, 91% yield, off-white solid. ^1H NMR (400 MHz, $CDCl_3$) δ 8.36 (d, *J* = 8.3 Hz, 1H), 7.84 (s, 1H), 7.73 – 7.66 (m, 3H), 7.49 (t, *J* = 7.4 Hz, 2H), 7.42 (t, *J* = 7.2 Hz, 1H), 6.45 (s, 1H), 5.03 (s, 1H), 3.19 – 3.09 (m, 2H), 2.94 – 2.87 (m, 2H). ^{13}C NMR (100 MHz, $CDCl_3$) δ 161.0, 159.6, 148.0, 147.4, 143.5, 139.7, 129.2, 128.7, 127.6, 125.9, 124.9, 120.1, 100.8, 29.2, 26.1. LRMS (EI) *m/z* 274 $[M]^+$. HRMS (EI) calcd for $C_{18}H_{14}N_2O$ $[M]^+$ 274.1106, found 274.1108.

2,3-Dihydro-1-methylene-benzo[*g*]pyrrolo[2,1-*b*]quinazolin-11(1*H*)-one (9L):

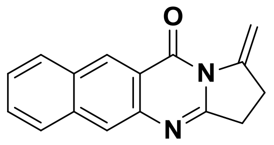

86 mg, 86% yield, off-white solid. ^{1}H NMR (400 MHz, $CDCl_3$) δ 8.92 (s, 1H), 8.08 (s, 1H), 8.05 (d, *J* = 8.3 Hz, 1H), 7.96 (d, *J* = 8.3 Hz, 1H), 7.63 – 7.58 (m, 1H), 7.56 – 7.50 (m, 1H), 6.41 (s, 1H), 5.02 (s, 1H), 3.18 – 3.11 (dd, *J* = 9.4, 7.1 Hz, 2H), 2.95 – 2.87 (m, 2H). ^{13}C NMR (100 MHz, $CDCl_3$) δ 161.4, 158.4, 143.4, 142.8, 136.8, 131.6, 129.6, 128.9, 128.8, 128.2, 126.5, 124.5, 120.1, 100.2, 29.2, 26.2. LRMS (EI) *m/z* 248 $[M]^+$. HRMS (EI) calcd for $C_{16}H_{12}N_2O$ $[M]^+$ 248.0950, found 248.0945.

Copies of ^{1}H NMR and ^{13}C NMR of compounds

2-(4-Pentynyl)-4(3*H*)-quinazolinone (6A)

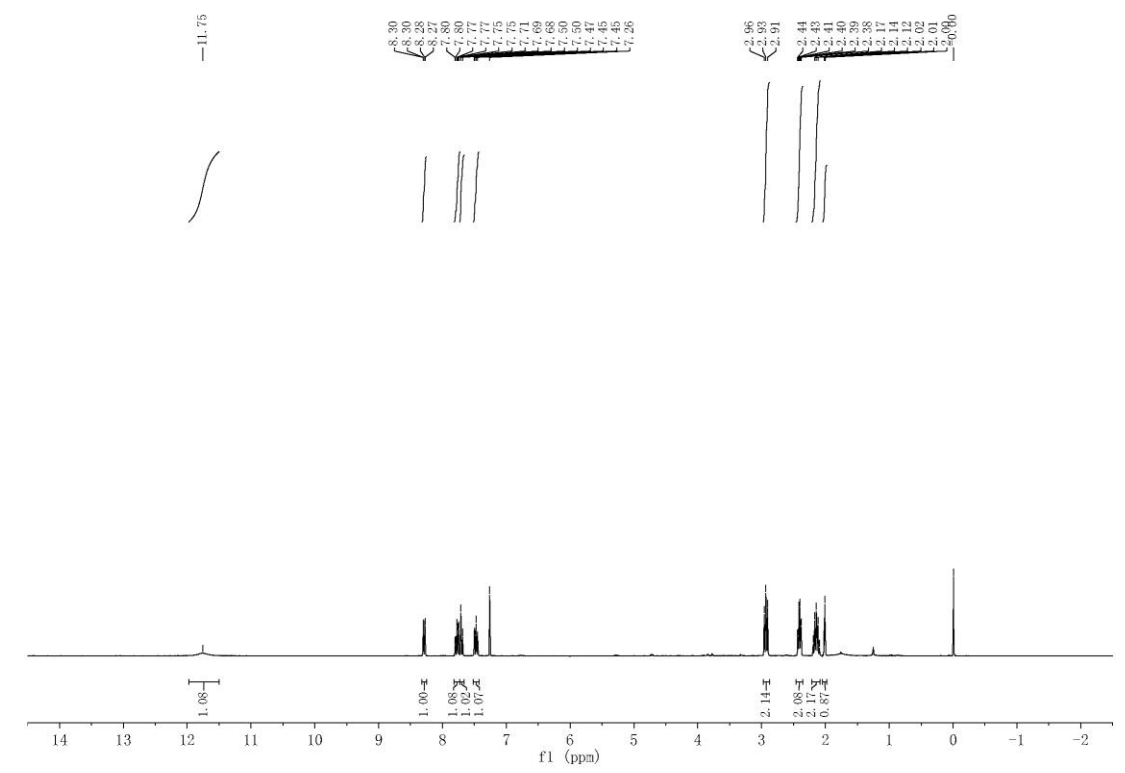

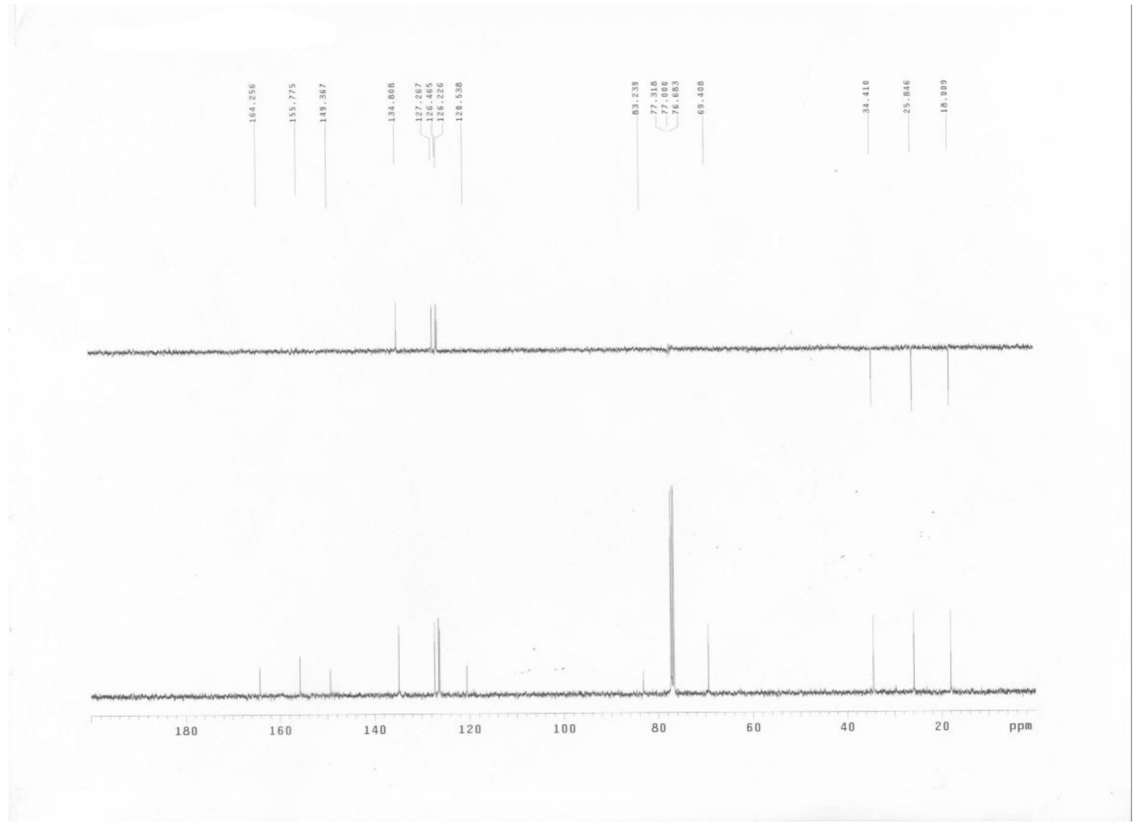

6,7,8,9-Tetrahydro-9-methylene-11*H*-pyrido[2,1-*b*]quinazolin-11-one (7A)

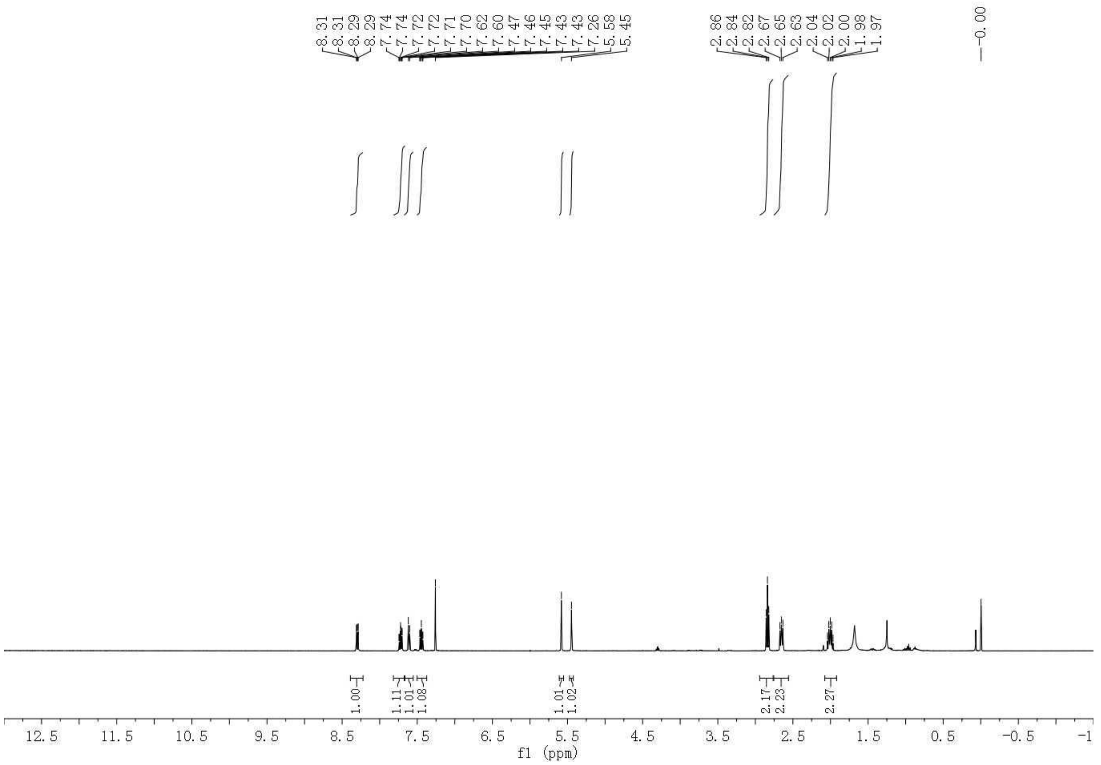

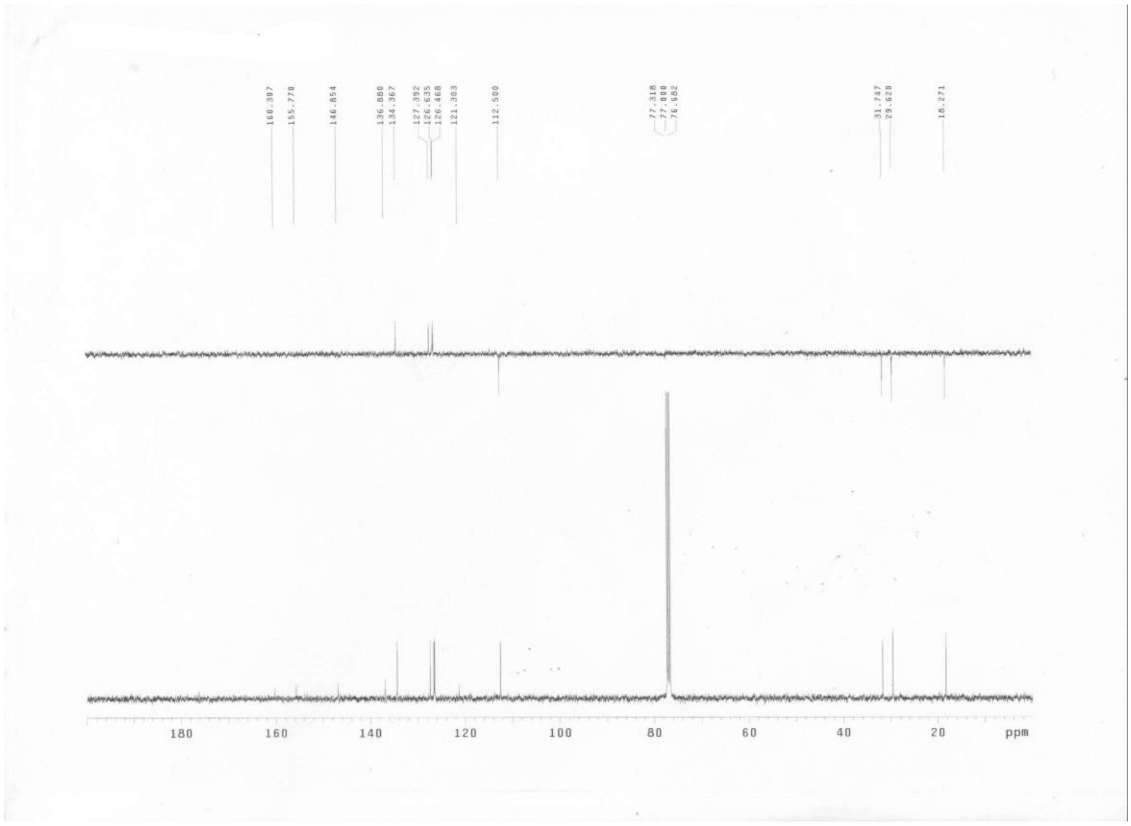

2-(4-Pentynyl)-6-methyl-4(3*H*)-quinazolinone (6B)

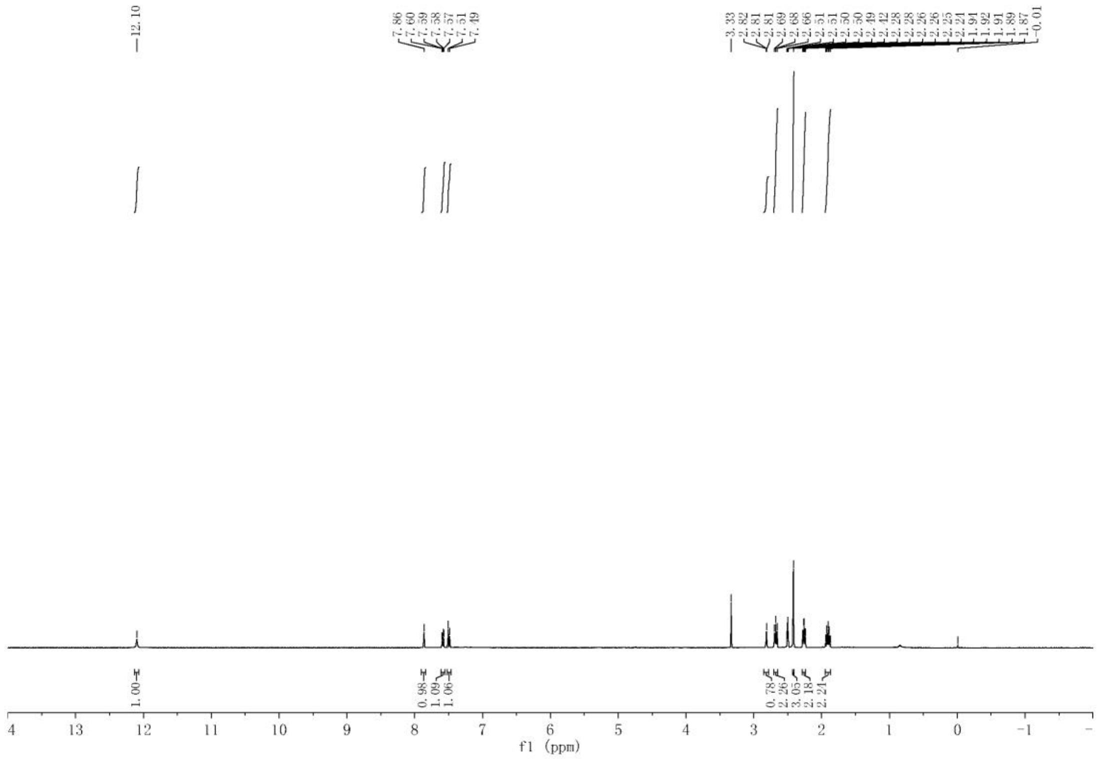

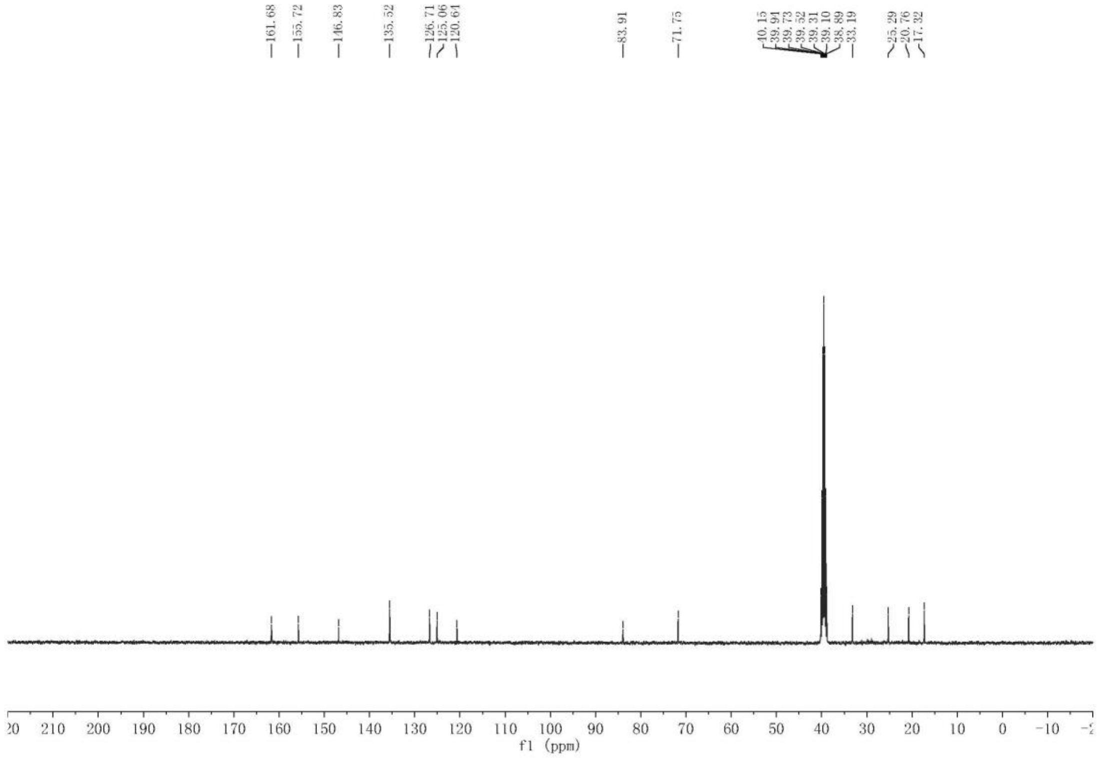

6,7,8,9-Tetrahydro-2-methyl-9-methylene-11*H*-pyrido[2,1-*b*]quinazolin-11-one (7B)

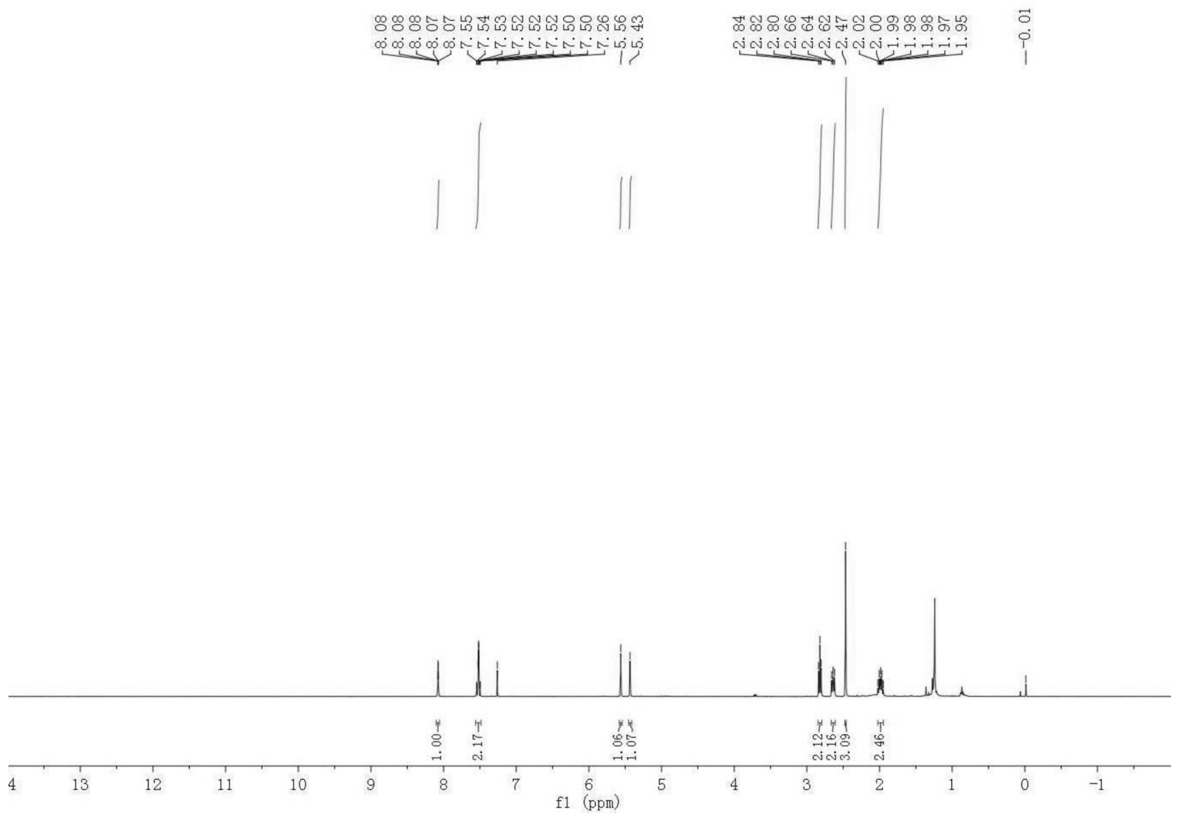

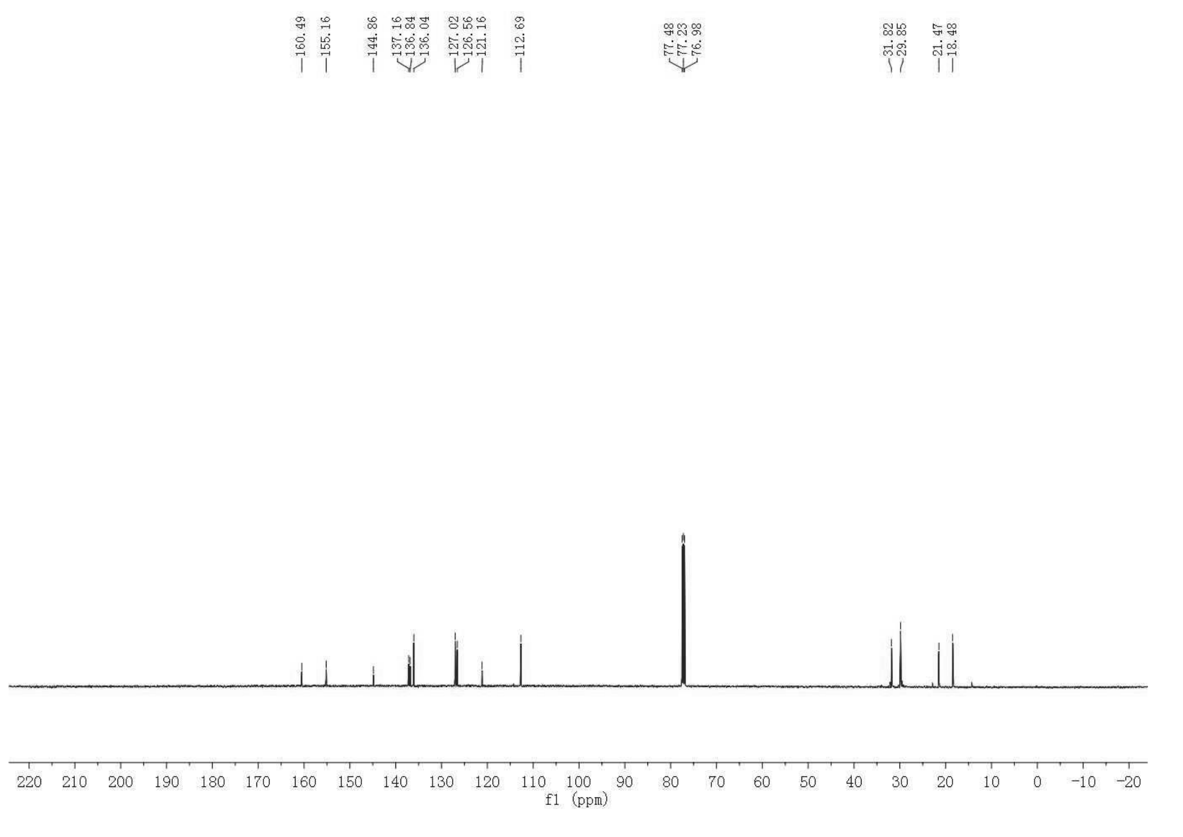

2-(4-Pentynyl)-7-methyl-4(3*H*)-quinazolinone (6C)

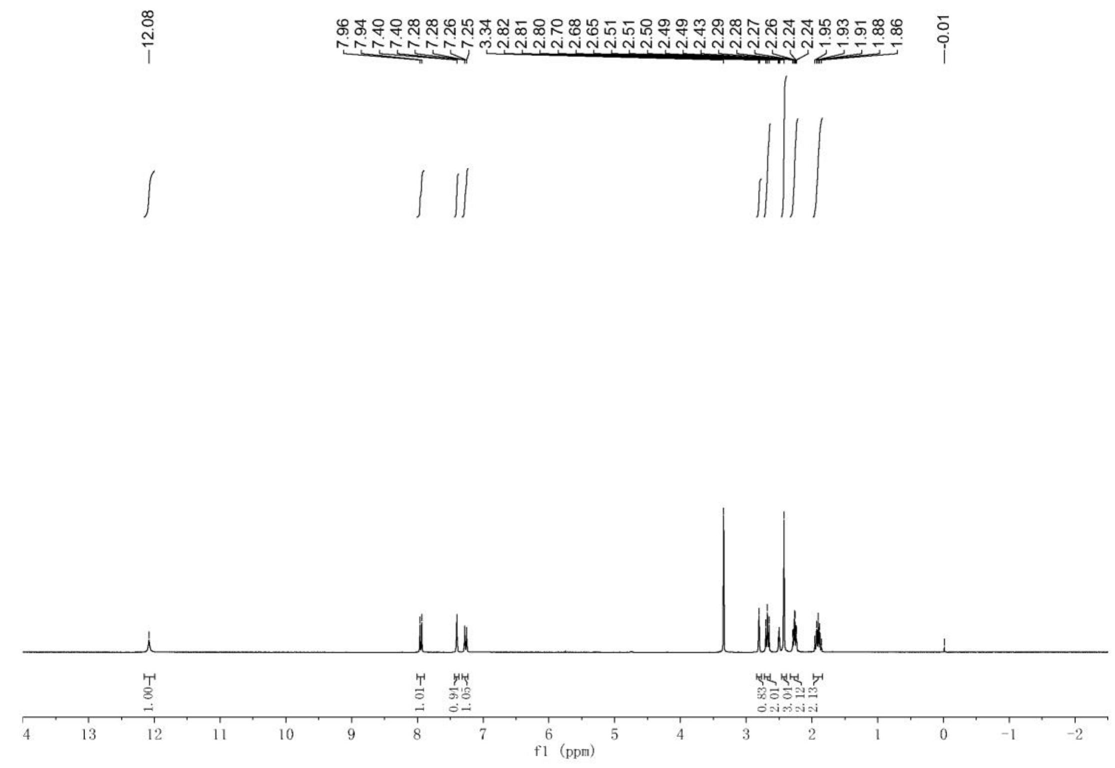

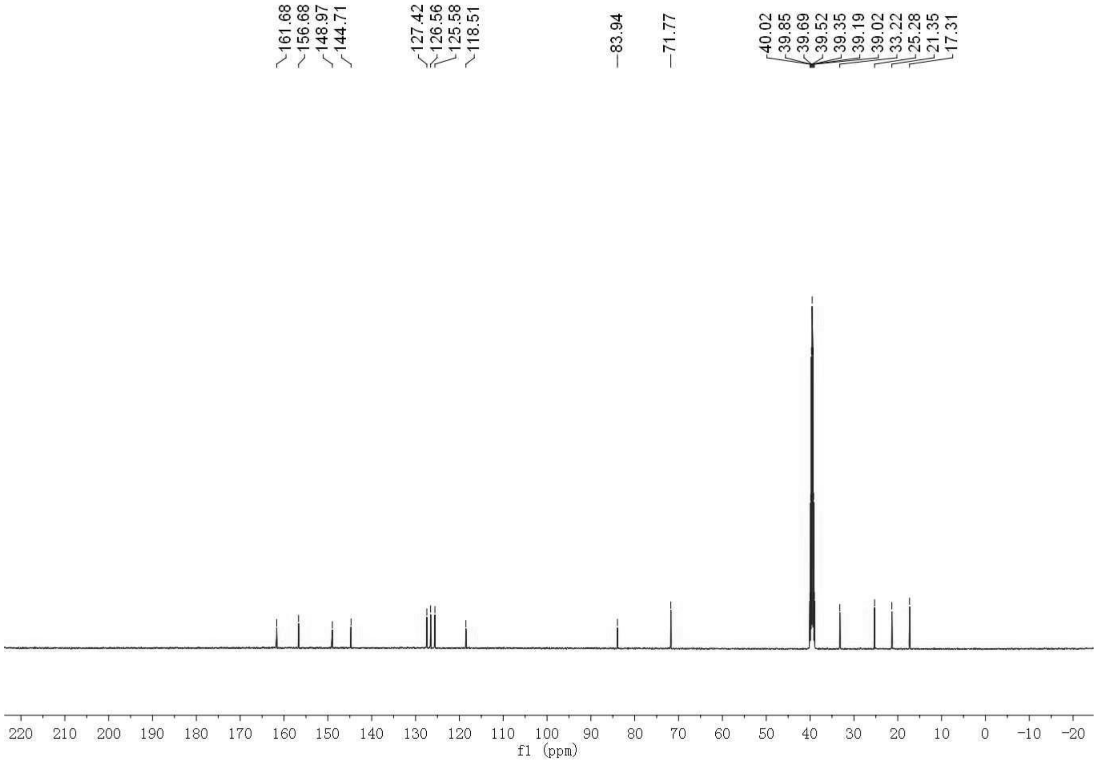

6,7,8,9-Tetrahydro-3-methyl-9-methylene-11*H*-pyrido[2,1-*b*]quinazolin-11-one (7C)

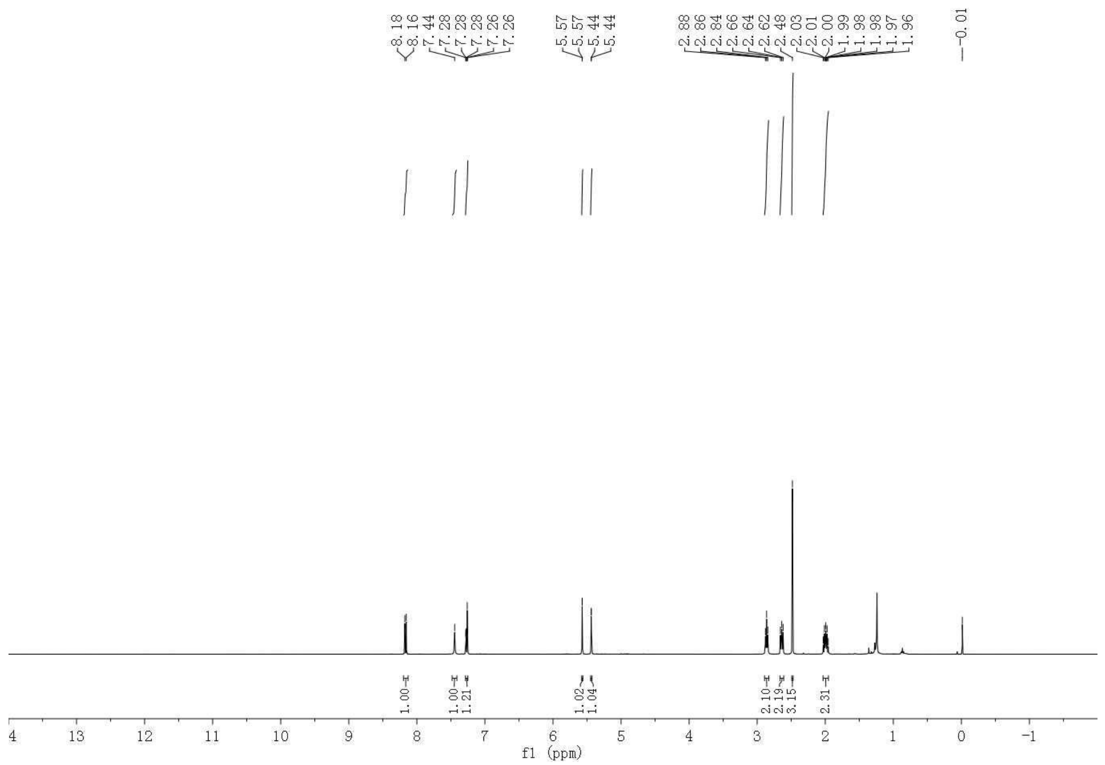

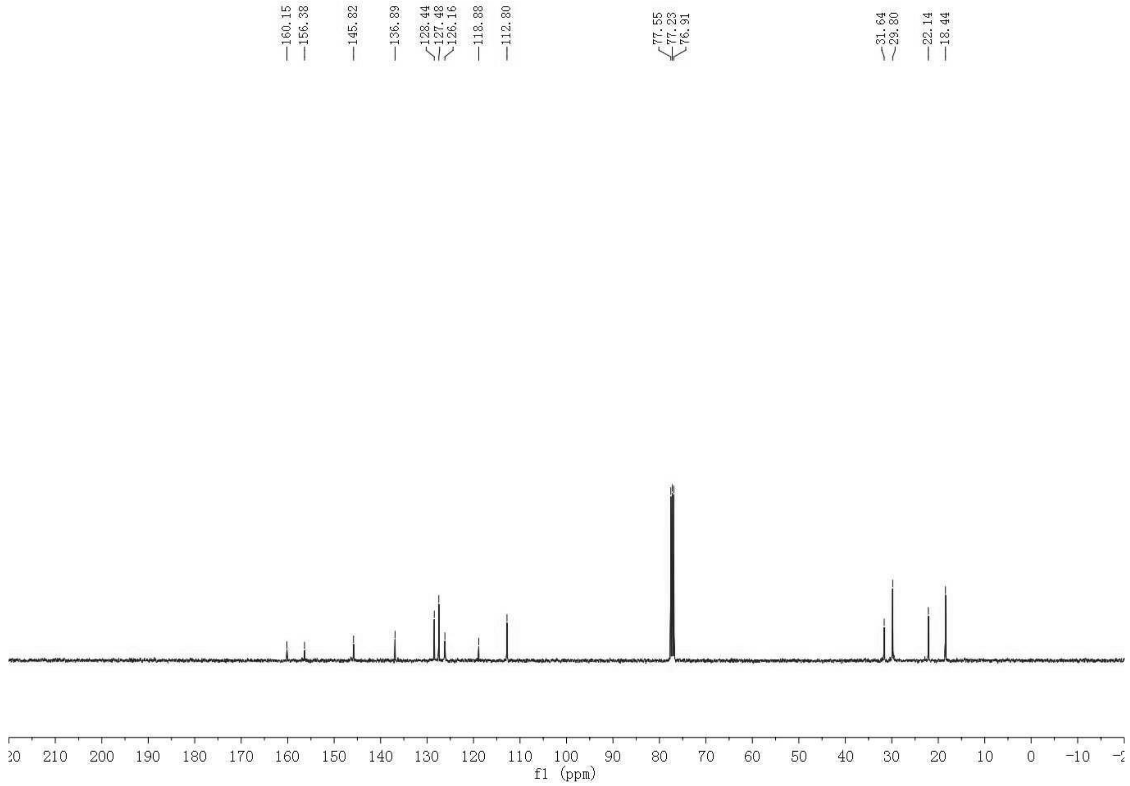

2-(4-Pentynyl)-6-methoxy-4(3*H*)-quinazolinone (6D)

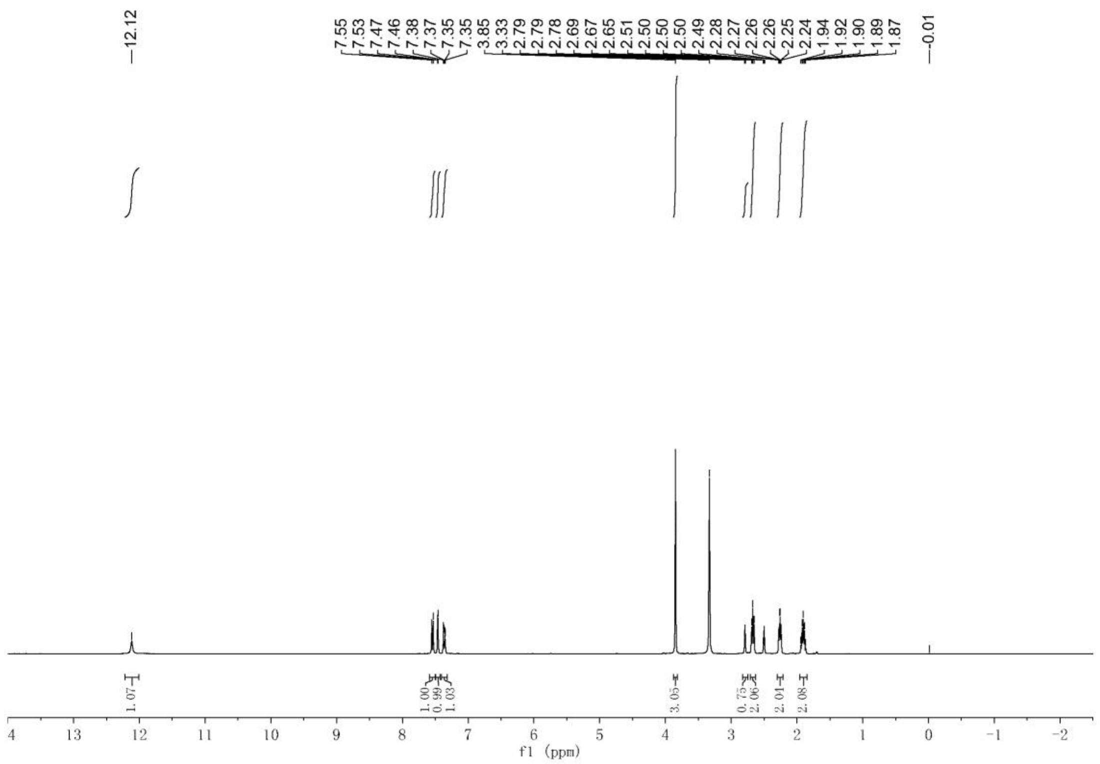

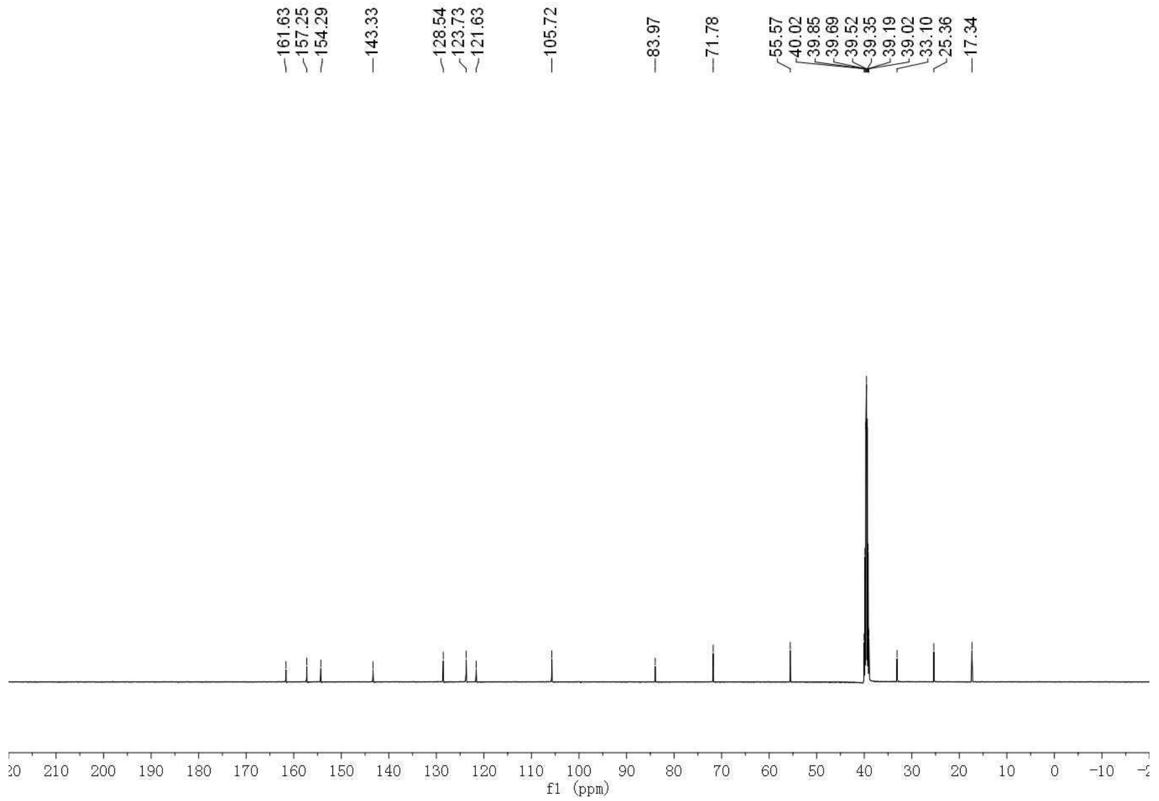

6,7,8,9-Tetrahydro-2-methoxy-9-methylene-11*H*-pyrido[2,1-*b*]quinazolin-11-one (7D)

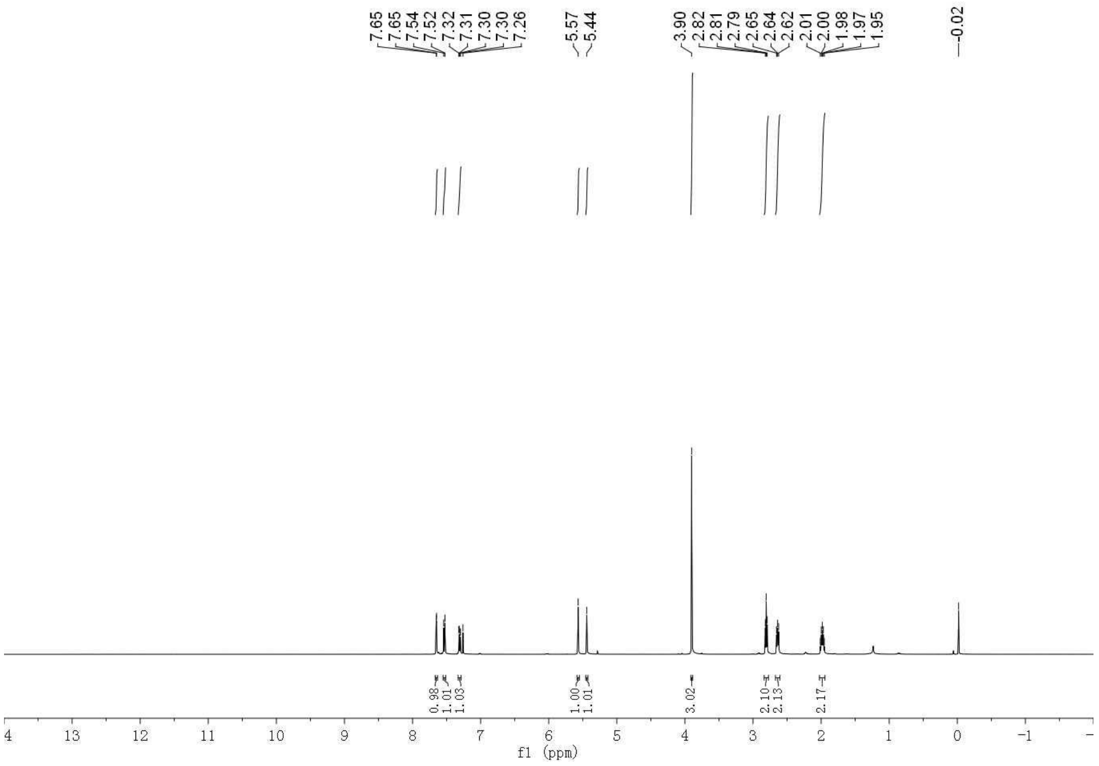

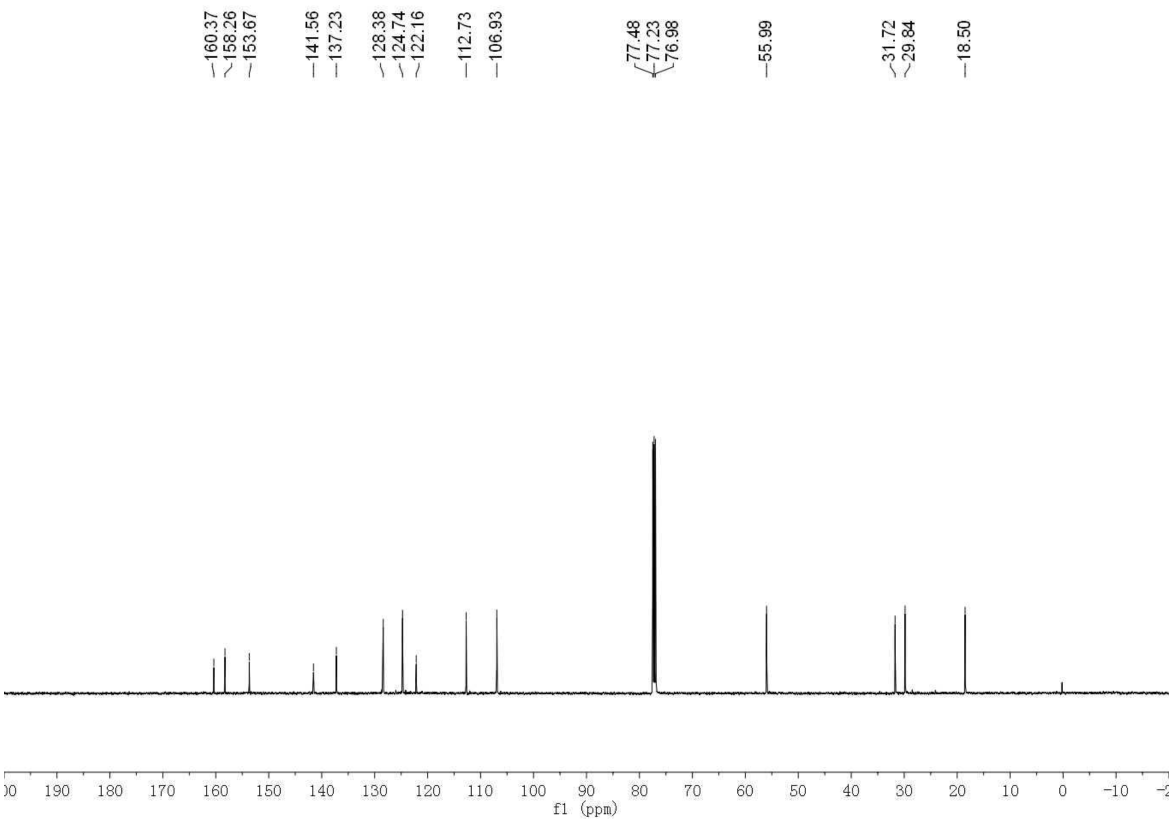

2-(4-Pentynyl)-5-fluoro-4(3*H*)-quinazolinone (6E)

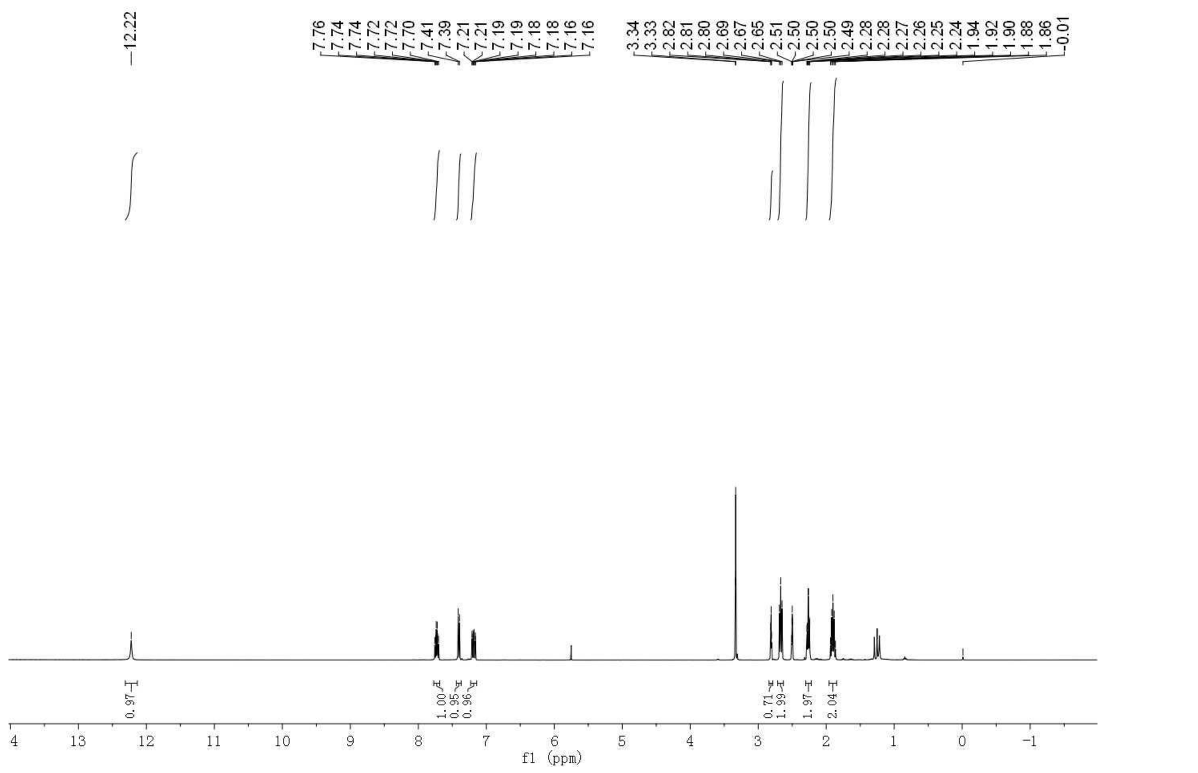

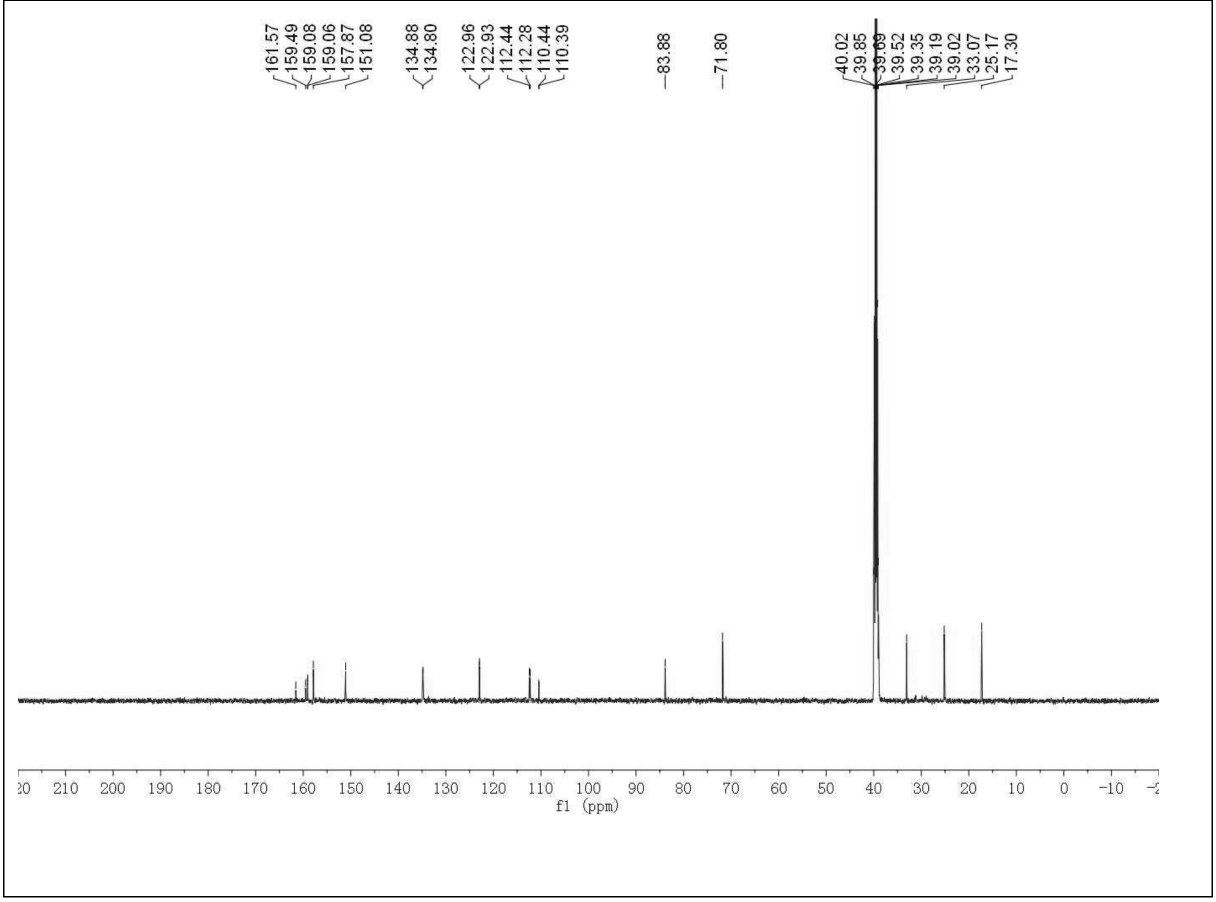

6,7,8,9-Tetrahydro-1-fluoro-9-methylene-11*H*-pyrido[2,1-*b*]quinazolin-11-one (7E)

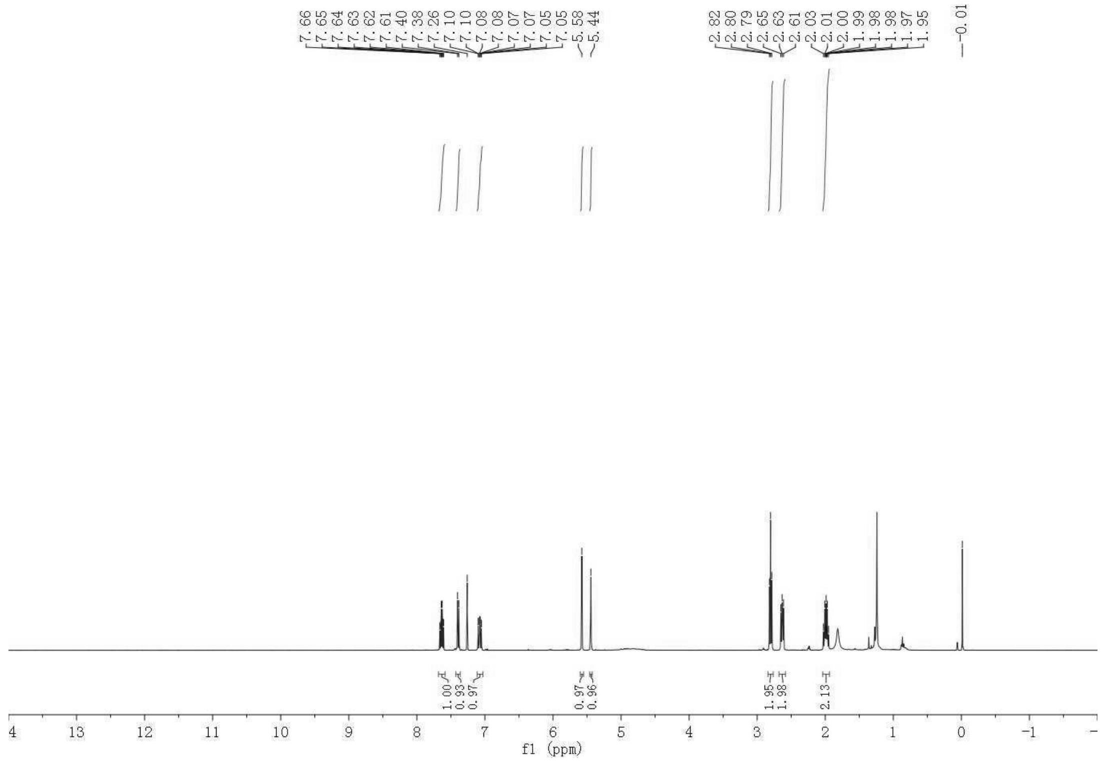

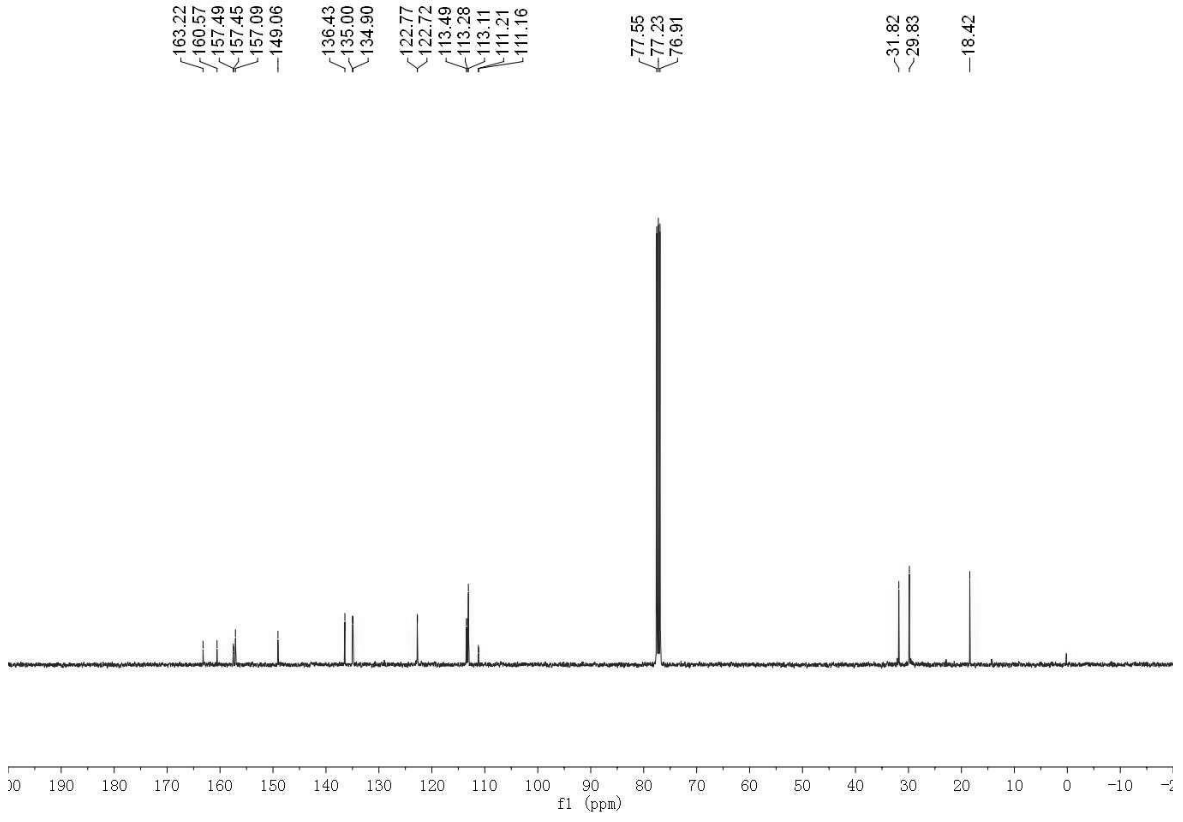

2-(4-Pentynyl)-5-chloro-4(3*H*)-quinazolinone (6F)

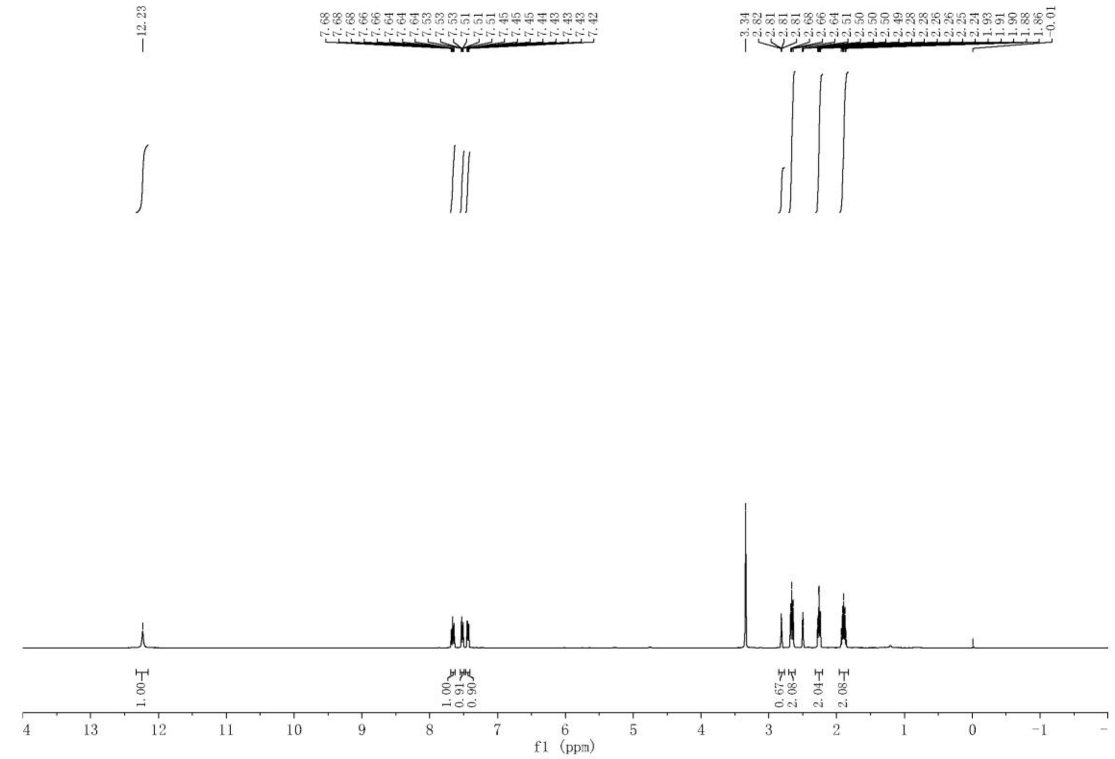

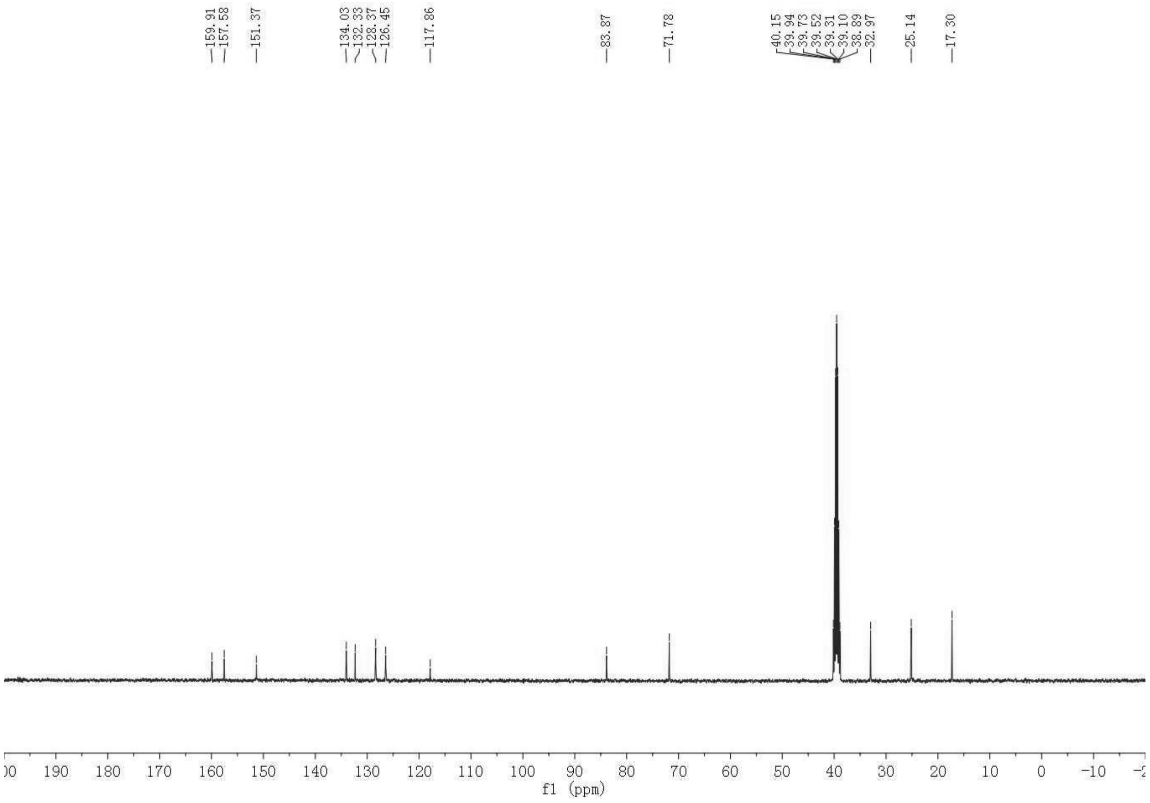

6,7,8,9-Tetrahydro-1-chloro-9-methylene-11*H*-pyrido[2,1-*b*]quinazolin-11-one (7F)

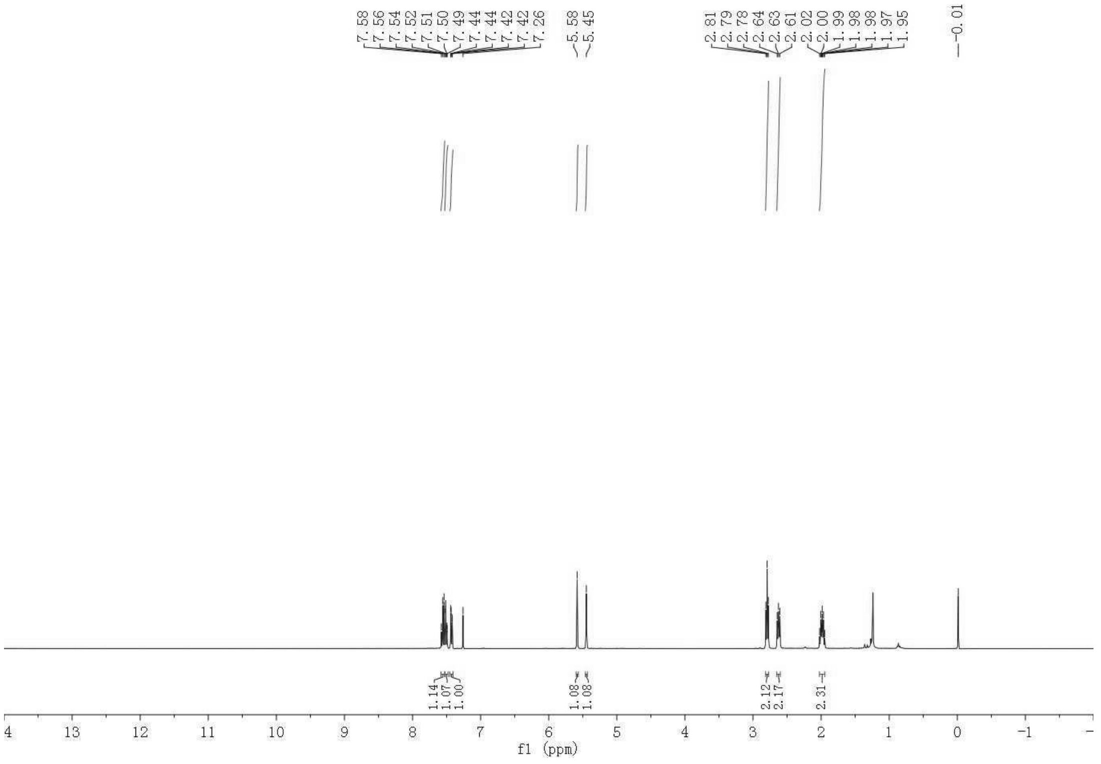

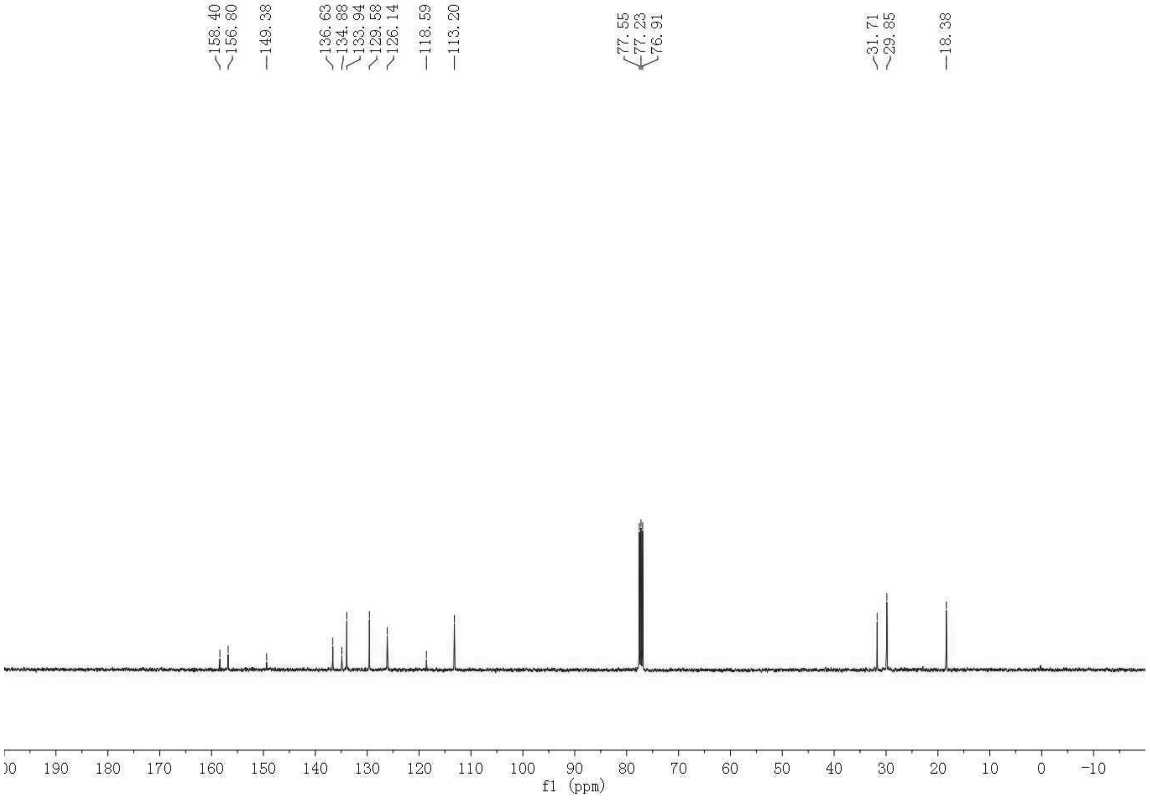

2-(4-Pentynyl)-6-chloro-4(3*H*)-quinazolinone (6G)

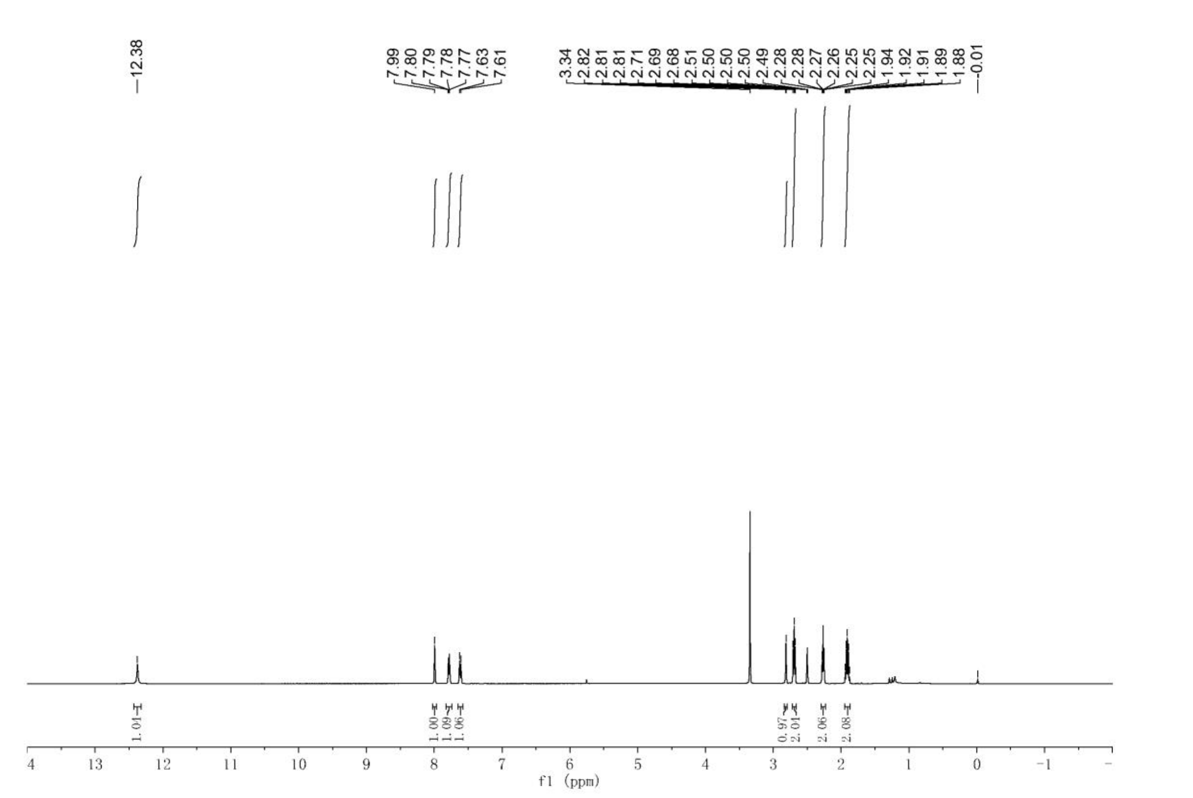

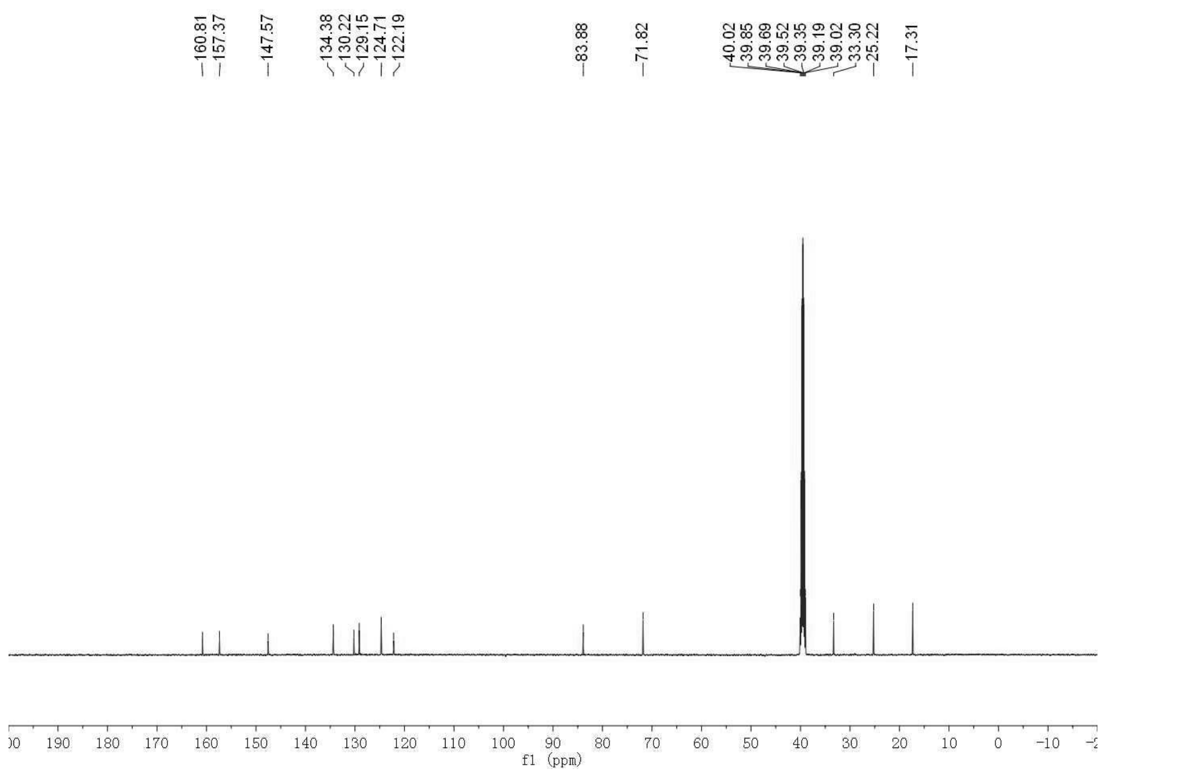

6,7,8,9-Tetrahydro-2-chloro-9-methylene-11*H*-pyrido[2,1-*b*]quinazolin-11-one (7G)

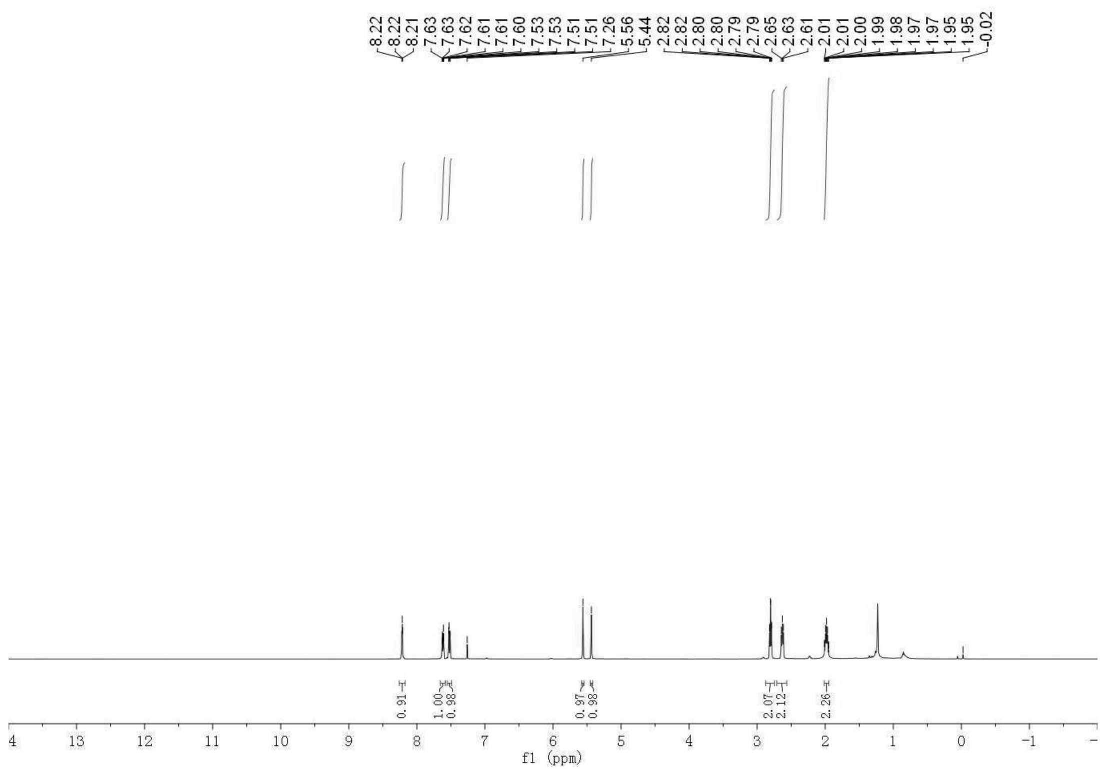

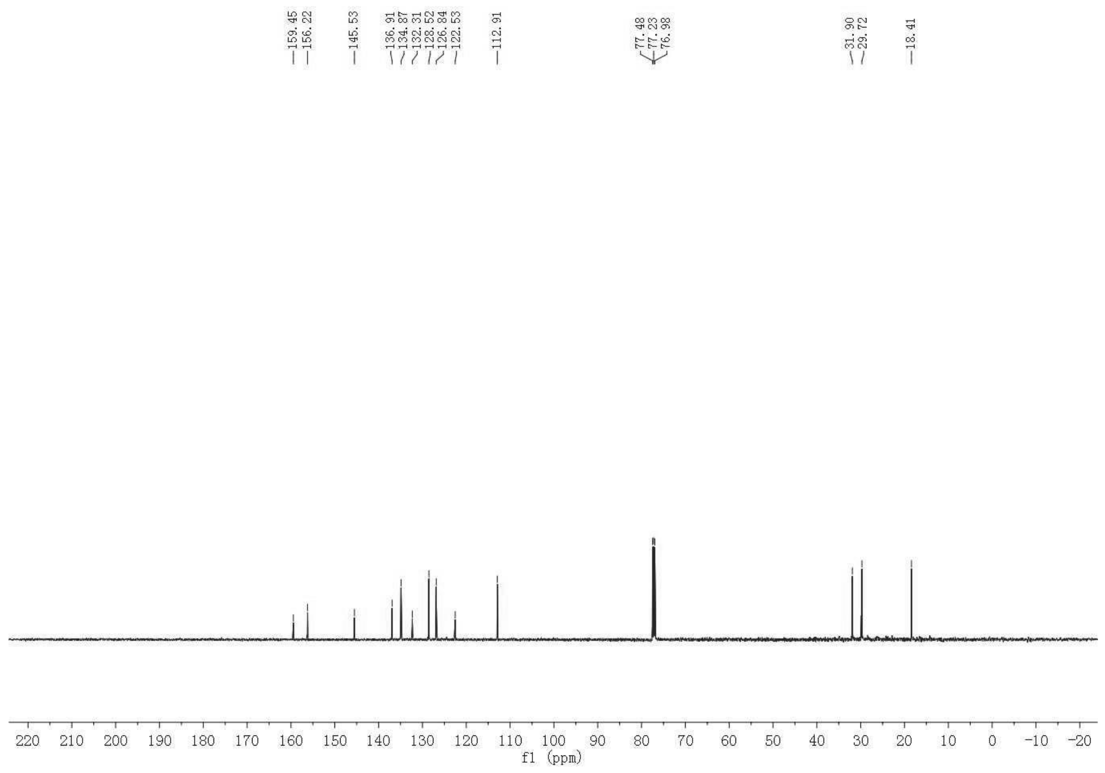

2-(4-Pentynyl)-7-chloro-4(3*H*)-quinazolinone (6H)

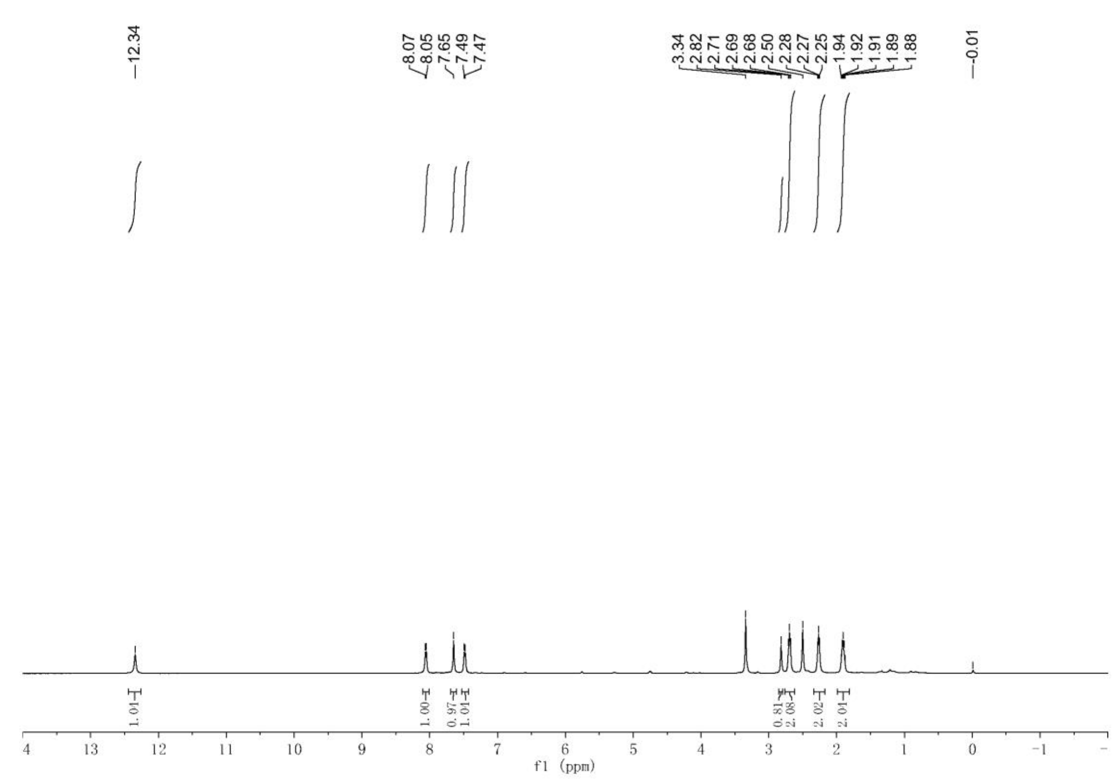

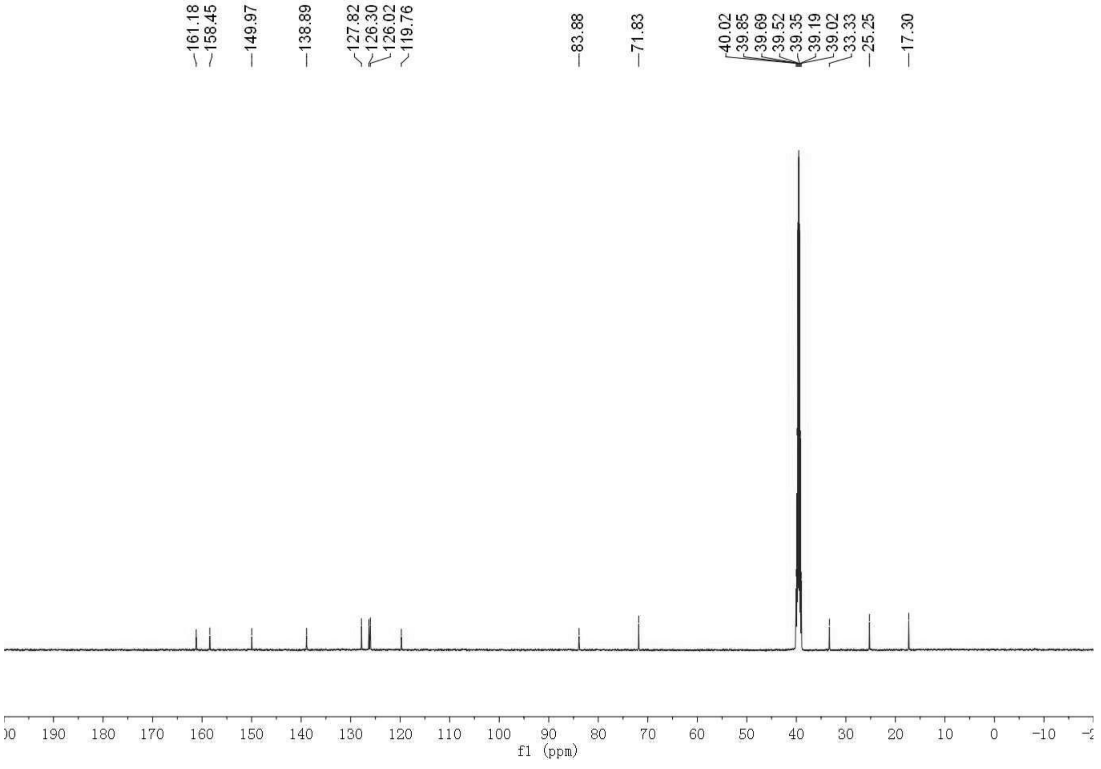

6,7,8,9-Tetrahydro-3-chloro-9-methylene-11*H*-pyrido[2,1-*b*]quinazolin-11-one (7H)

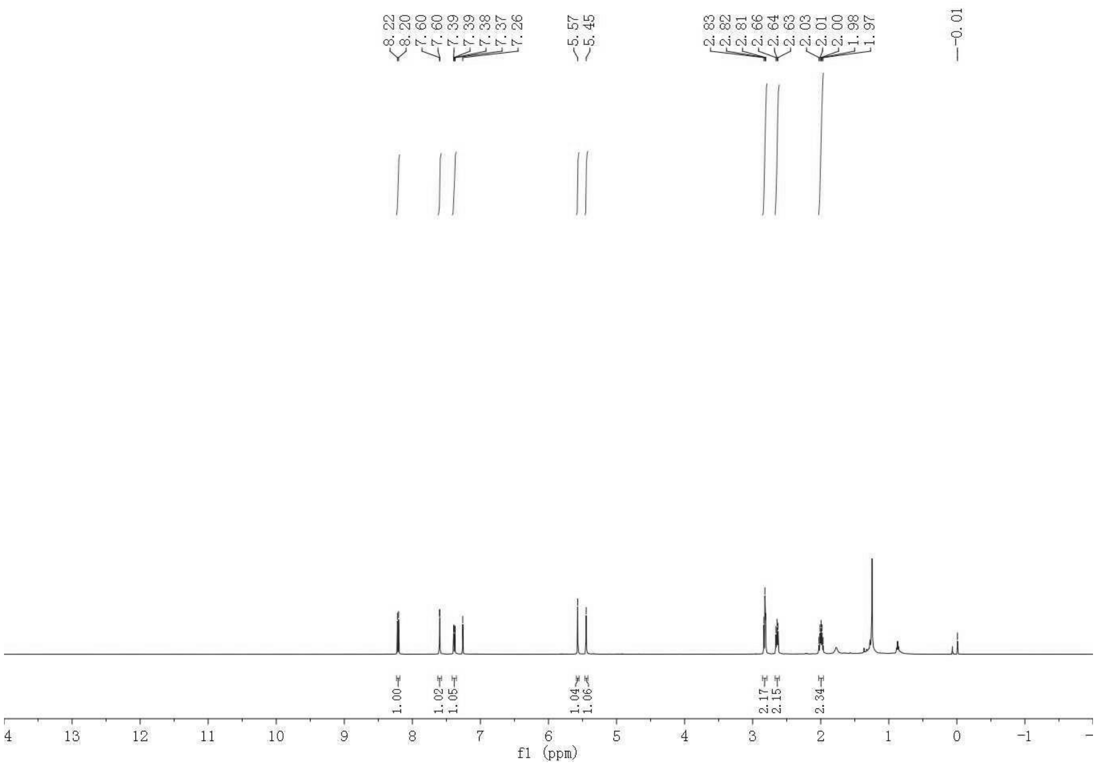

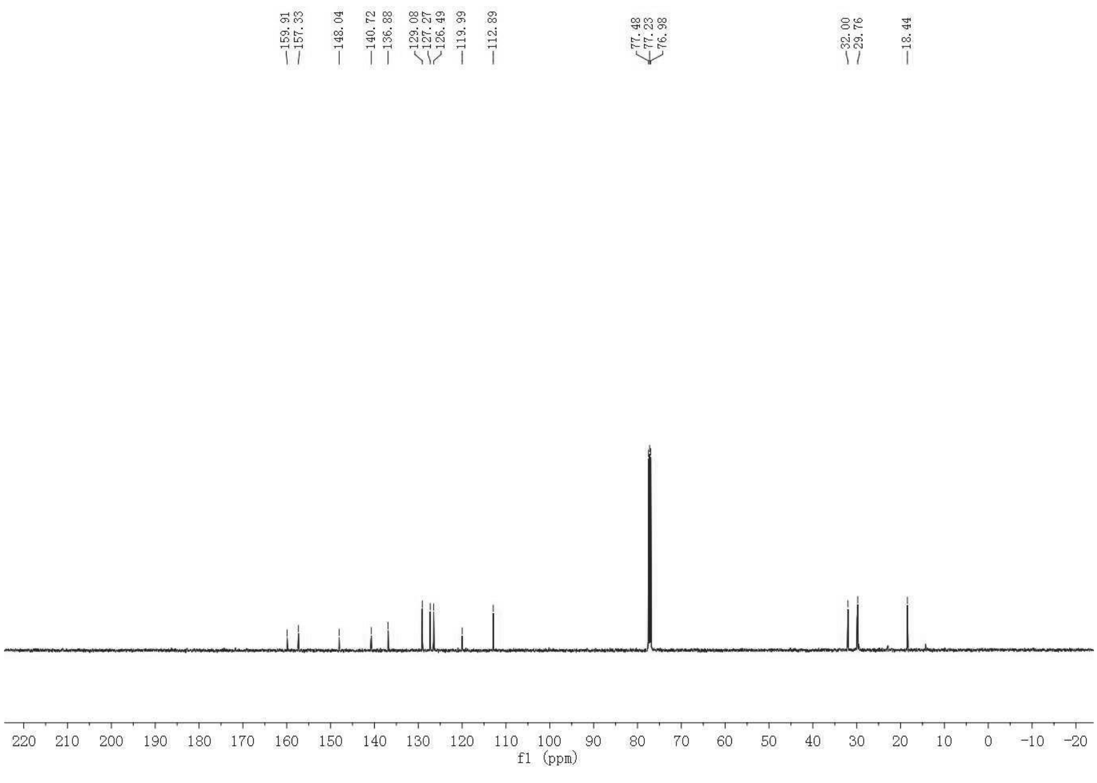

2-(4-Pentynyl)-6-bromo-4(3*H*)-quinazolinone (6l)

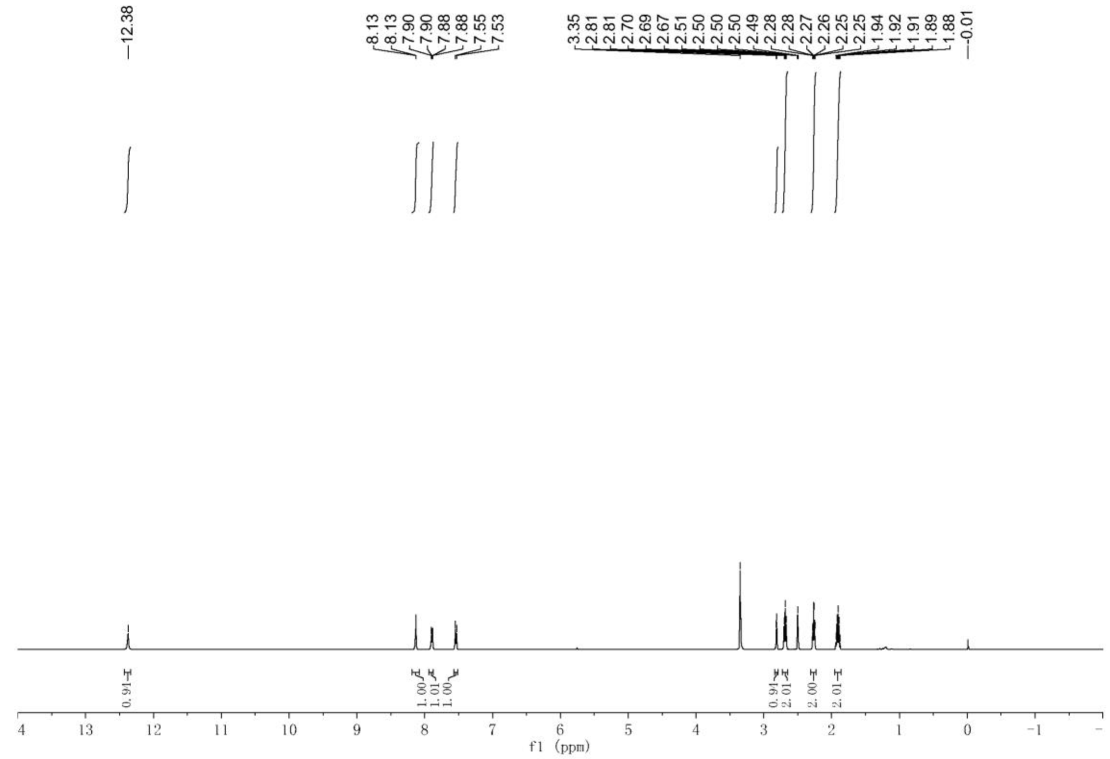

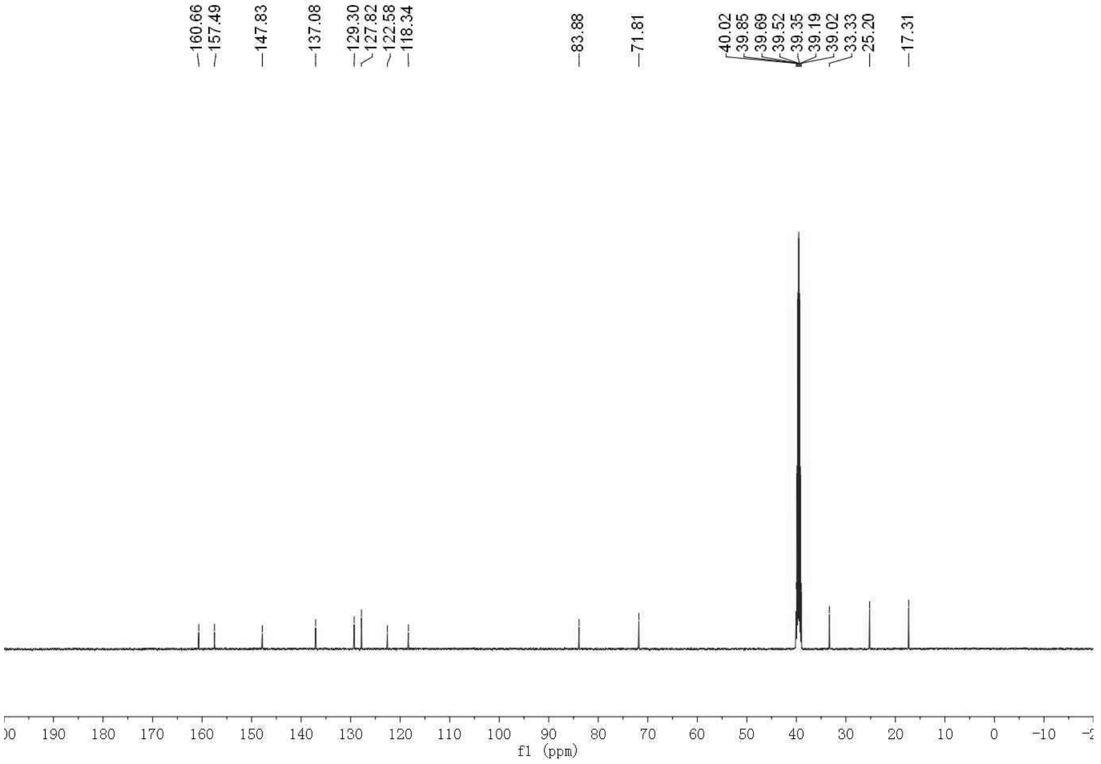

6,7,8,9-Tetrahydro-2-bromo-9-methylene-11*H*-pyrido[2,1-*b*]quinazolin-11-one (7l)

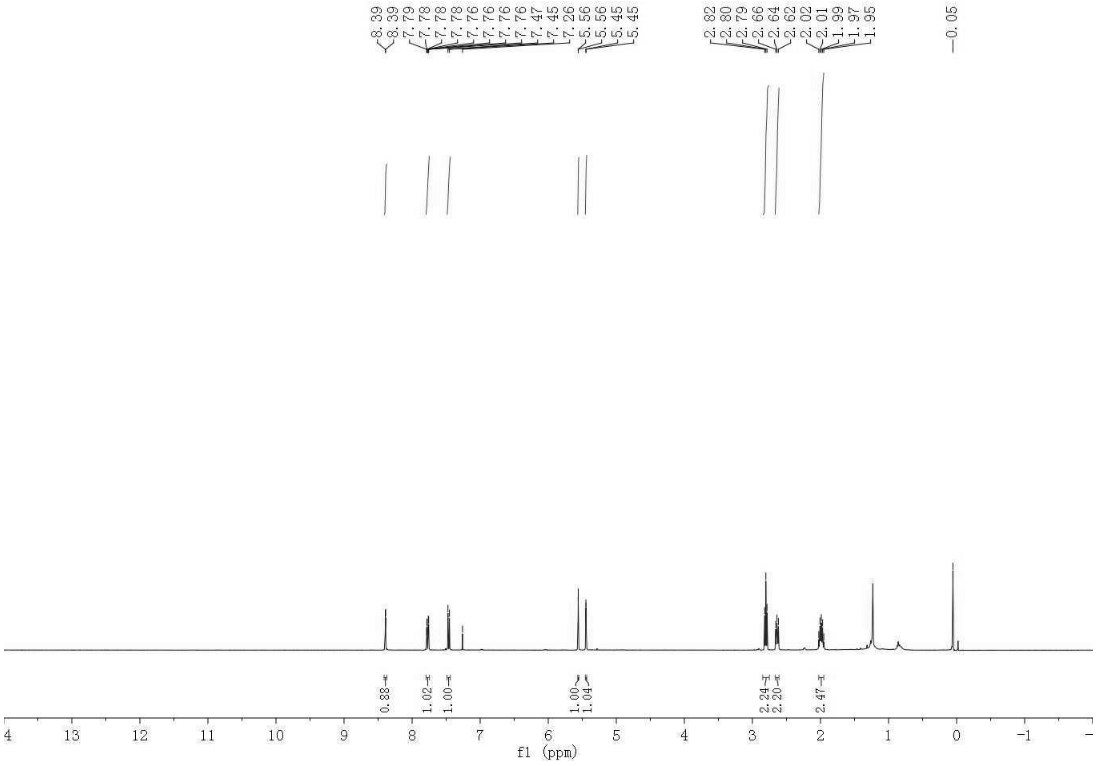

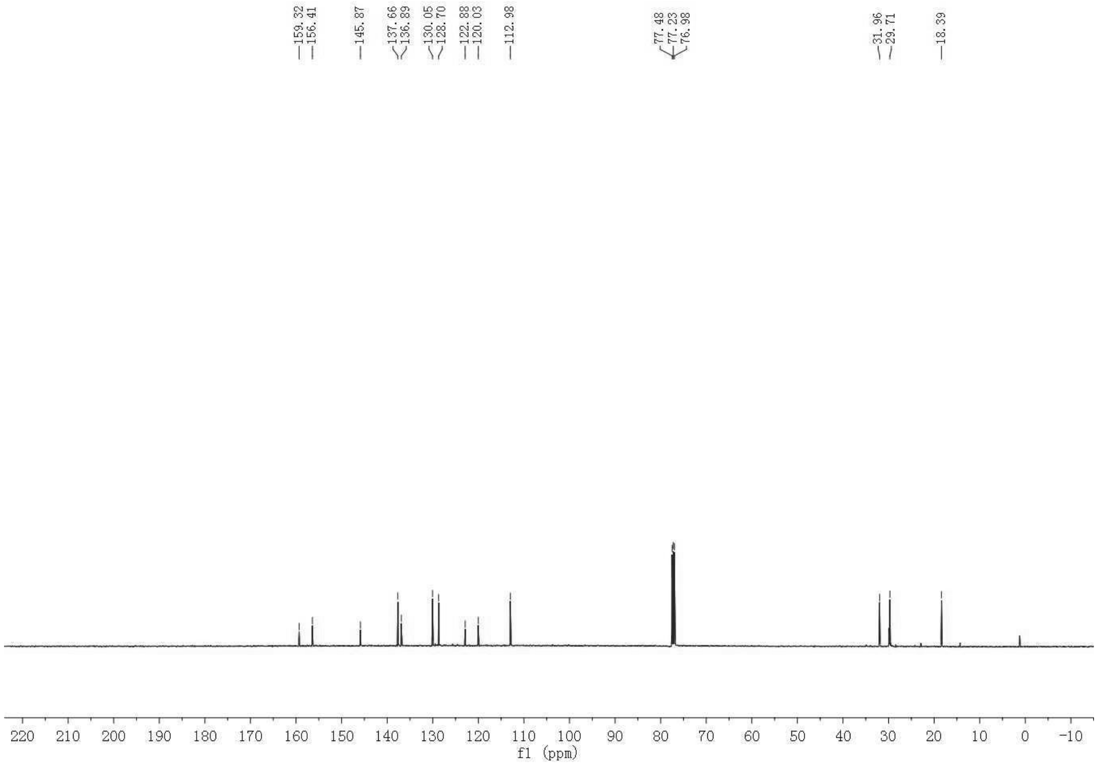

2-(4-Pentynyl)-7-phenyl-4(3*H*)-quinazolinone (6J)

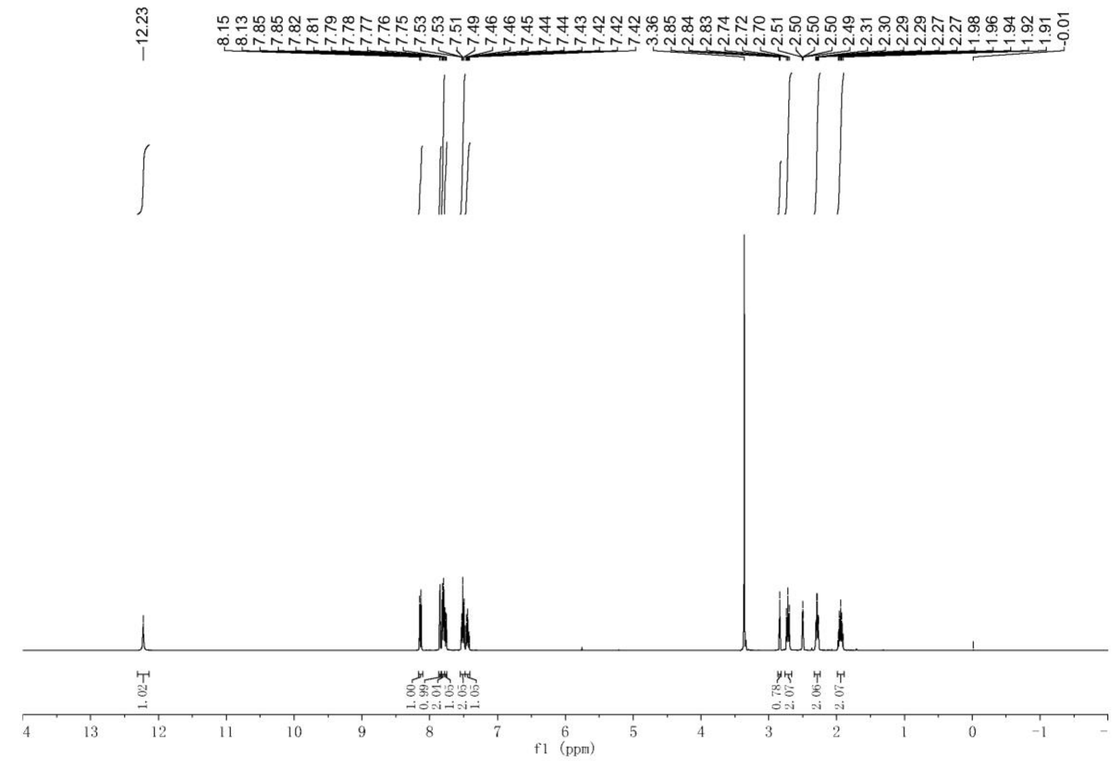

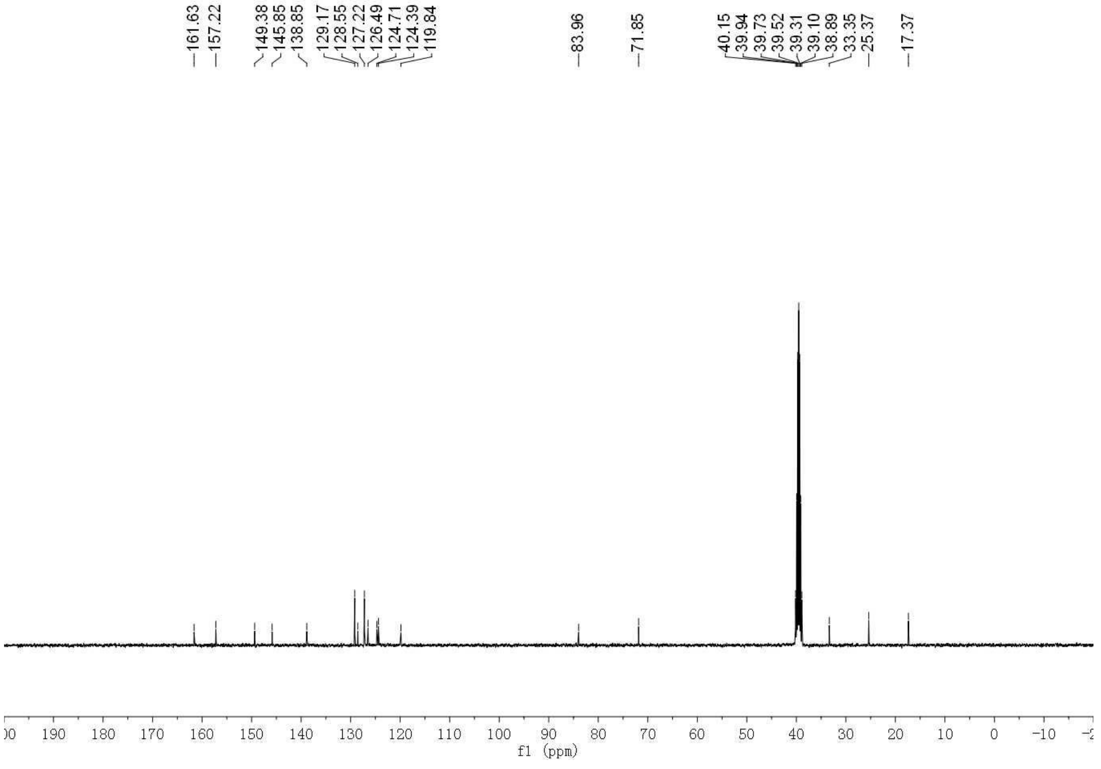

6,7,8,9-Tetrahydro-3-phenyl-9-methylene-11*H*-pyrido[2,1-*b*]quinazolin-11-one (7J)

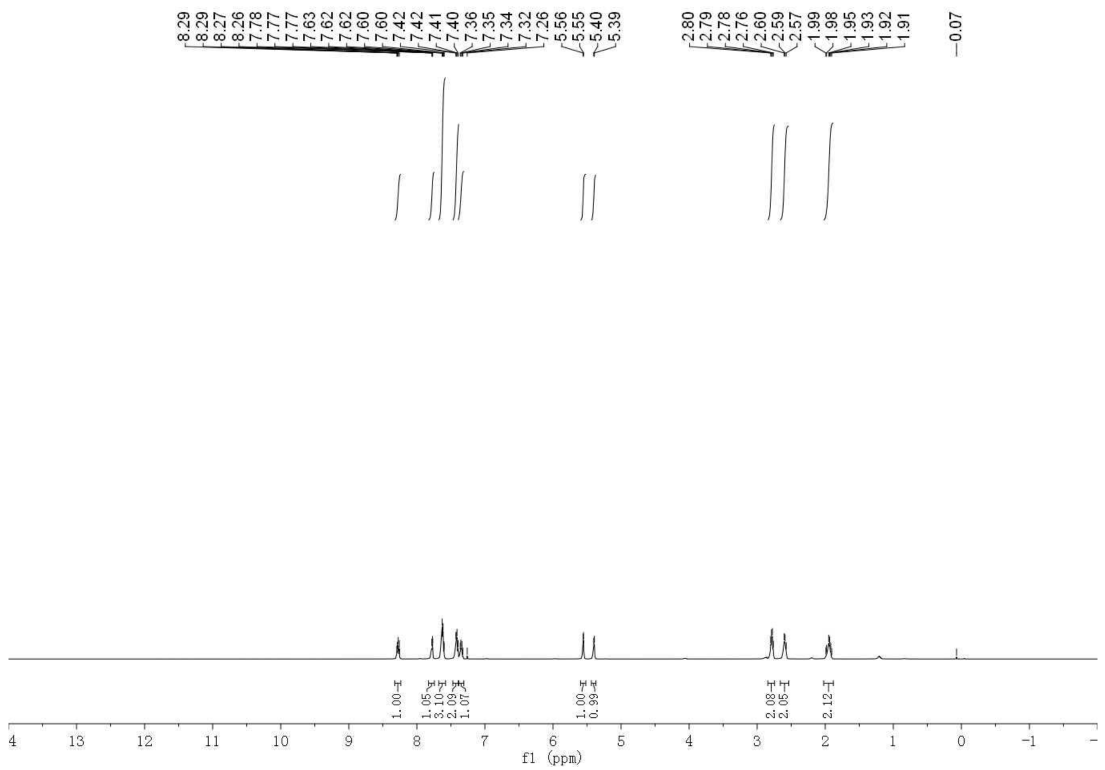

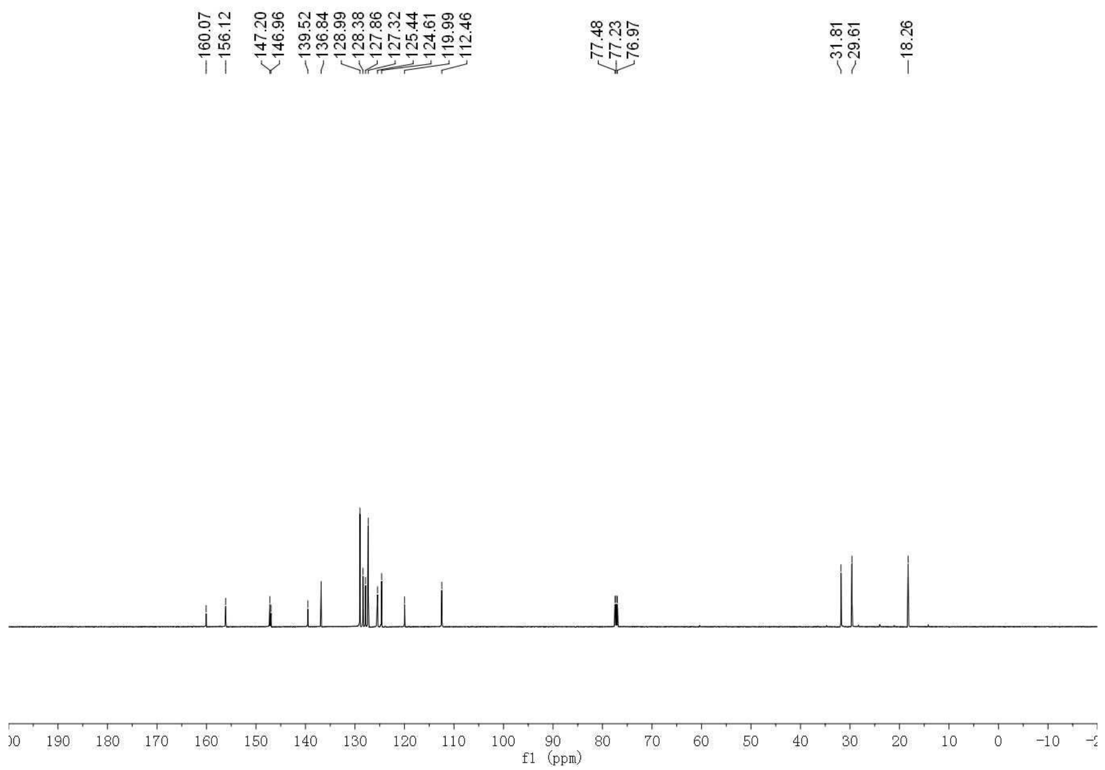

2-(4-Pentynyl)-4(3*H*)-benzo[*g*]quinazolinone (6K)

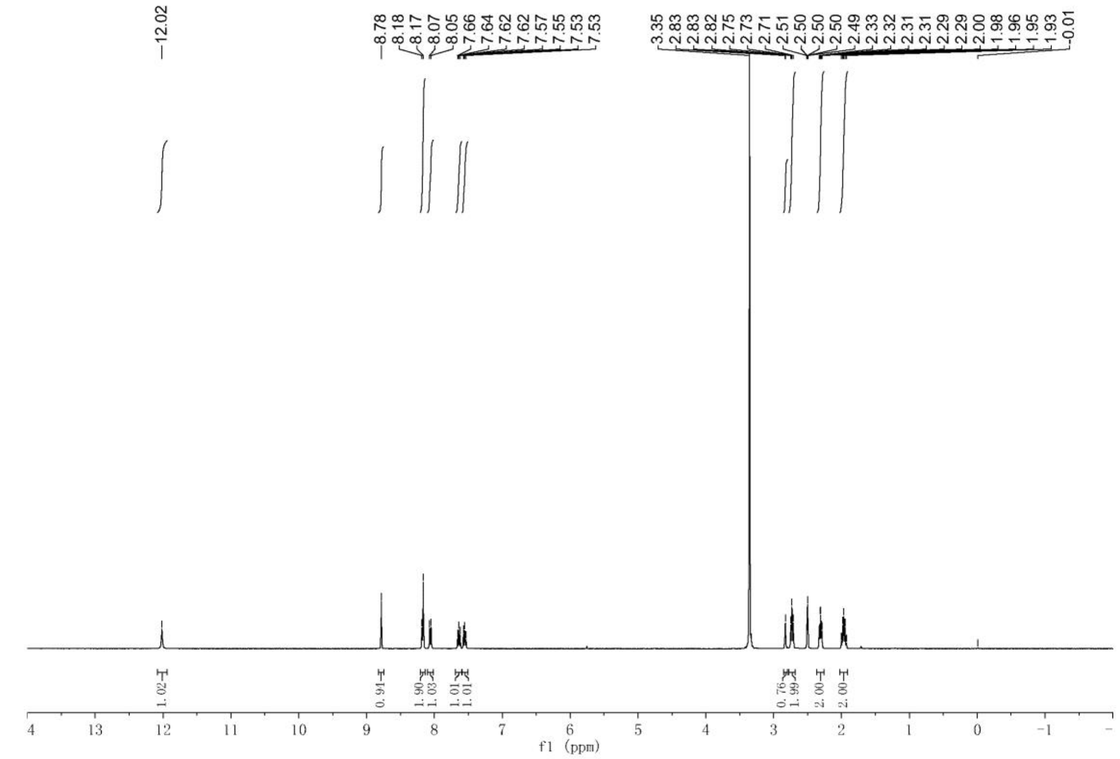

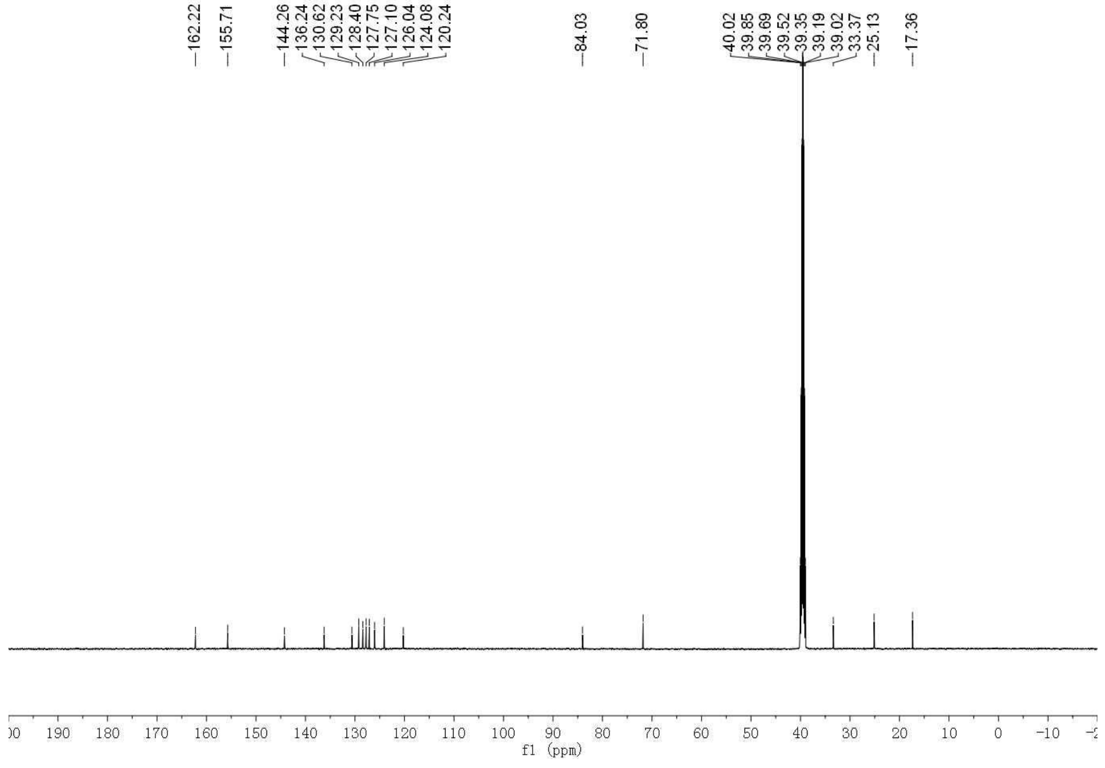

1,2,3,4-Tetrahydro-1-methylene-12*H*-benzo[*g*]pyrido[2,1-*b*]quinazolin-12-one (7K)

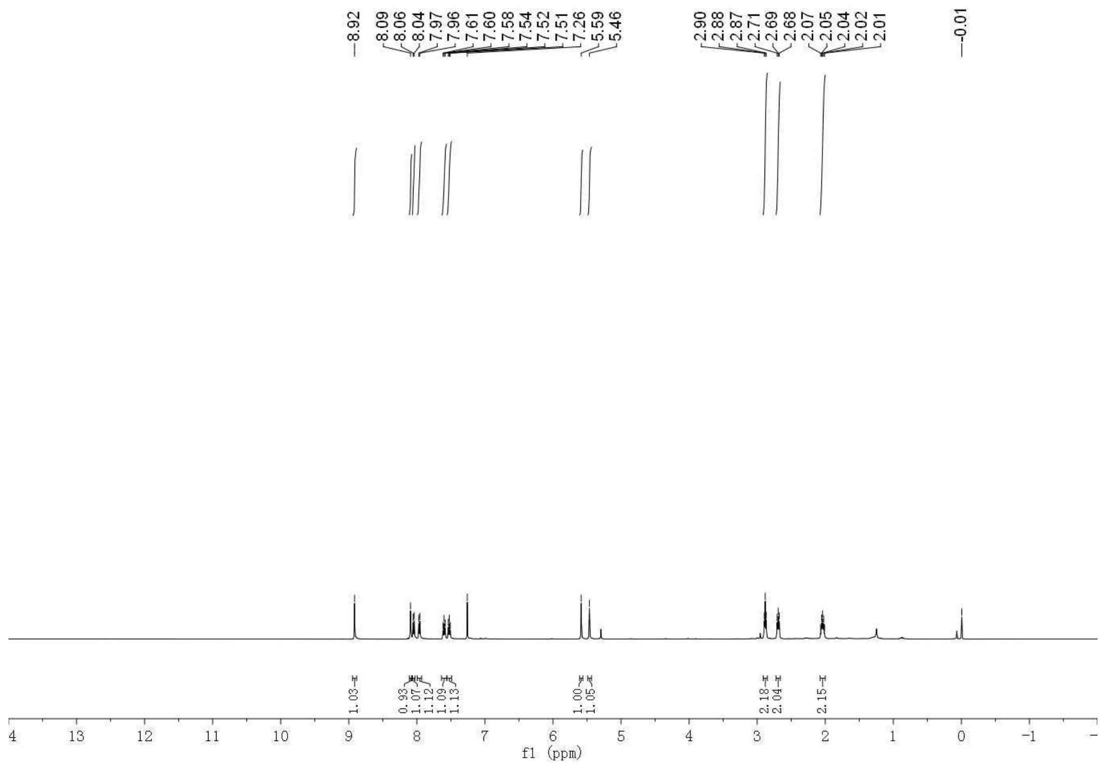

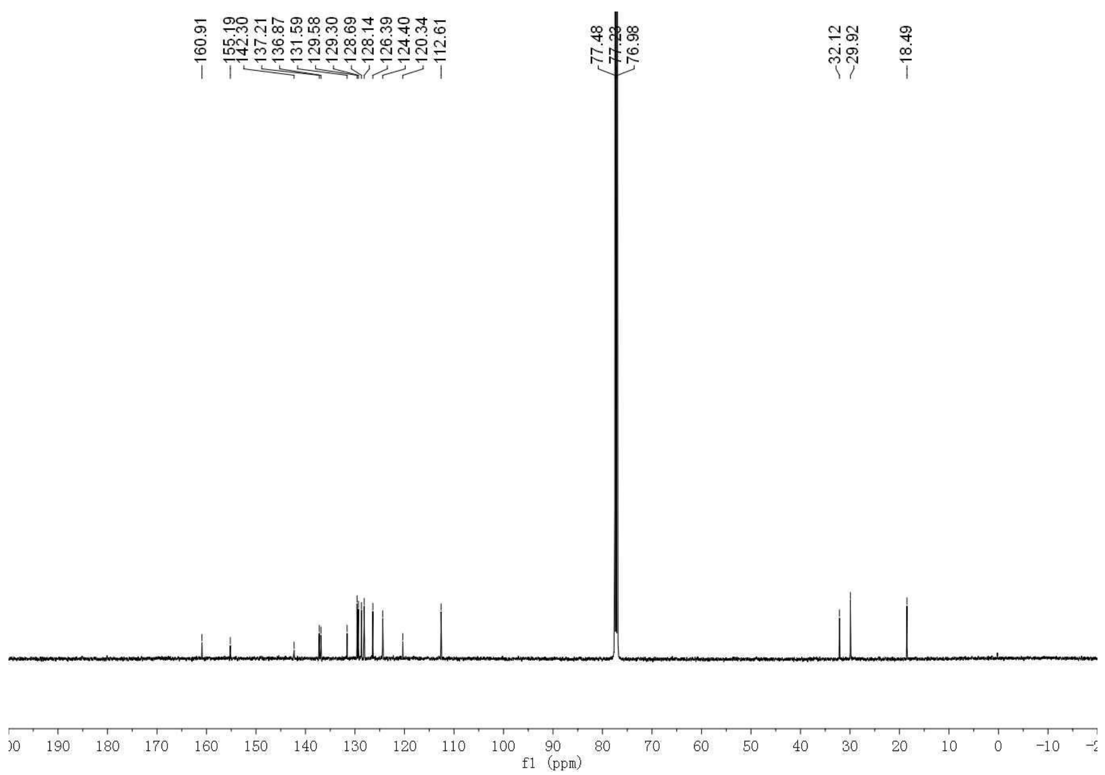

2-(4-Pentynyl)-thieno[2,3-*d*]pyrimidin-4(1*H*)-one (6L)

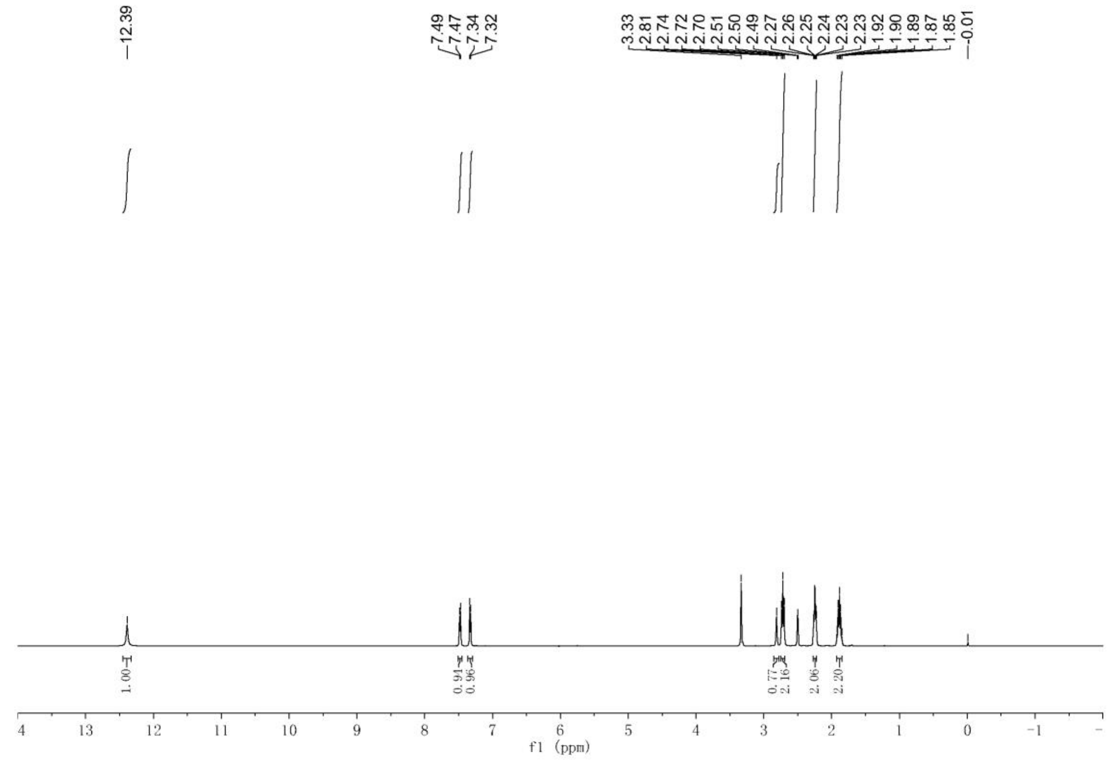

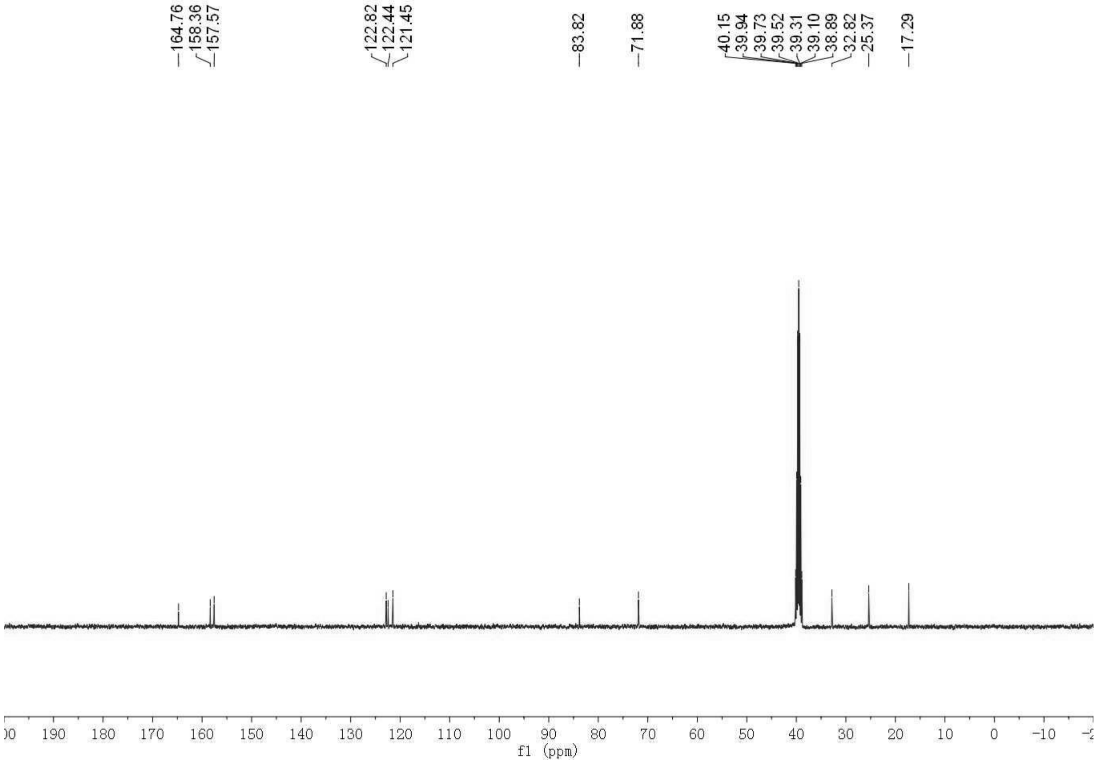

6,7,8,9-Tetrahydro-6-methylene-4*H*-pyrido[1,2-*a*]thieno[2,3-*d*]pyrimidin-4-one (7L)

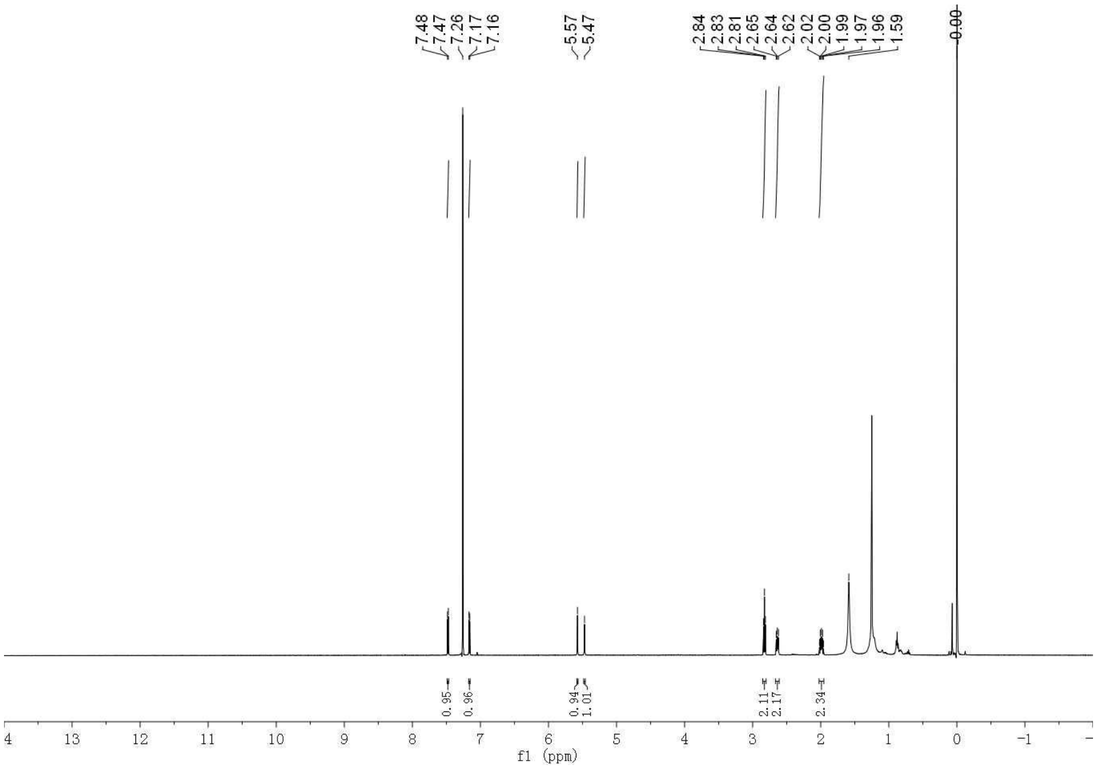

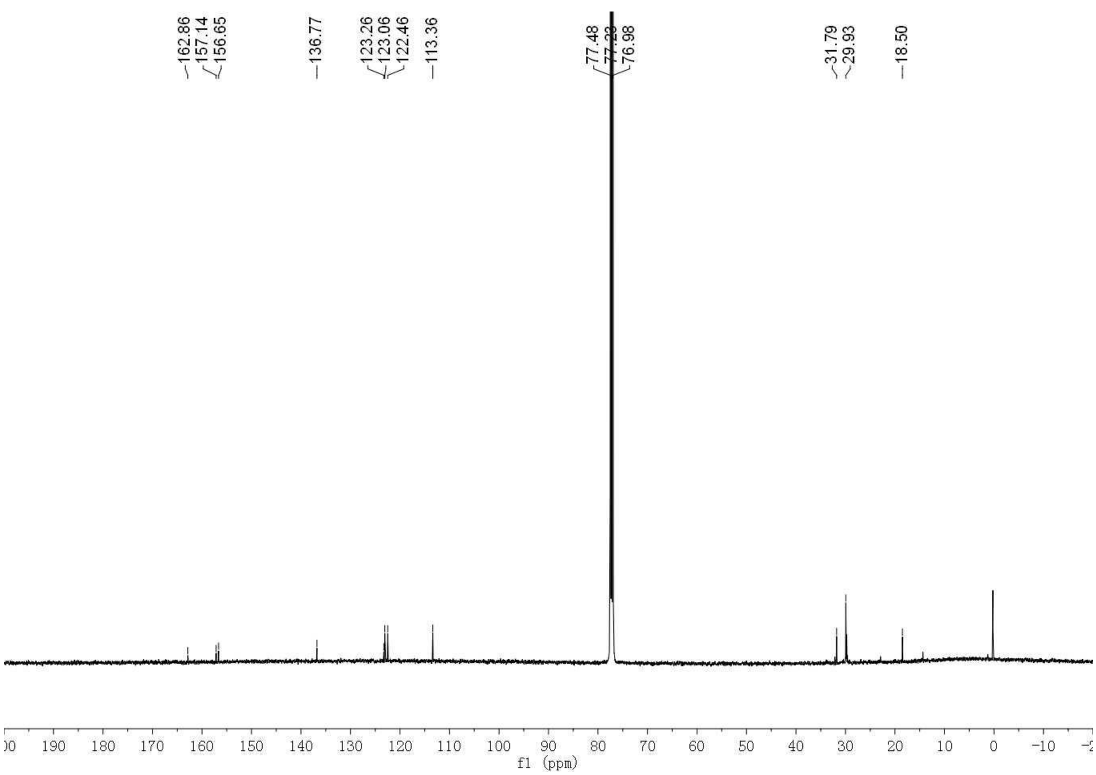

2-(3-Butynyl)-4(3*H*)-quinazolinone (8A)

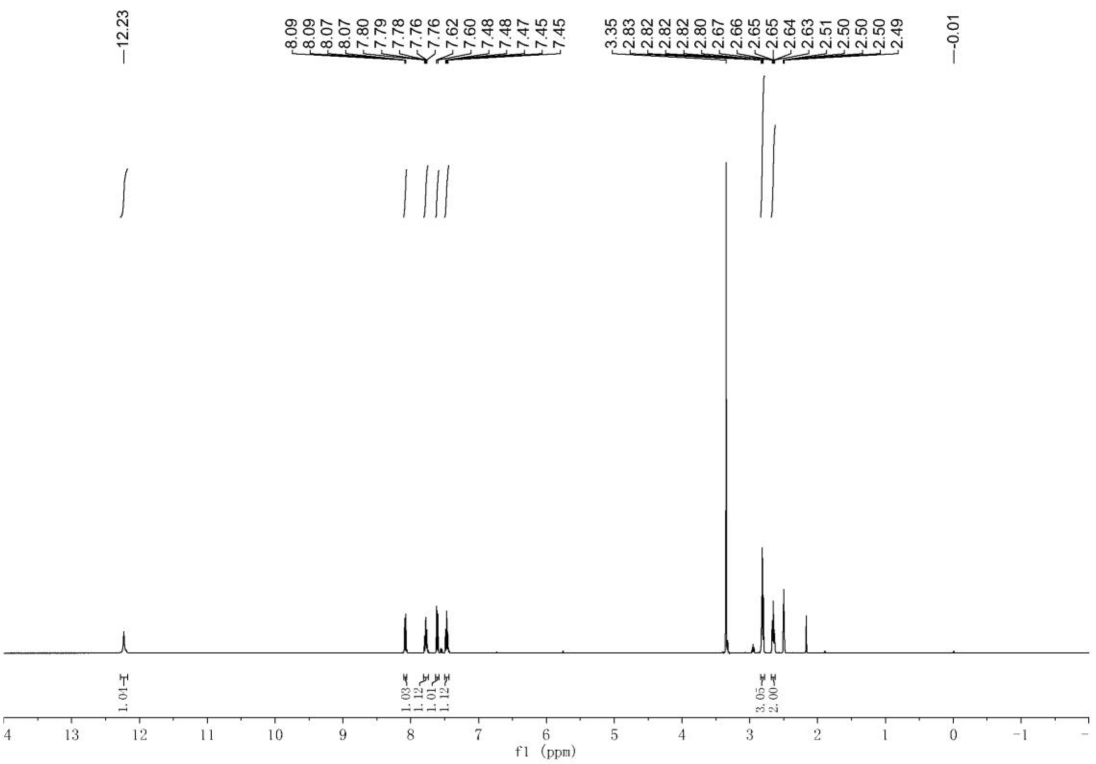

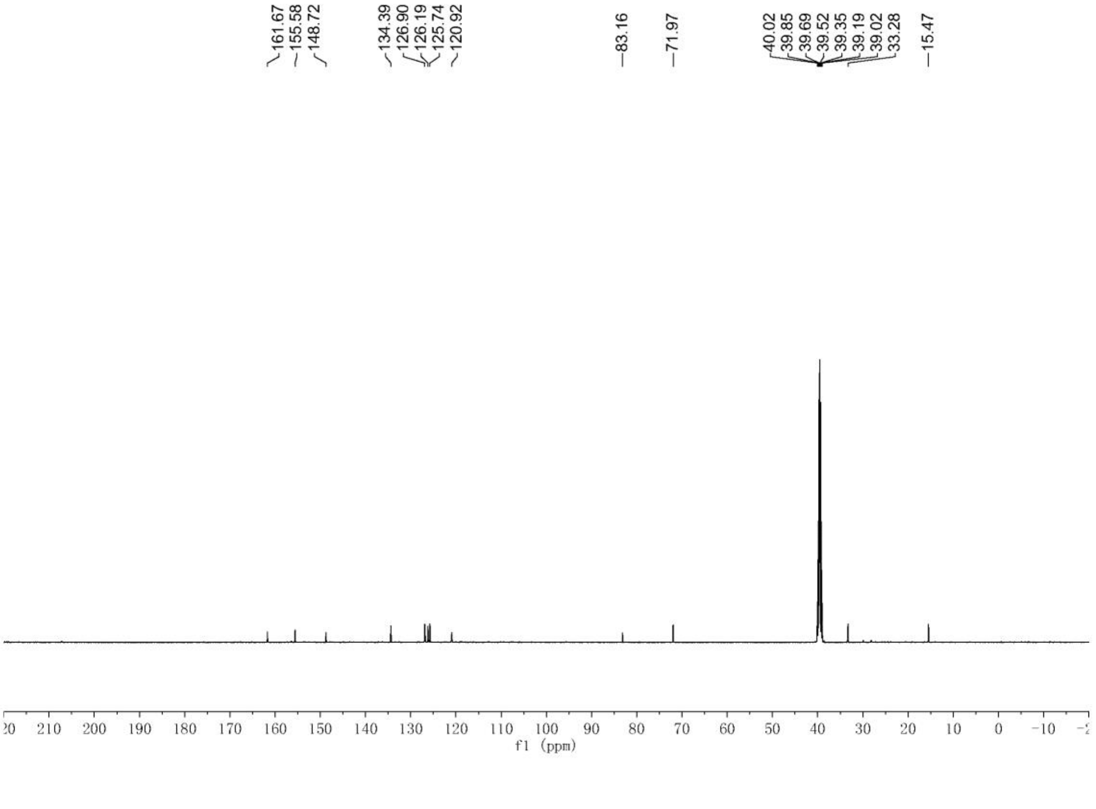

2,3-Dihydro-1-methylene-pyrrolo[2,1-*b*]quinazolin-9(1*H*)-one (9A)

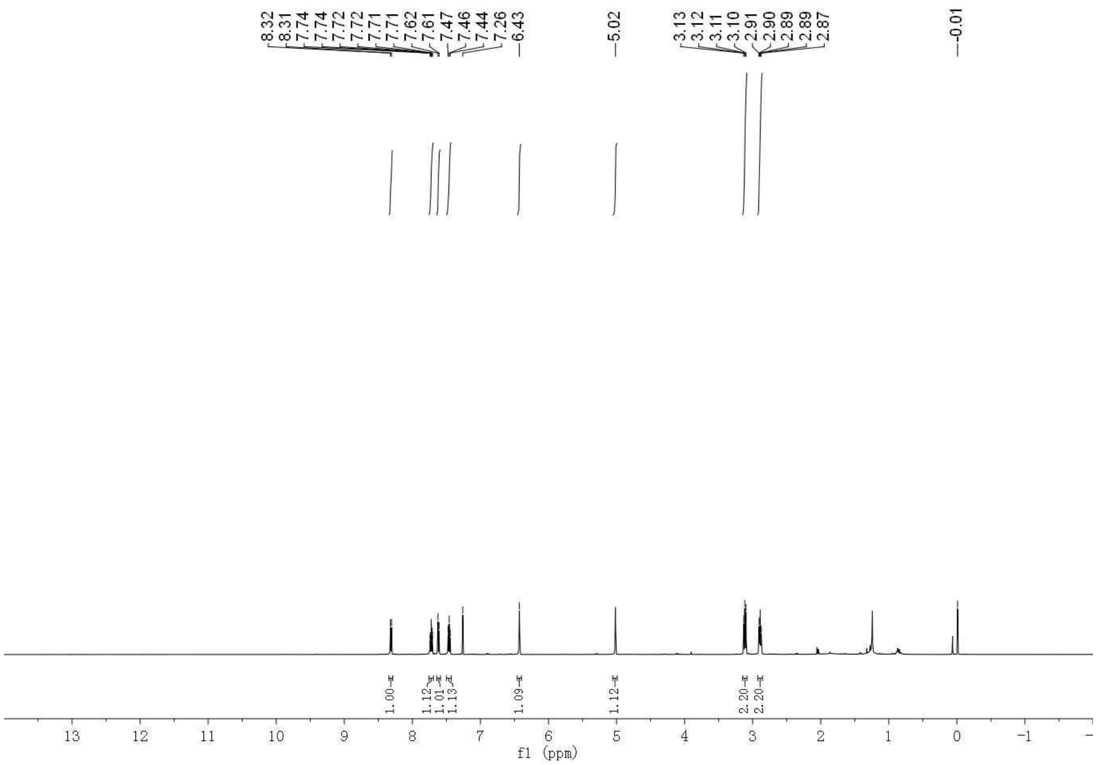

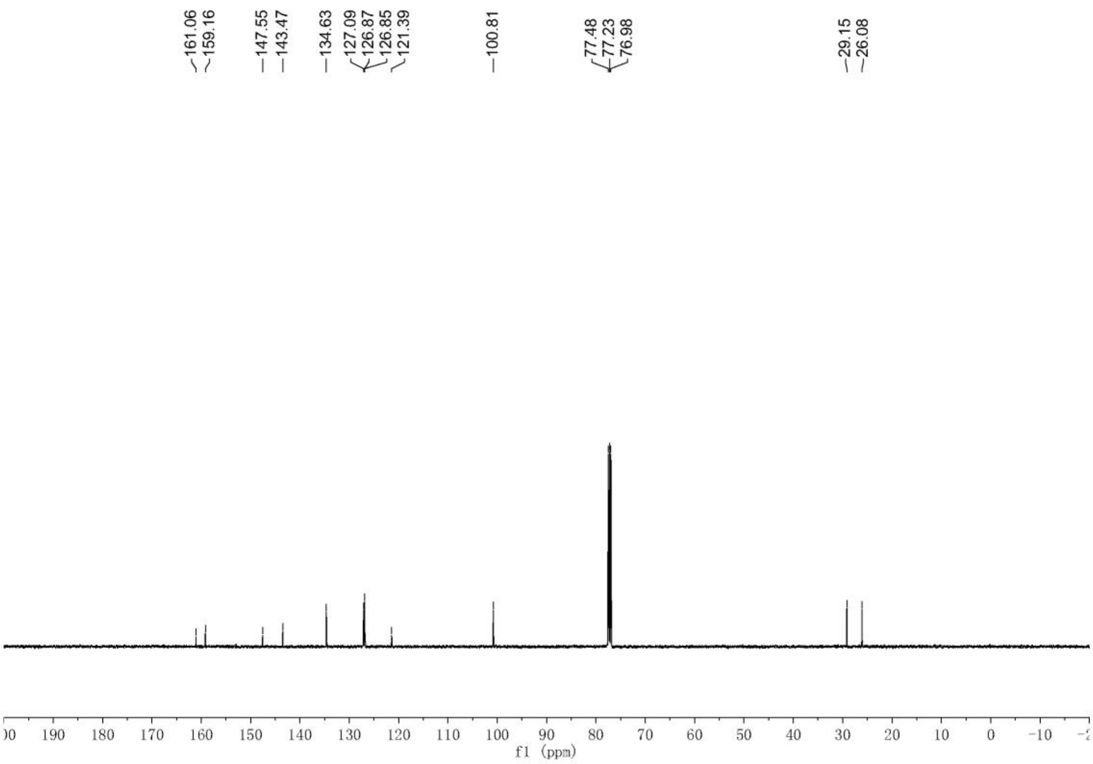

2-(3-Butynyl)-6-methyl-4(3*H*)-quinazolinone (8B)

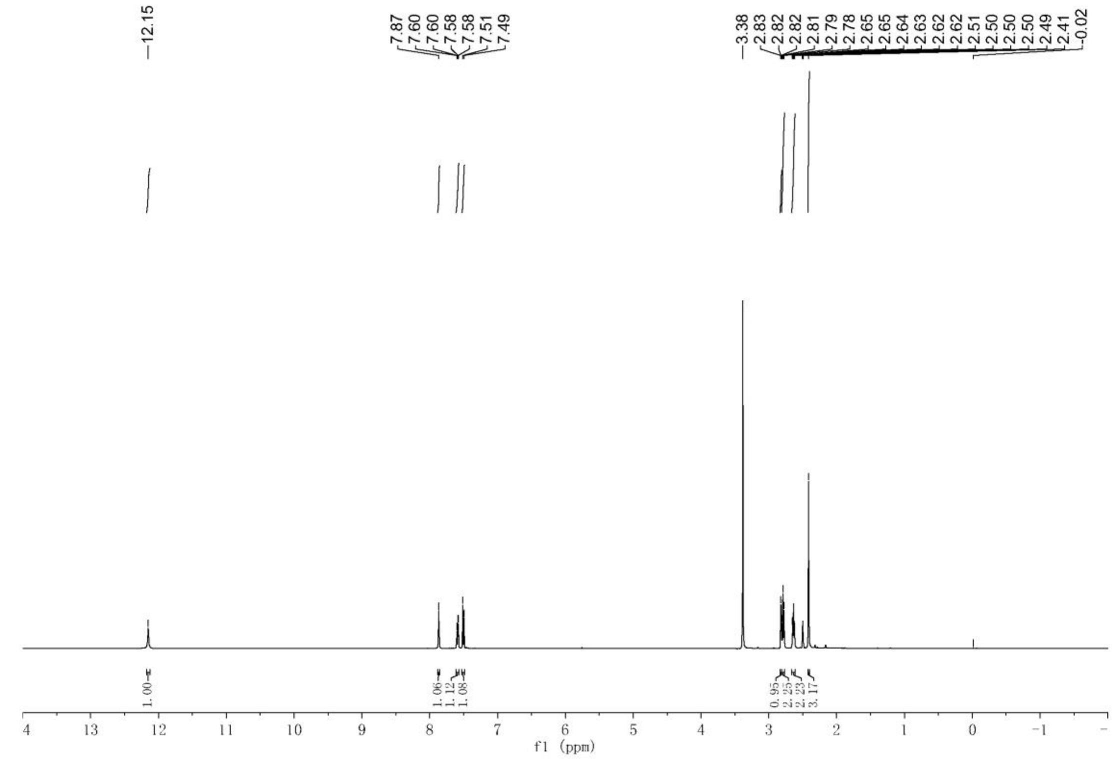

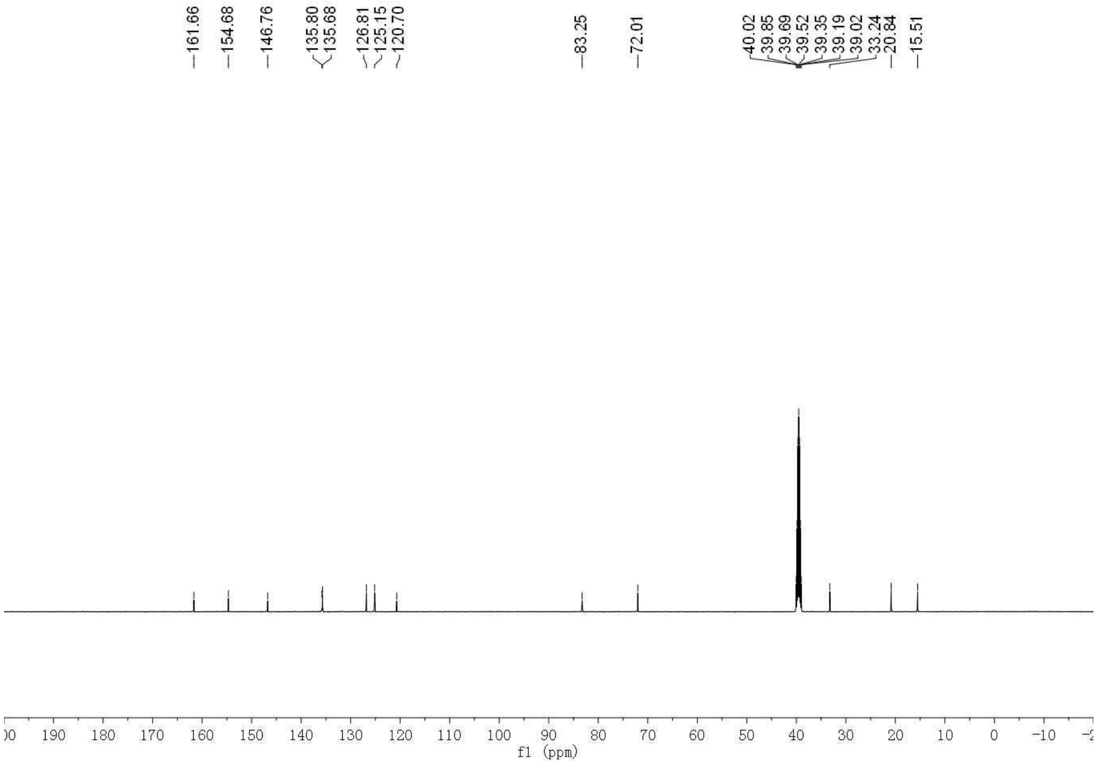

2,3-Dihydro-1-methylene-7-methyl-pyrrolo[2,1-*b*]quinazolin-9(1*H*)-one (9B)

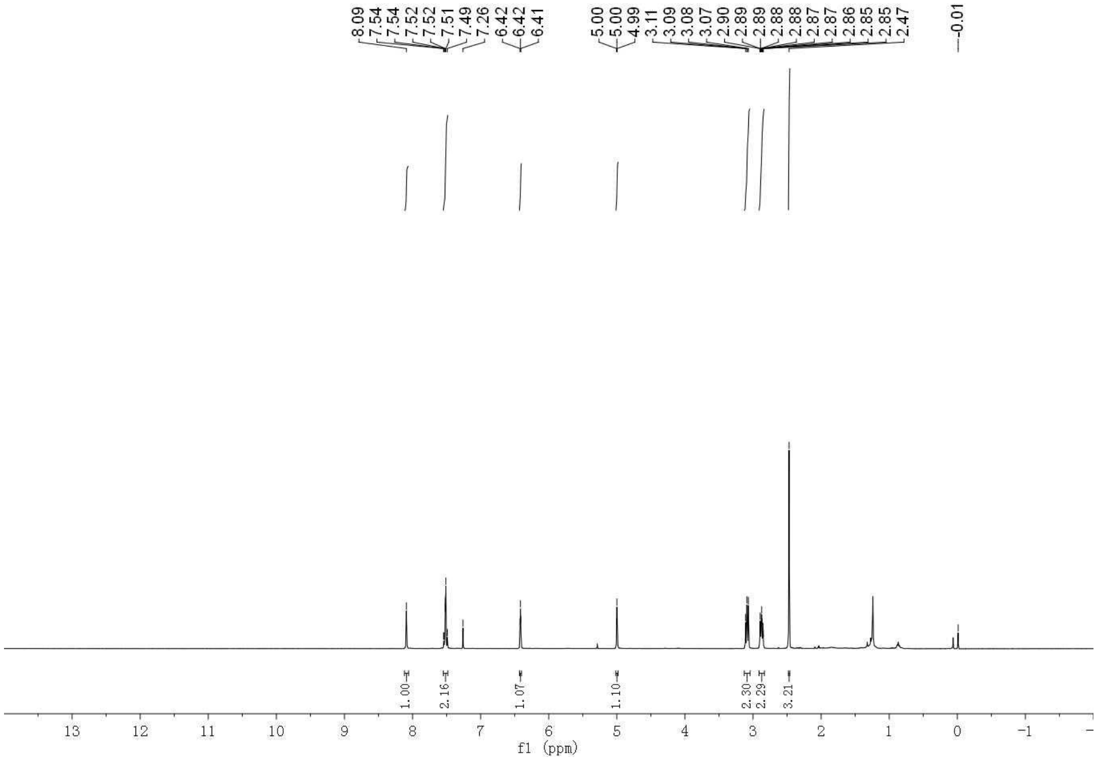

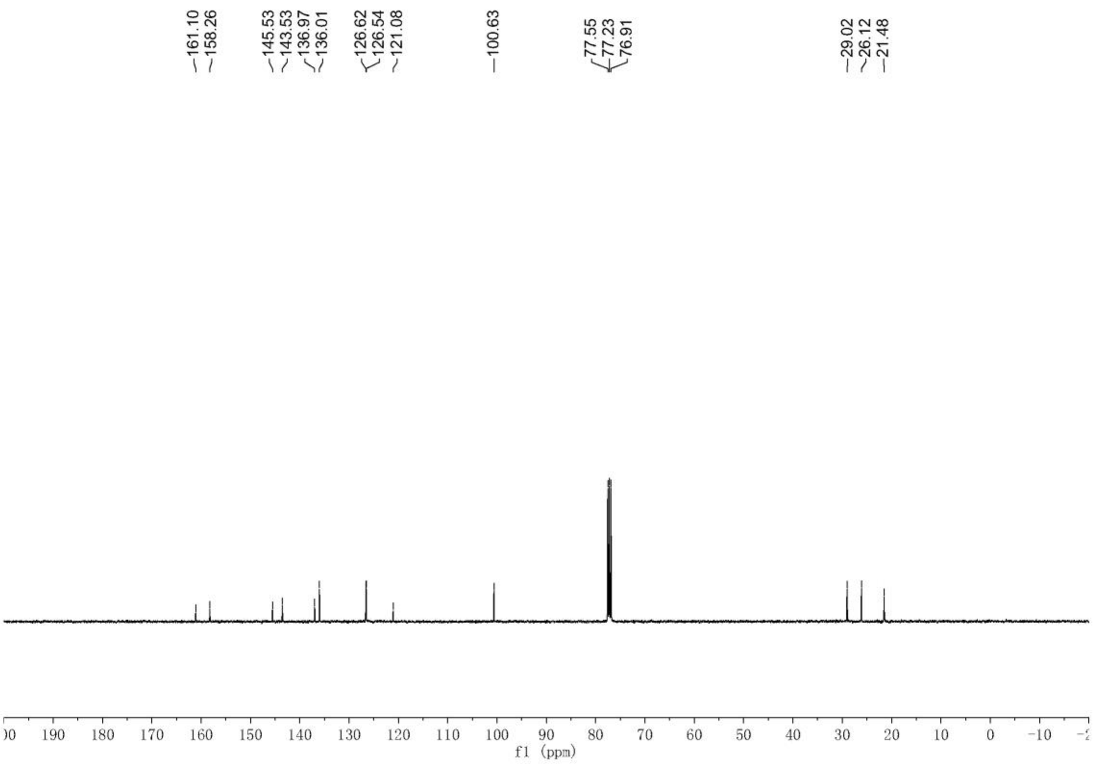

2-(3-Butynyl)-7-methyl-4(3*H*)-quinazolinone (8C)

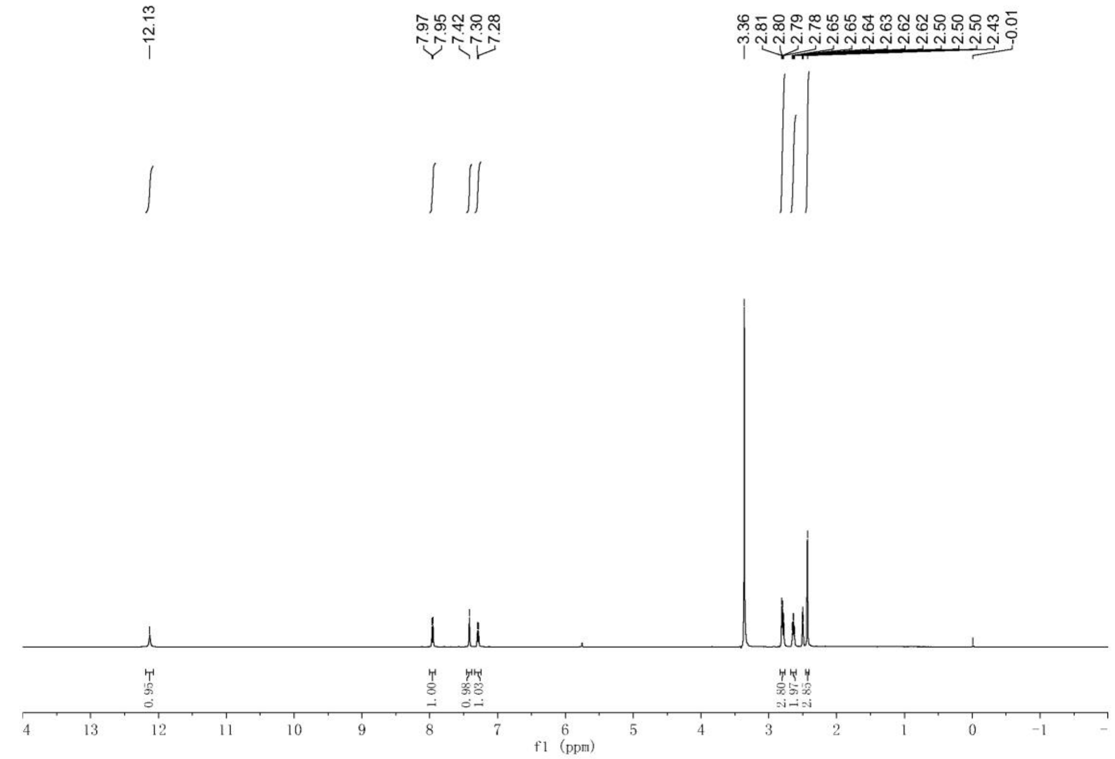

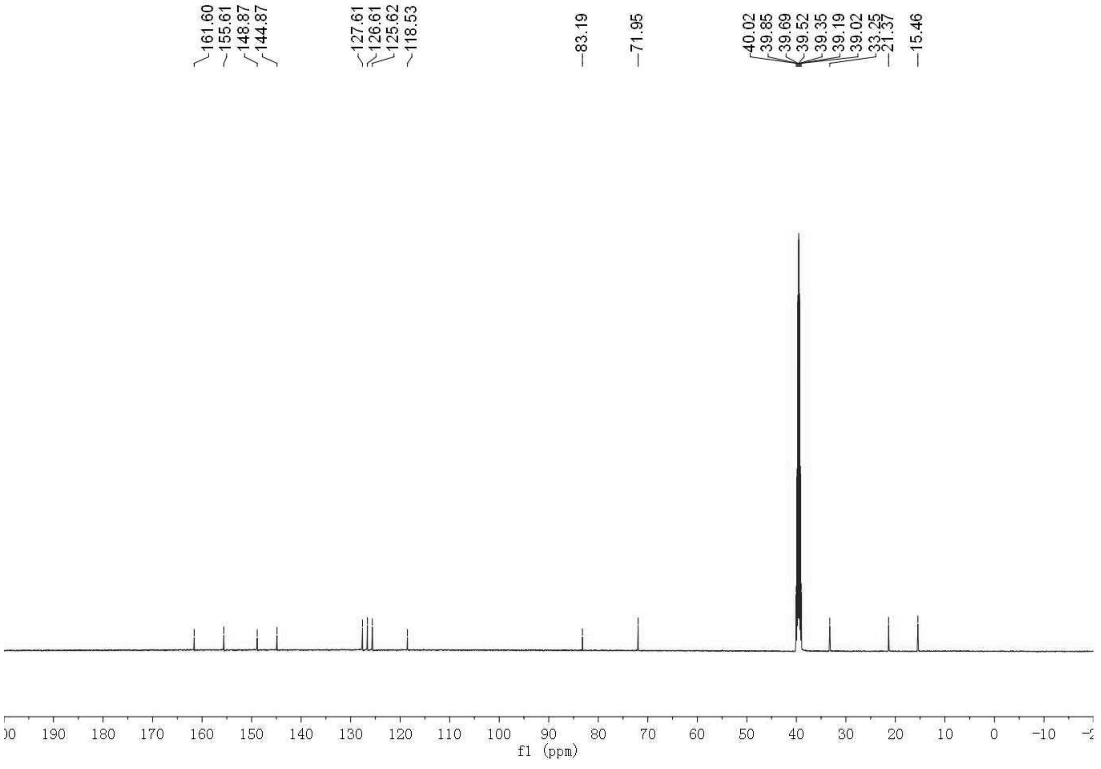

2,3-Dihydro-1-methylene-6-methyl-pyrrolo[2,1-*b*]quinazolin-9(1*H*)-one (9C)

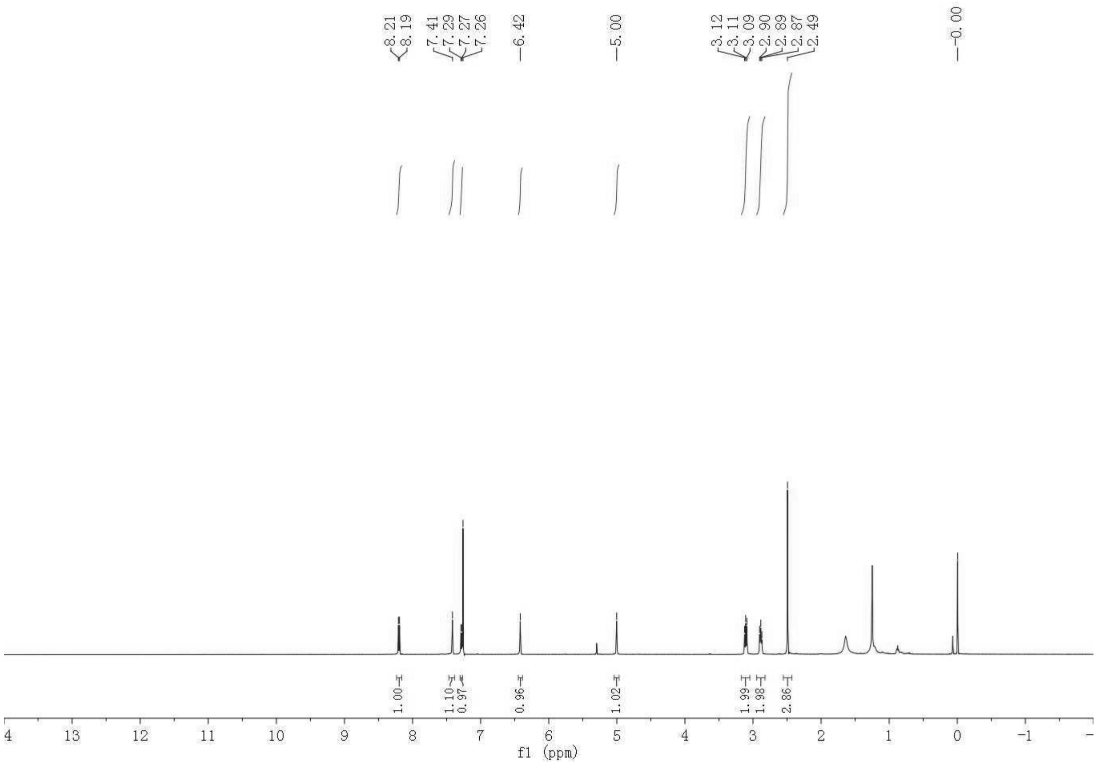

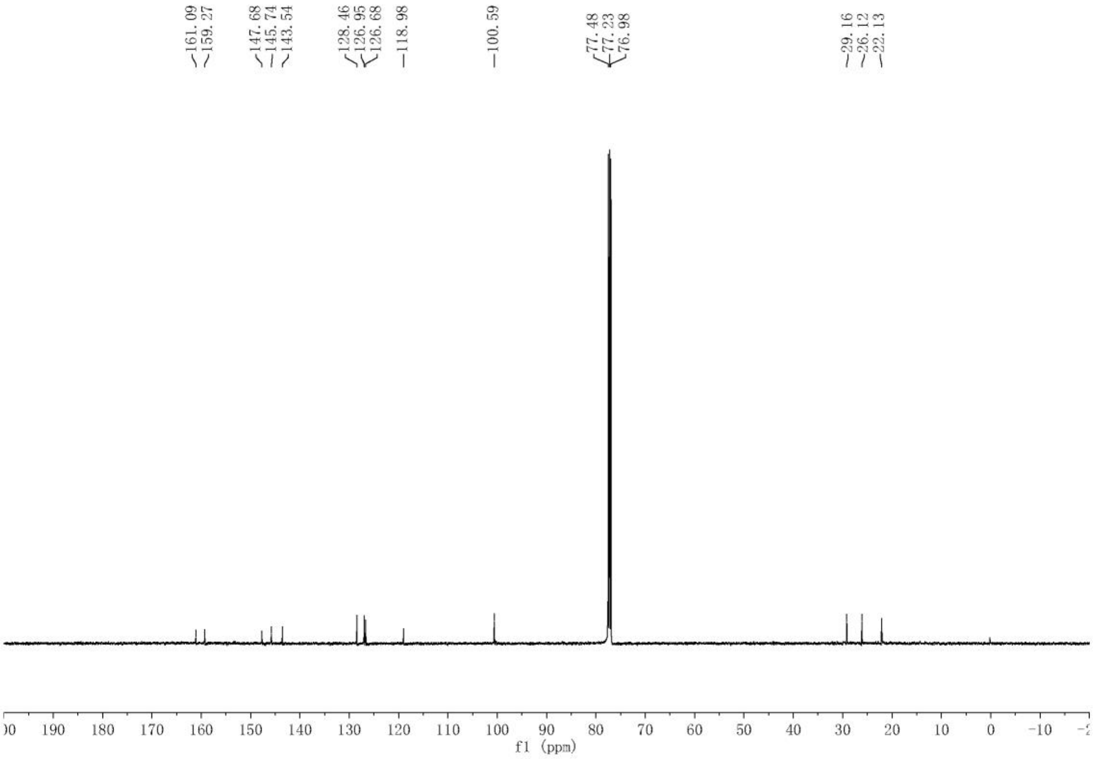

2-(3-Butynyl)-6-methoxy-4(3*H*)-quinazolinone (8D)

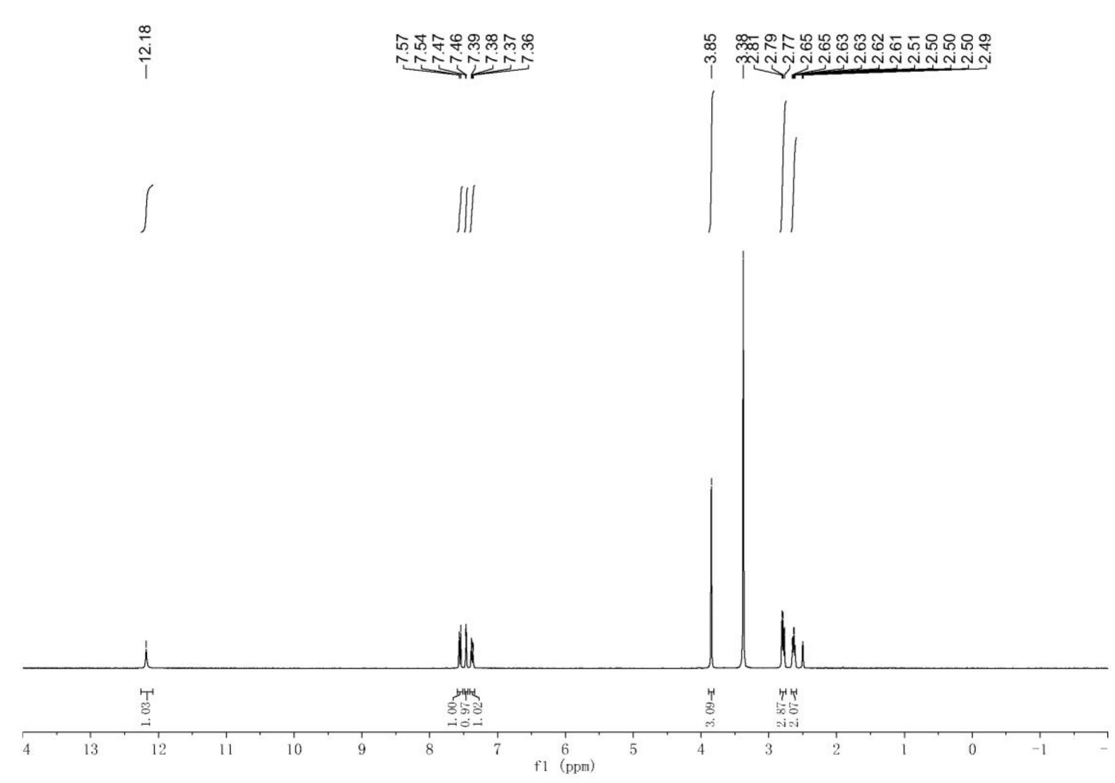

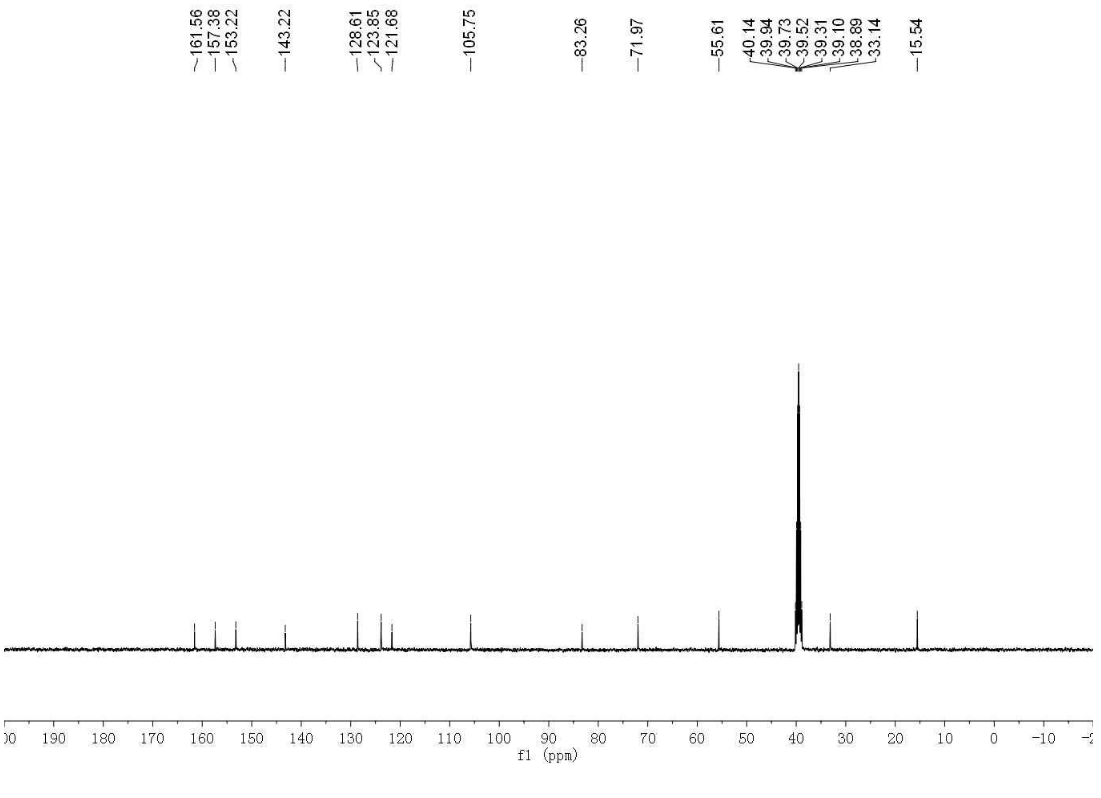

2,3-Dihydro-1-methylene-7-methoxy-pyrrolo[2,1-*b*]quinazolin-9(1*H*)-one (9D)

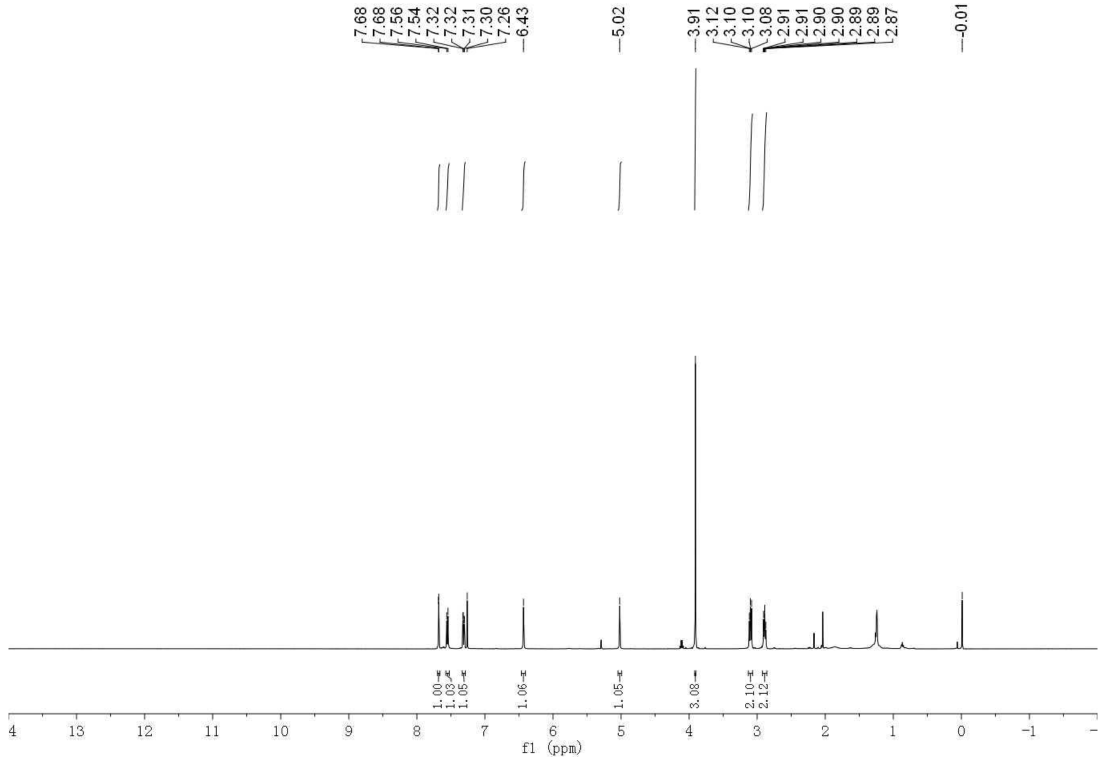

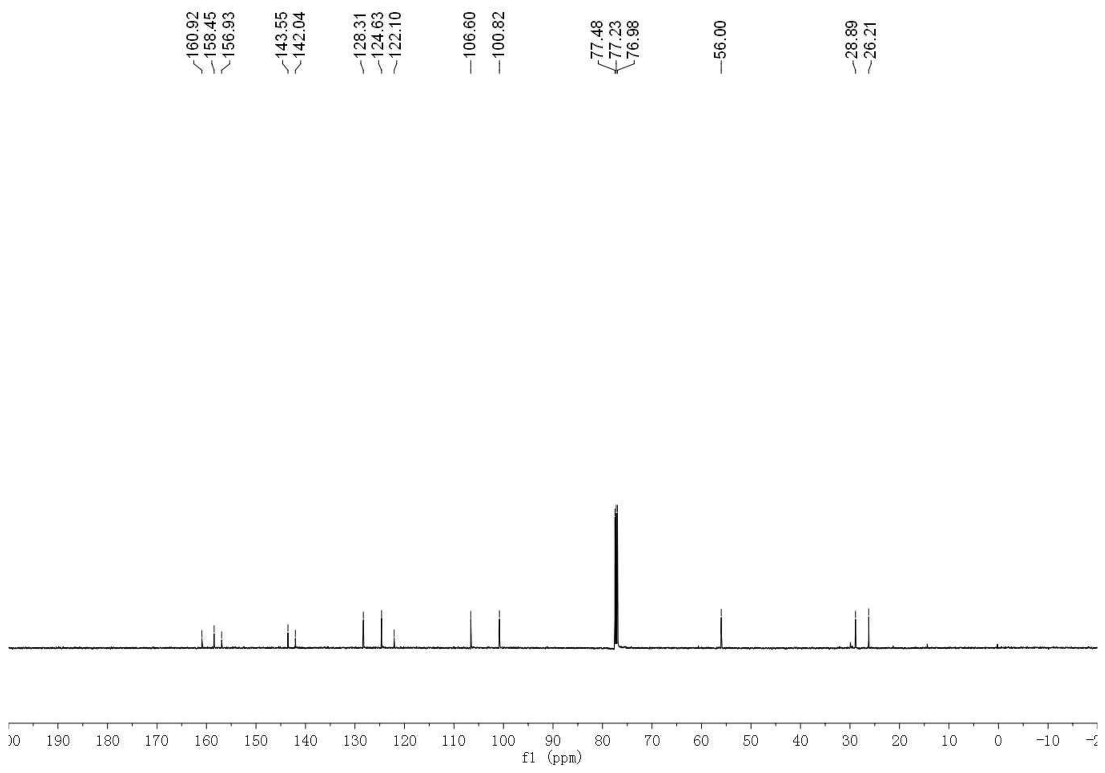

2-(3-Butynyl)-8-methoxy-4(3*H*)-quinazolinone (8E)

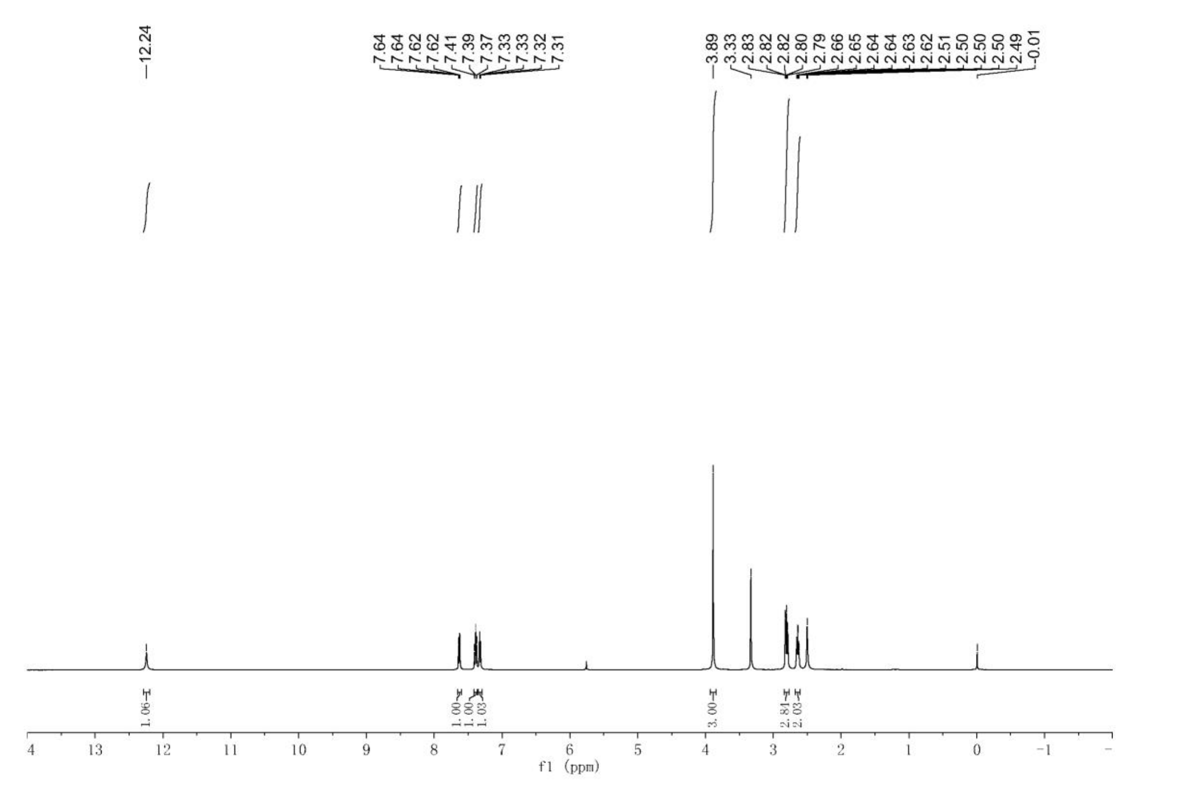

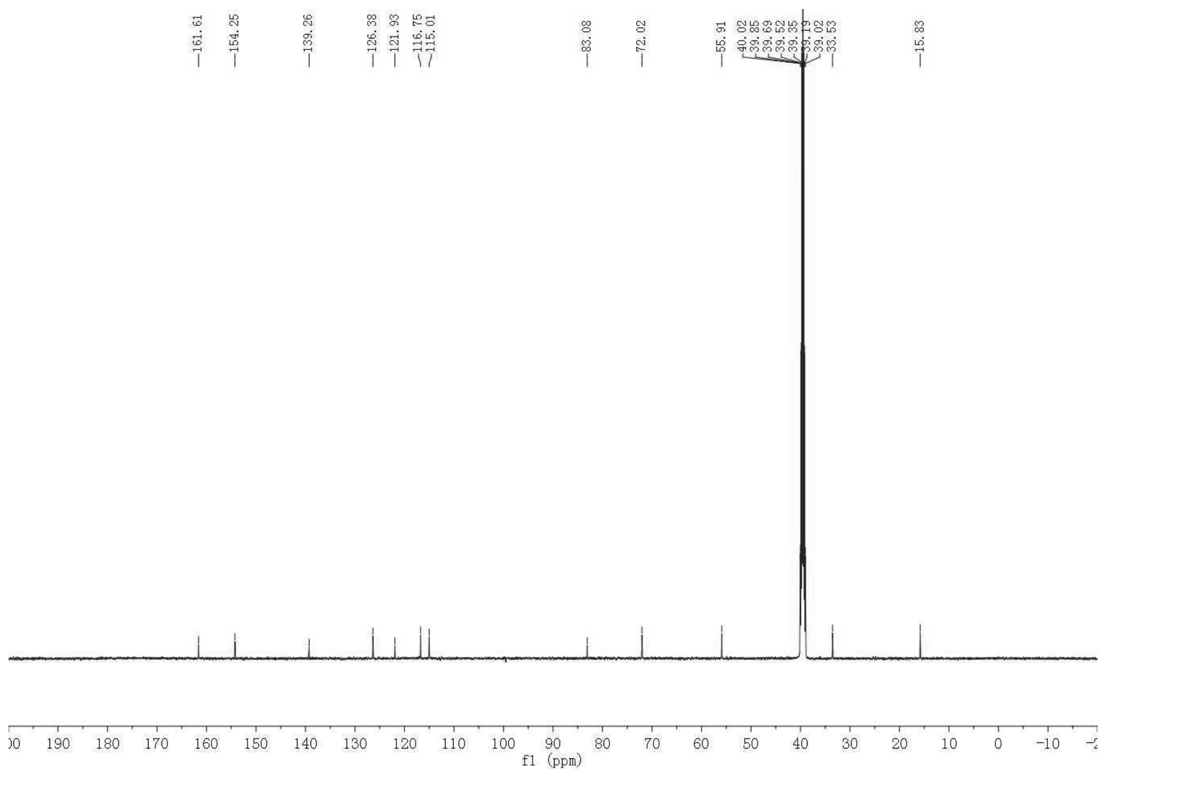

2,3-Dihydro-1-methylene-5-methoxy-pyrrolo[2,1-*b*]quinazolin-9(1*H*)-one (9E)

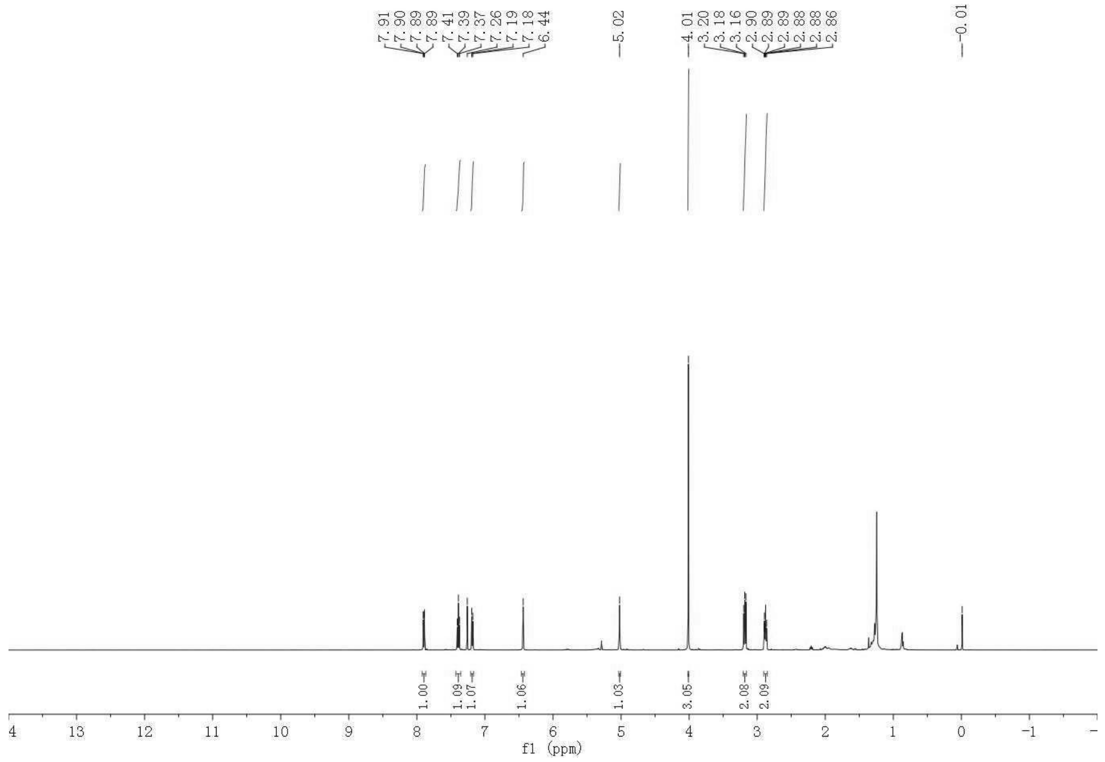

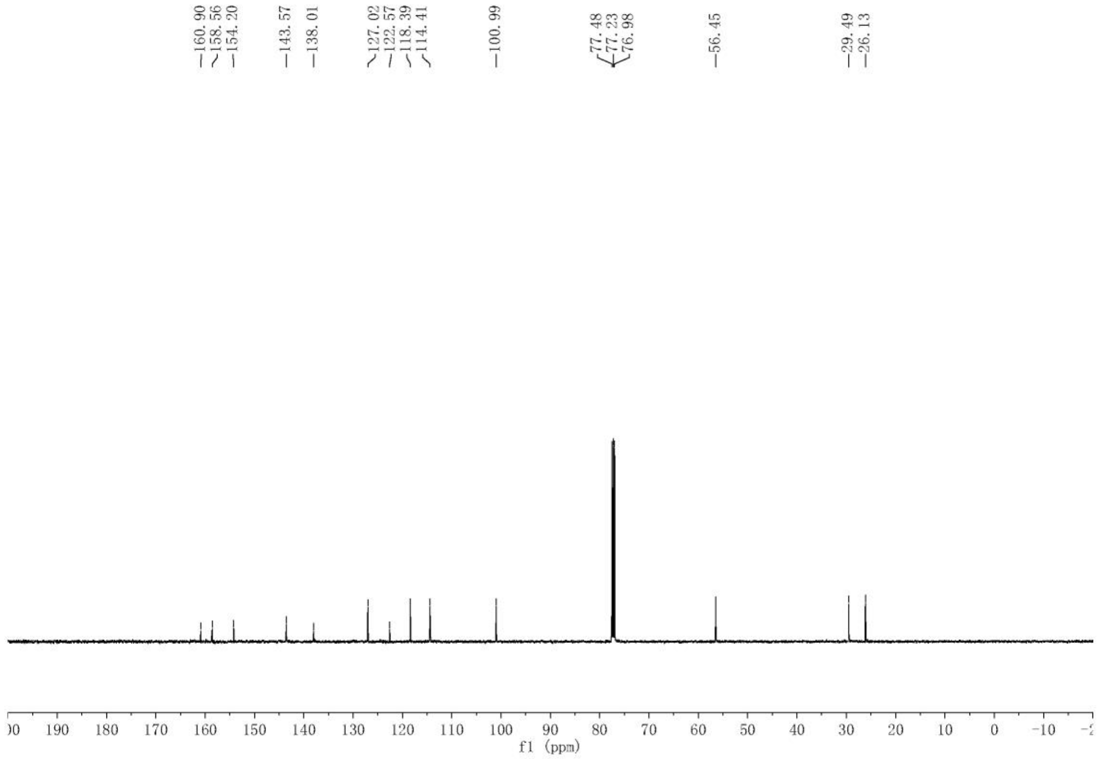

2-(3-Butynyl)-5-fluoro-4(3*H*)-quinazolinone (8F)

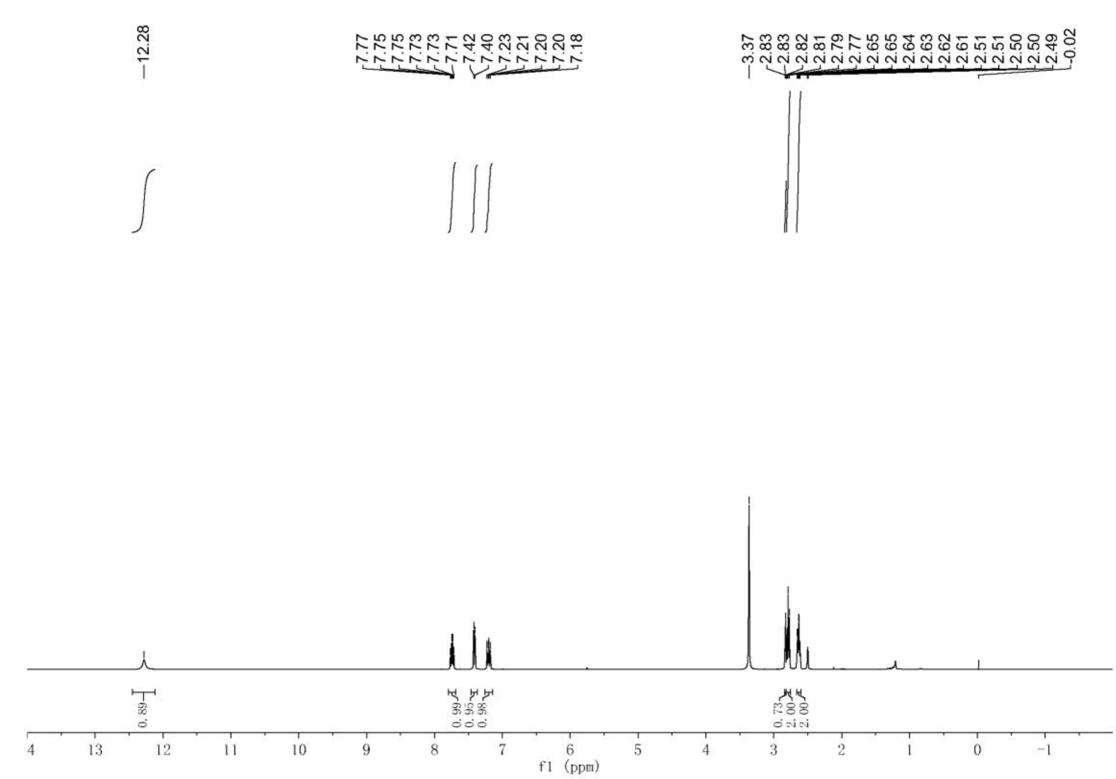

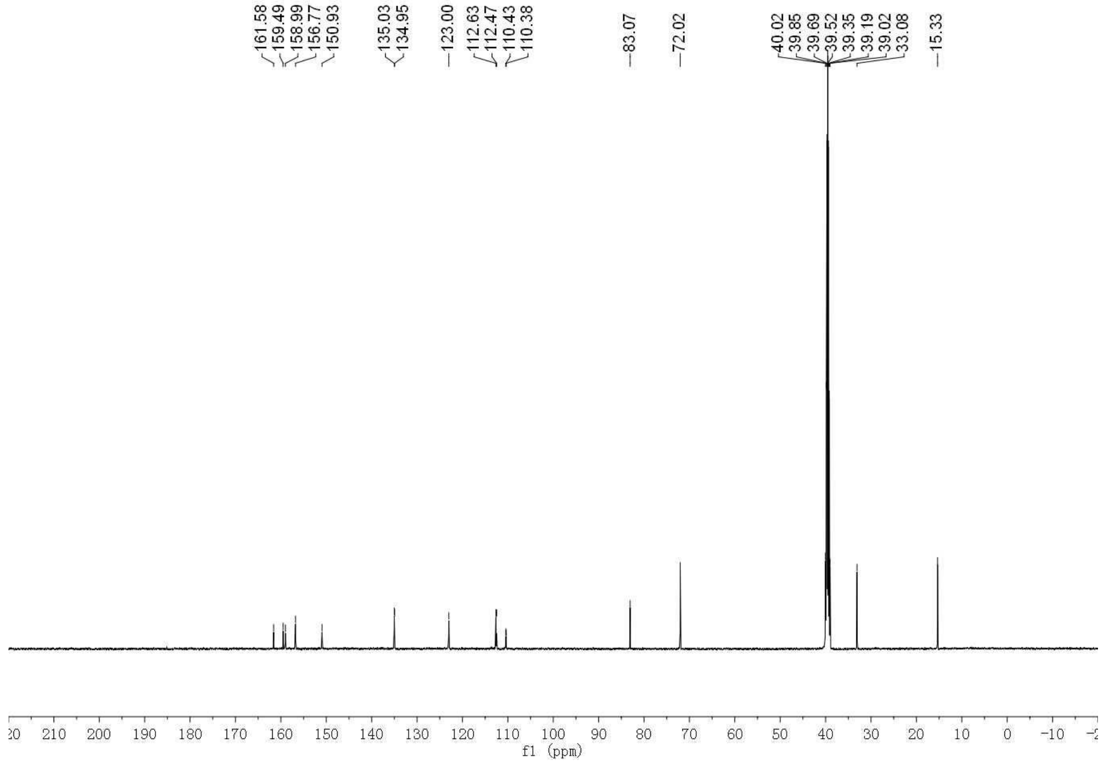

2,3-Dihydro-1-methylene-8-fluoro-pyrrolo[2,1-*b*]quinazolin-9(1*H*)-one (9F)

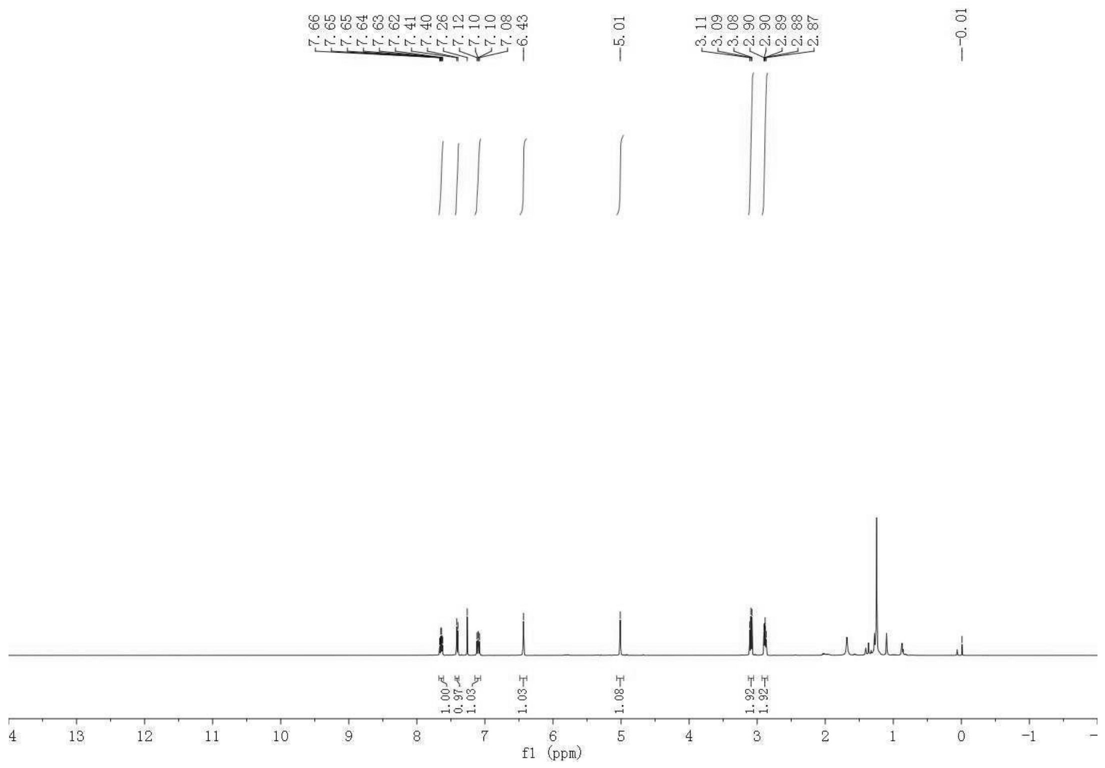

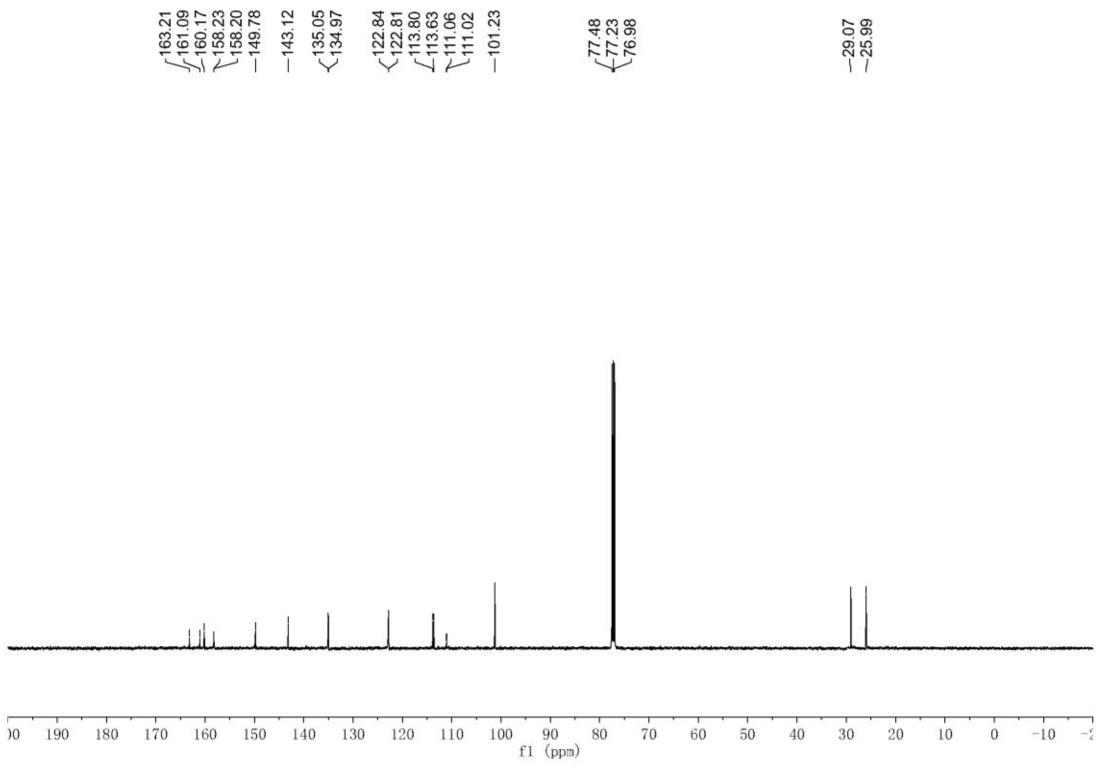

2-(3-Butynyl)-5-chloro-4(3*H*)-quinazolinone (8G)

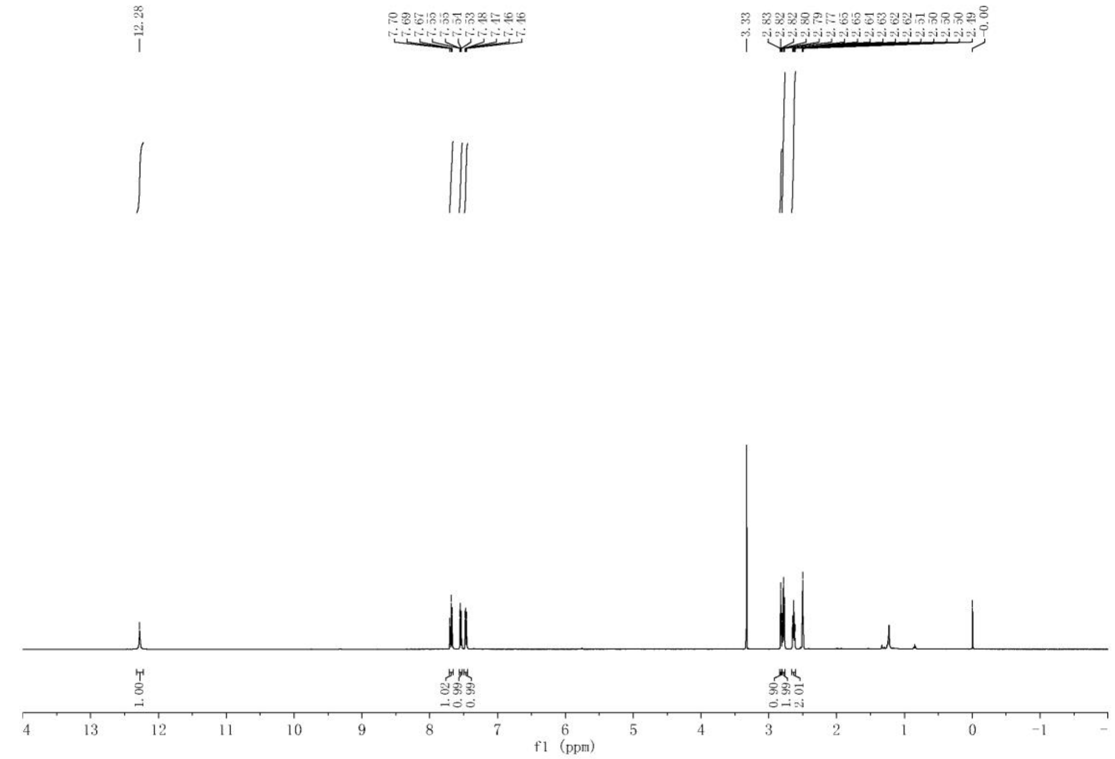

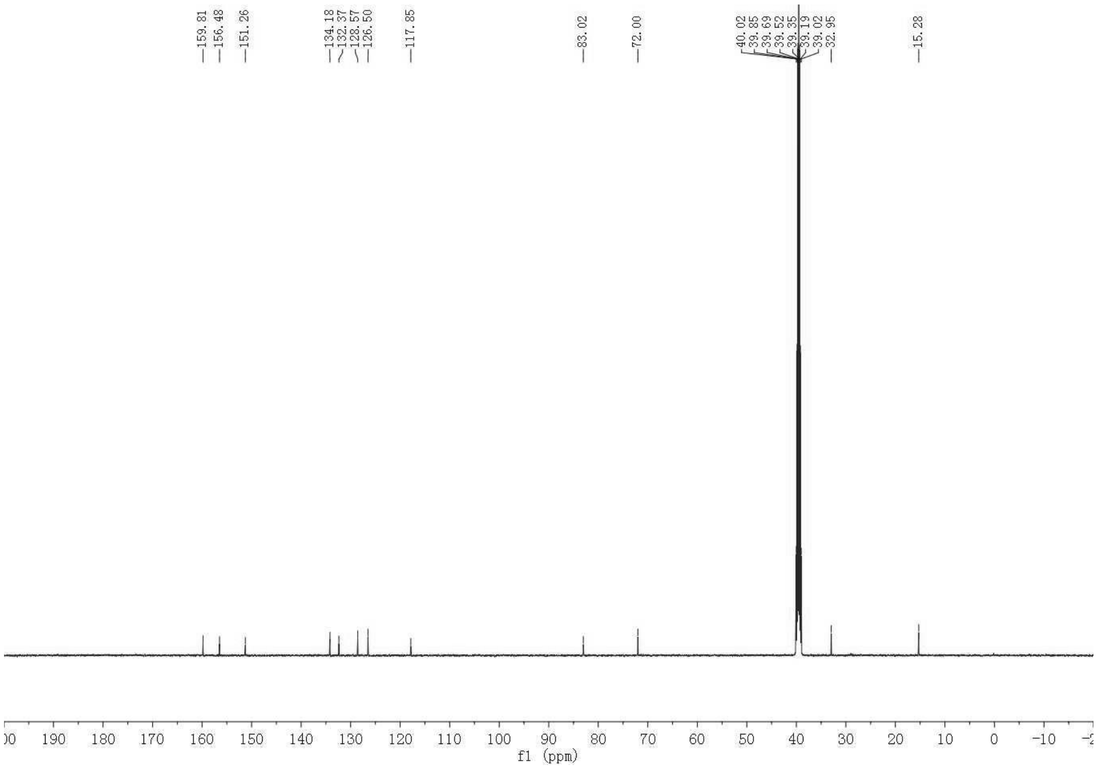

2,3-Dihydro-1-methylene-8-chloro-pyrrolo[2,1-*b*]quinazolin-9(1*H*)-one (9G)

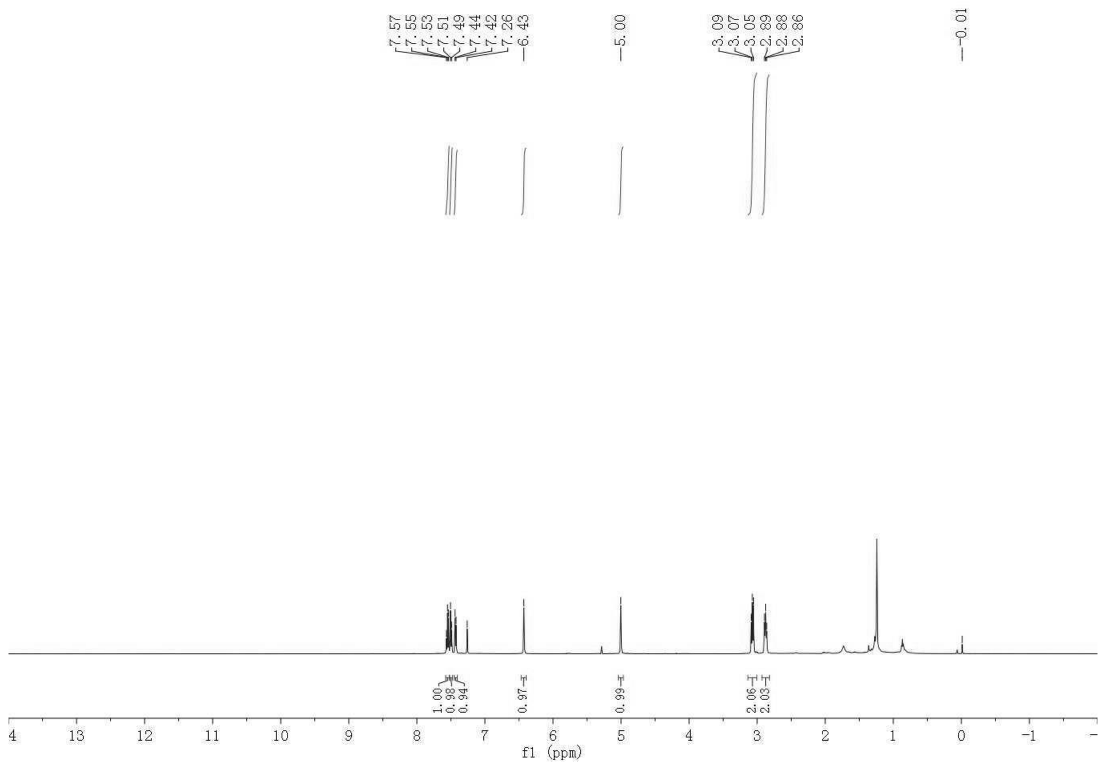

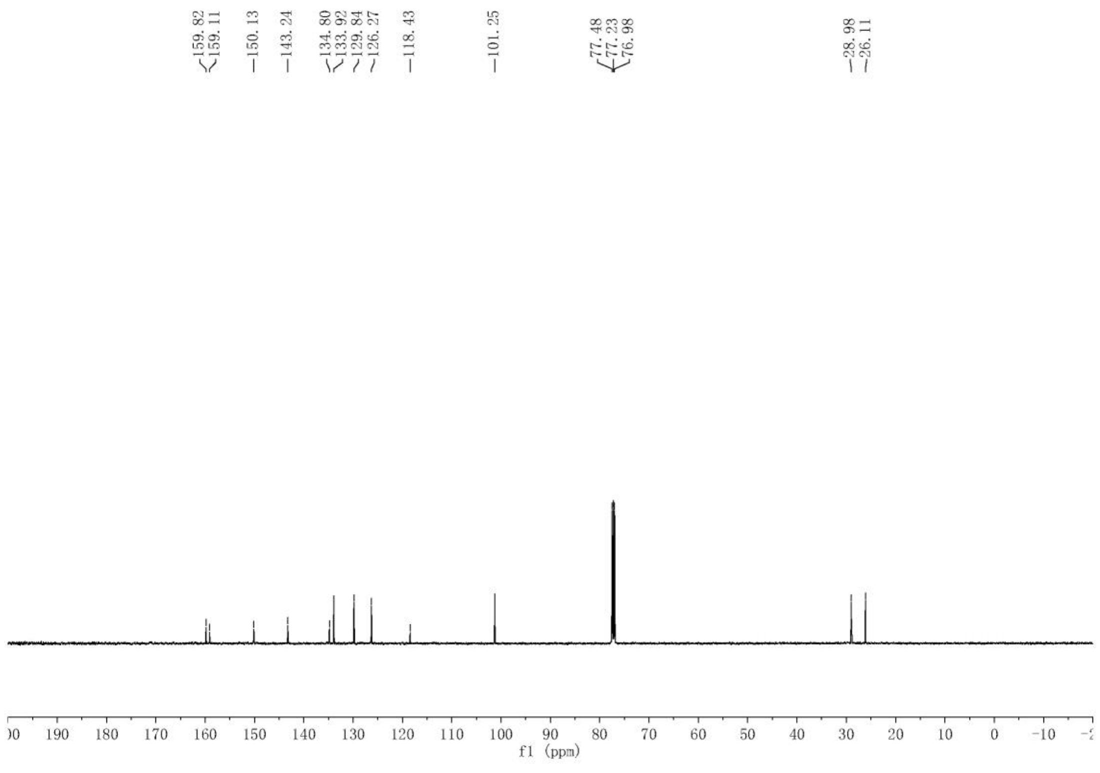

2-(3-Butynyl)-6-chloro-4(3*H*)-quinazolinone (8H)

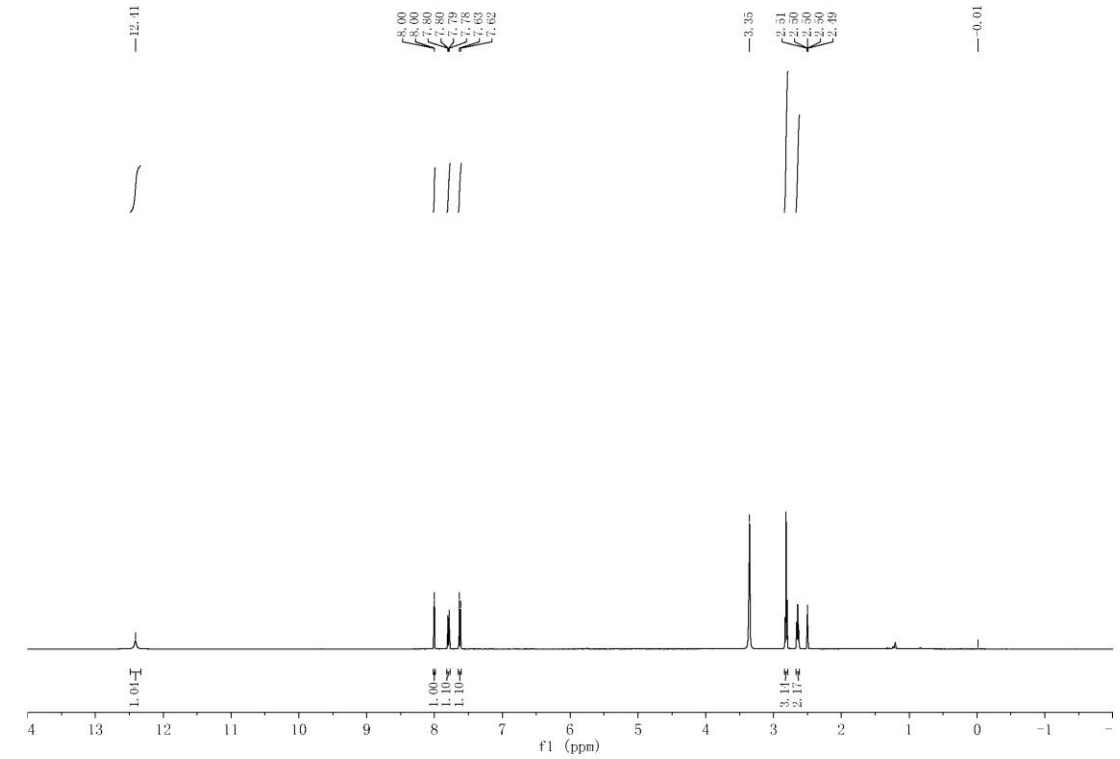

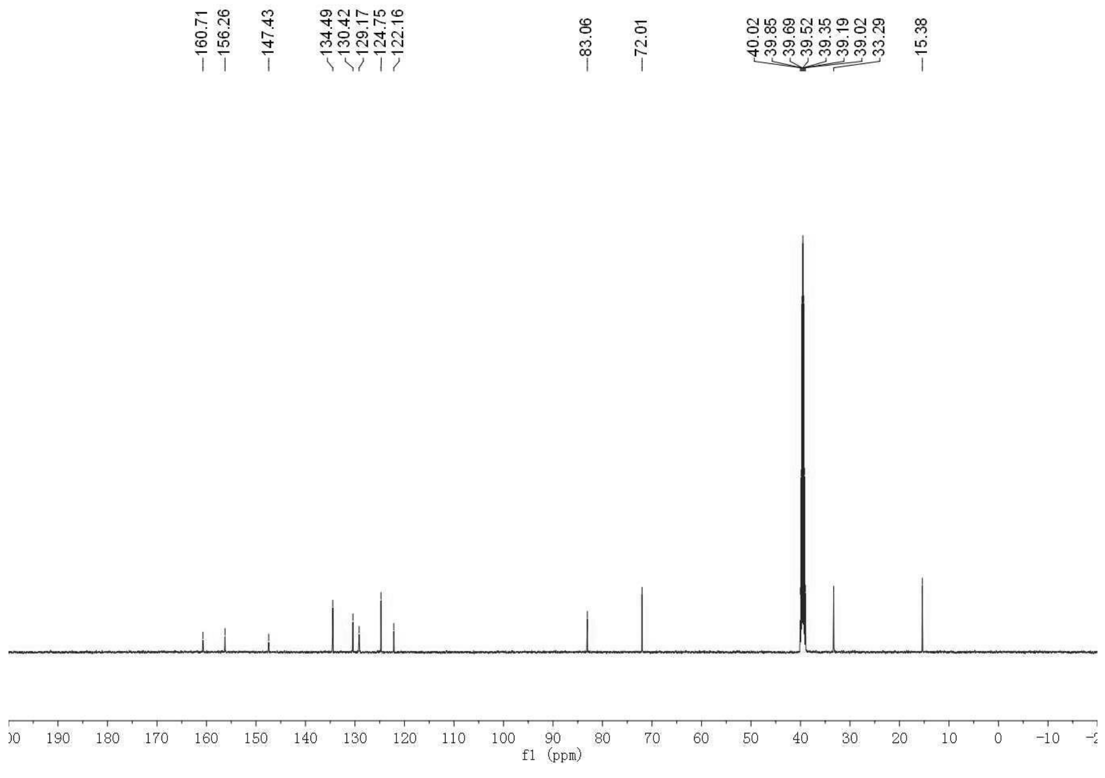

2,3-Dihydro-1-methylene-7-chloro-pyrrolo[2,1-*b*]quinazolin-9(1*H*)-one (9H)

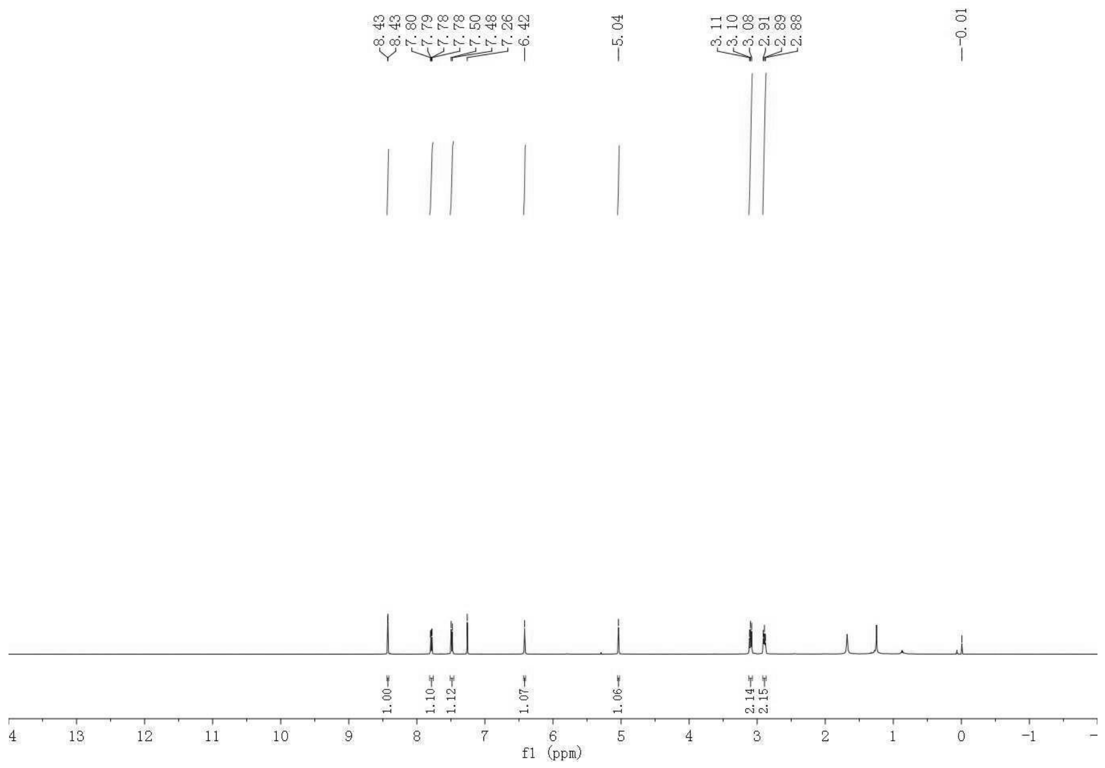

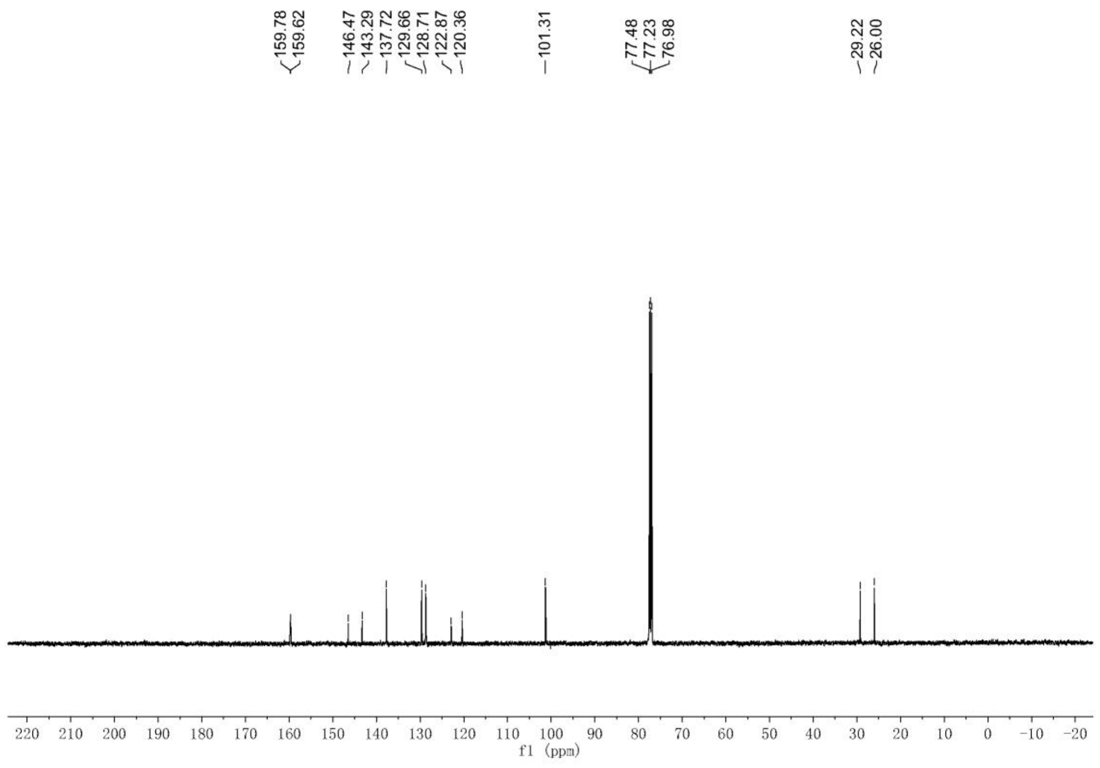

2-(3-Butynyl)-7-chloro-4(3*H*)-quinazolinone (8l)

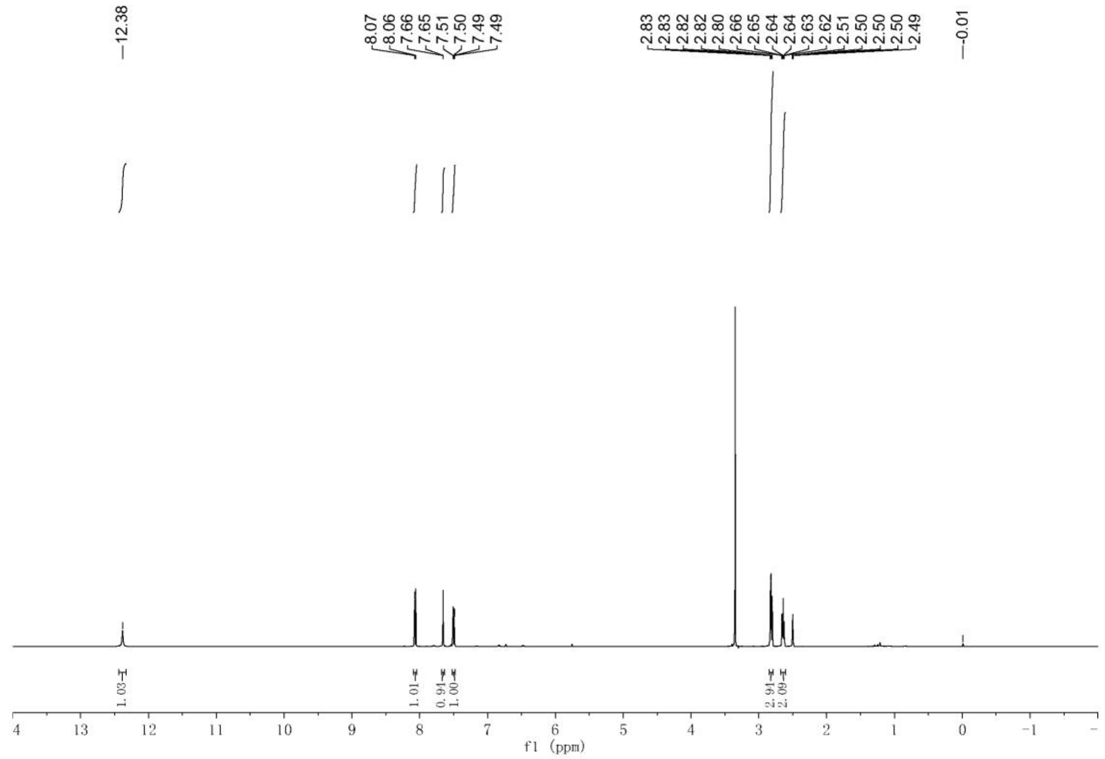

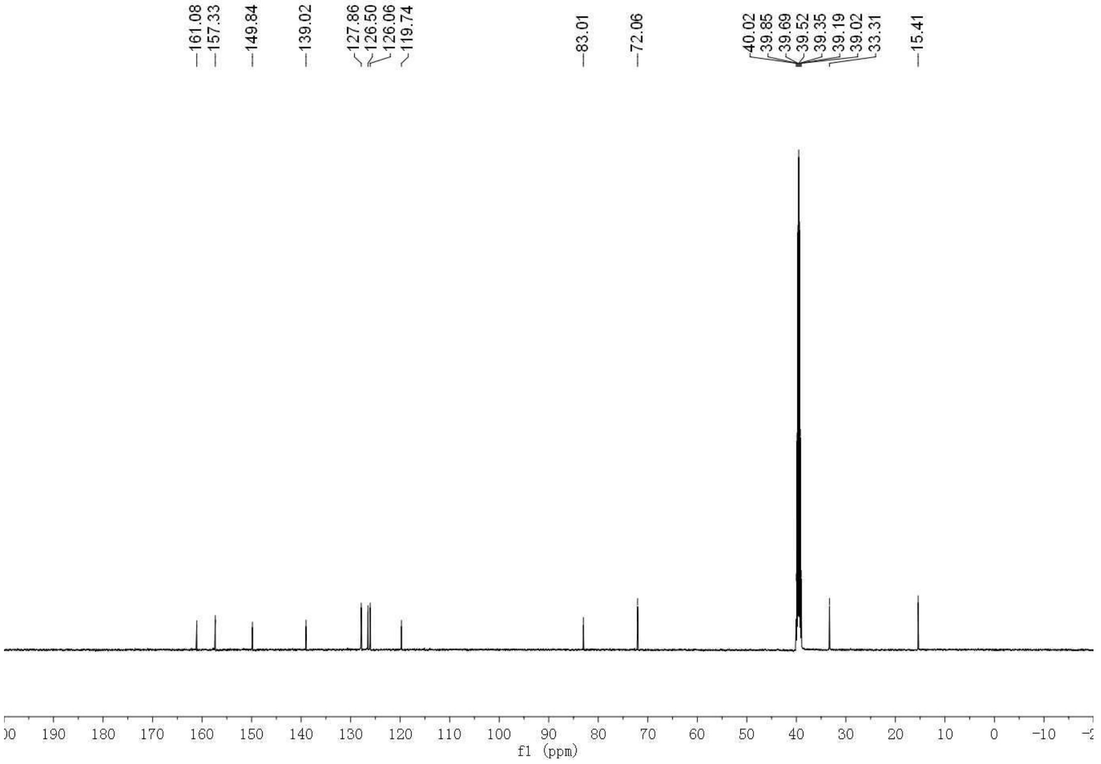

2,3-Dihydro-1-methylene-6-chloro-pyrrolo[2,1-*b*]quinazolin-9(1*H*)-one (9l)

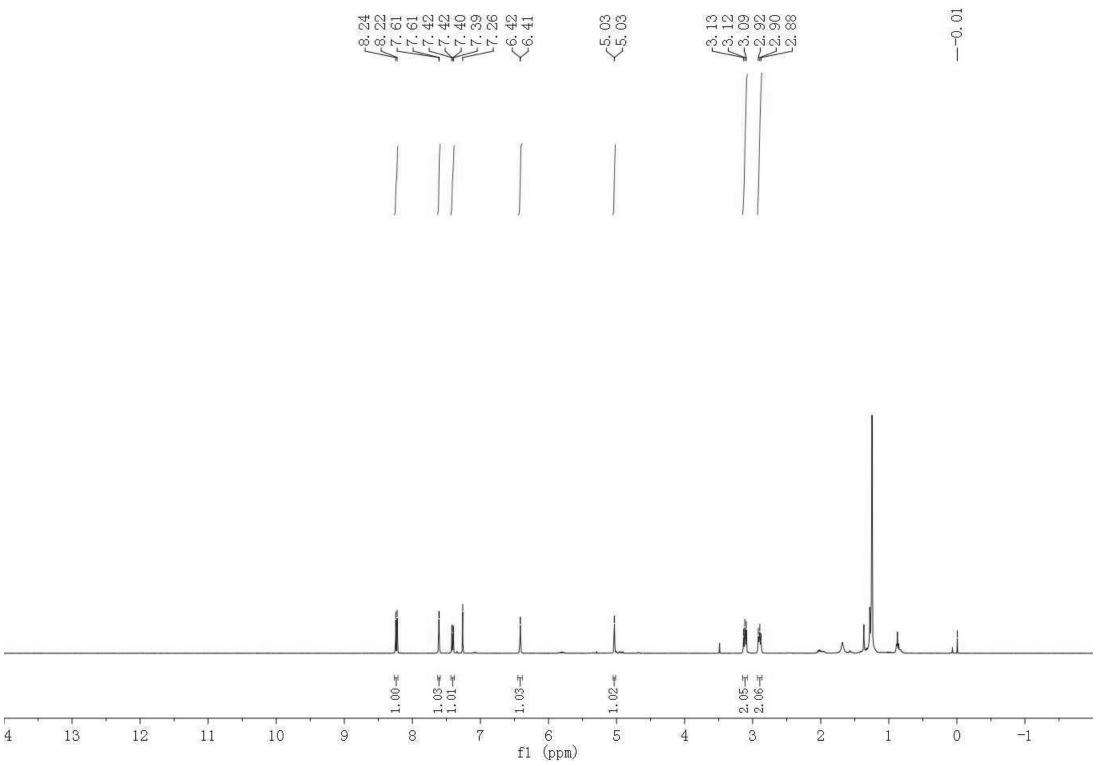

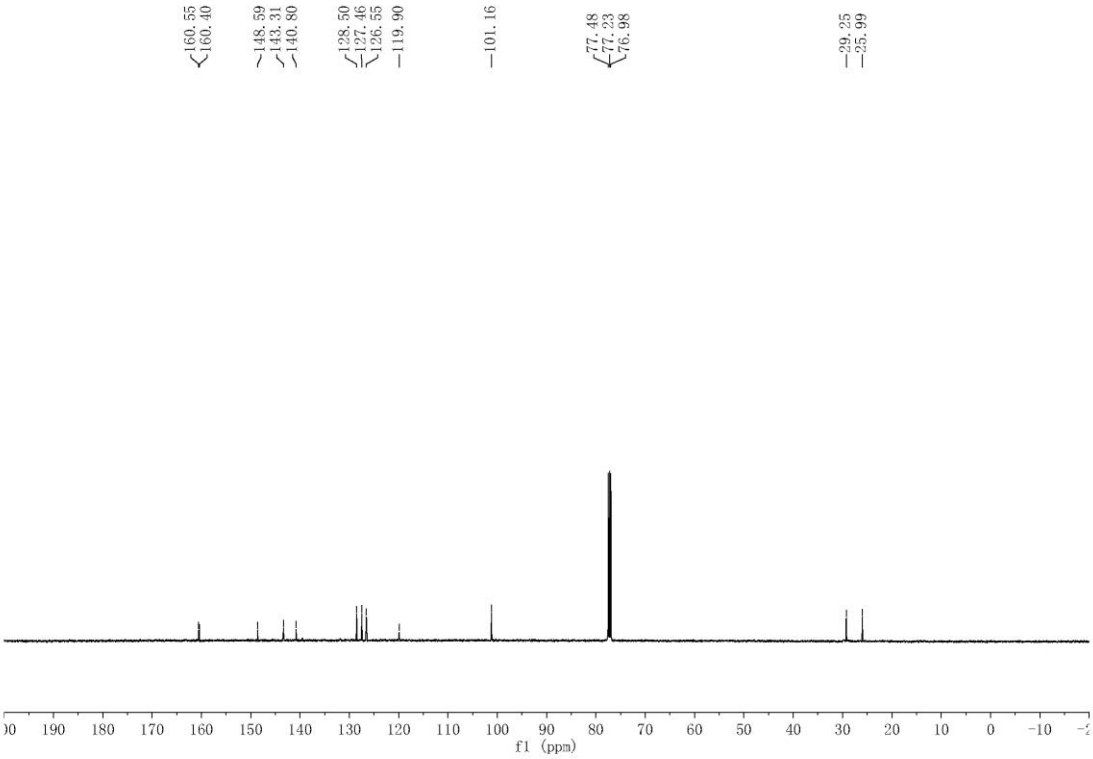

2-(3-Butynyl)-6-bromo-4(3*H*)-quinazolinone (8J)

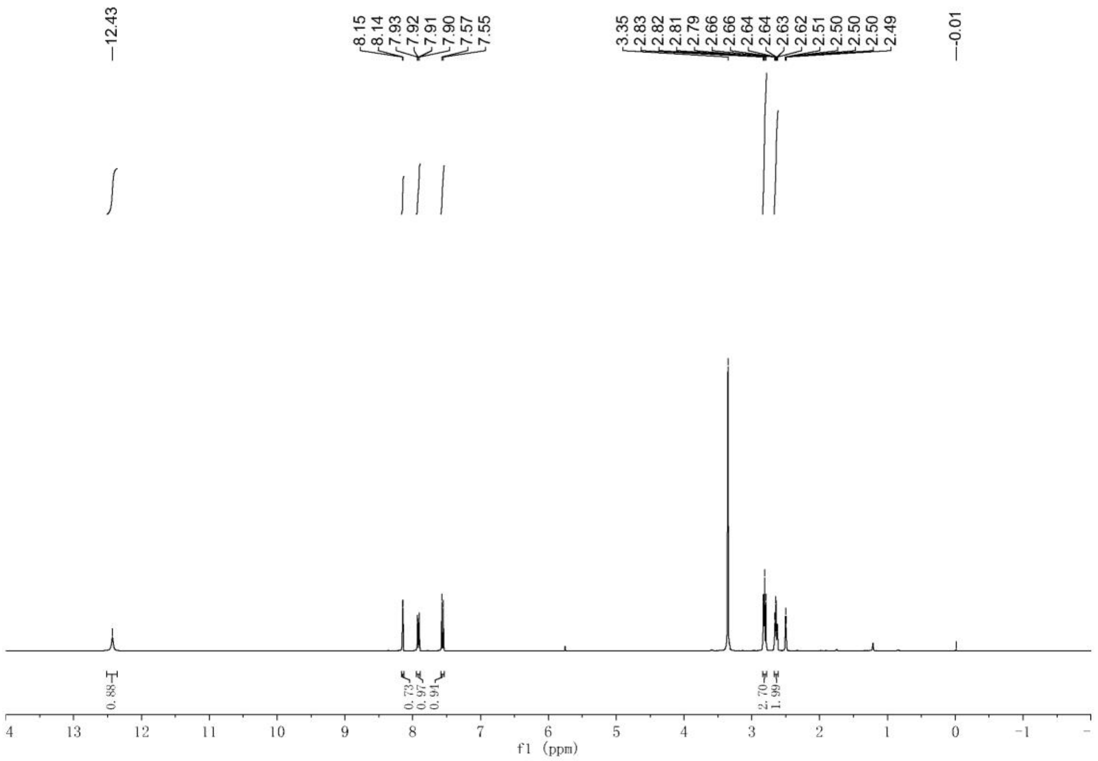

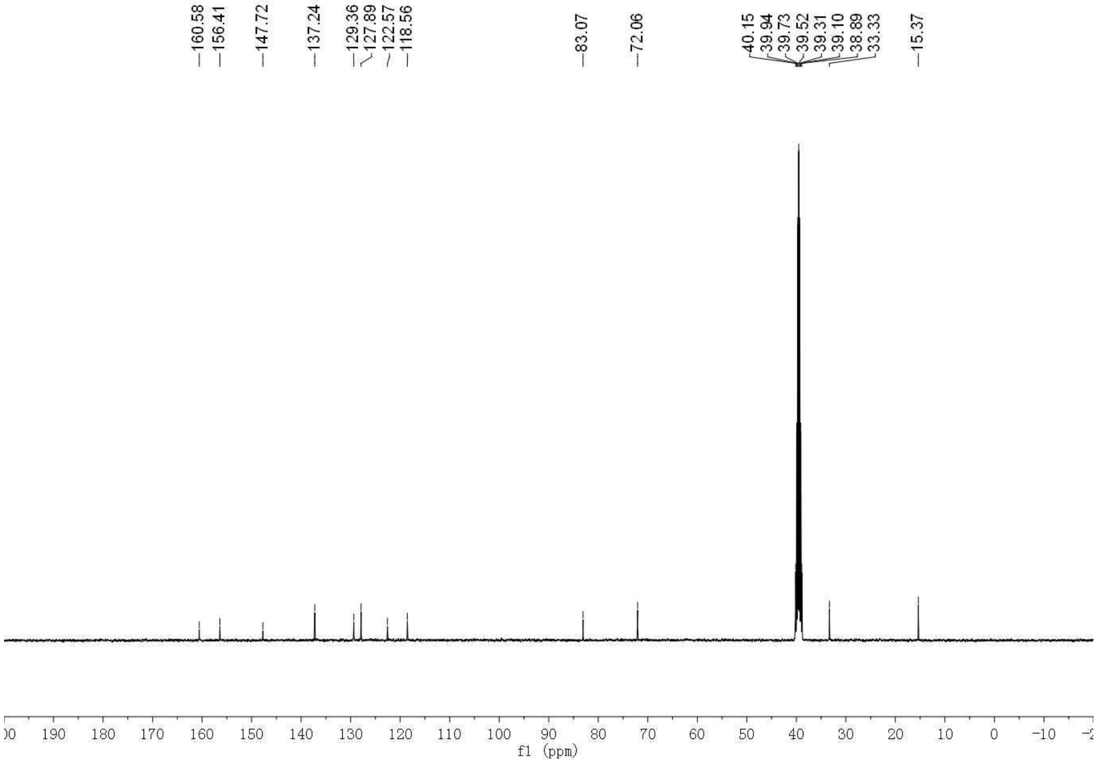

2,3-Dihydro-1-methylene-7-bromo-pyrrolo[2,1-*b*]quinazolin-9(1*H*)-one (9J)

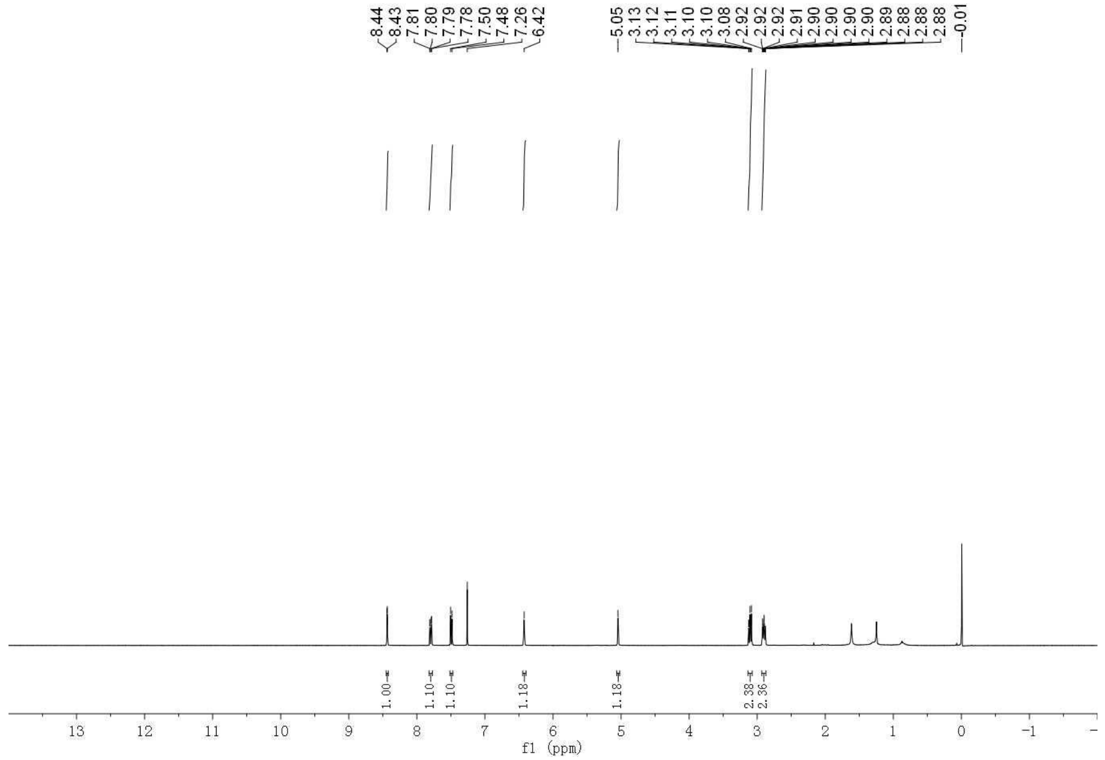

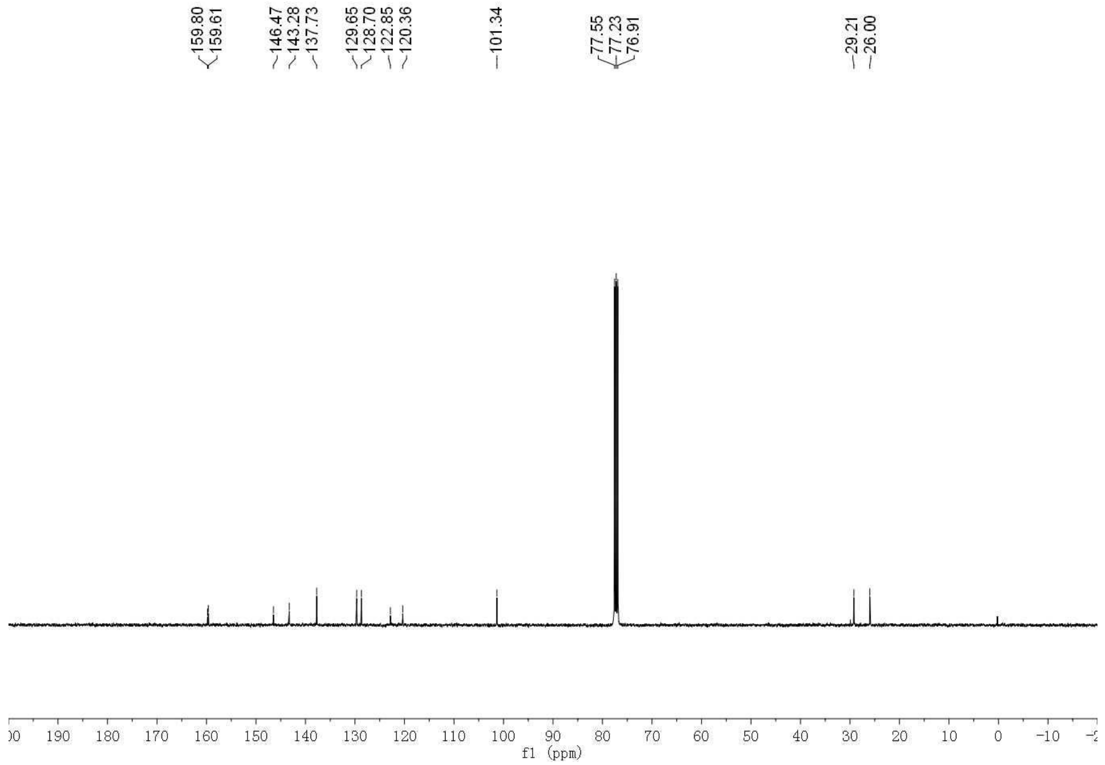

2-(3-Butynyl)-7-phenyl-4(3*H*)-quinazolinone (8K)

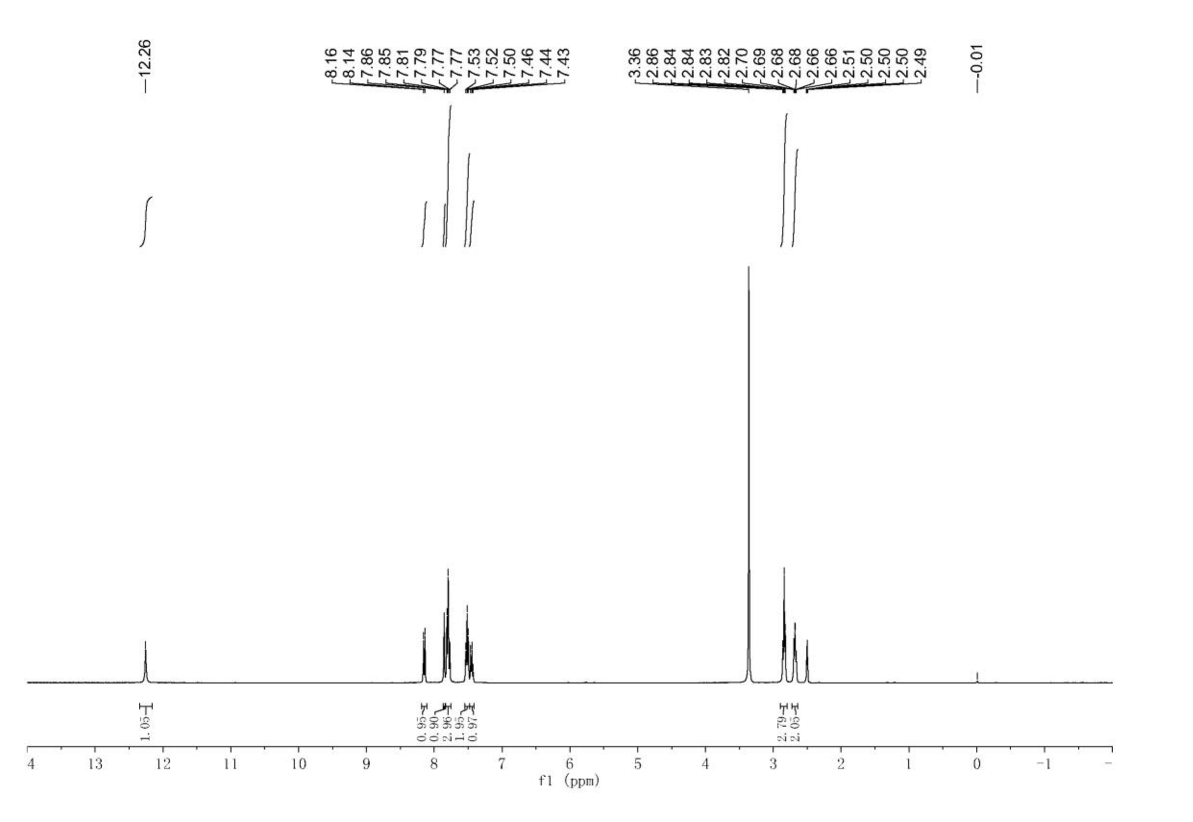

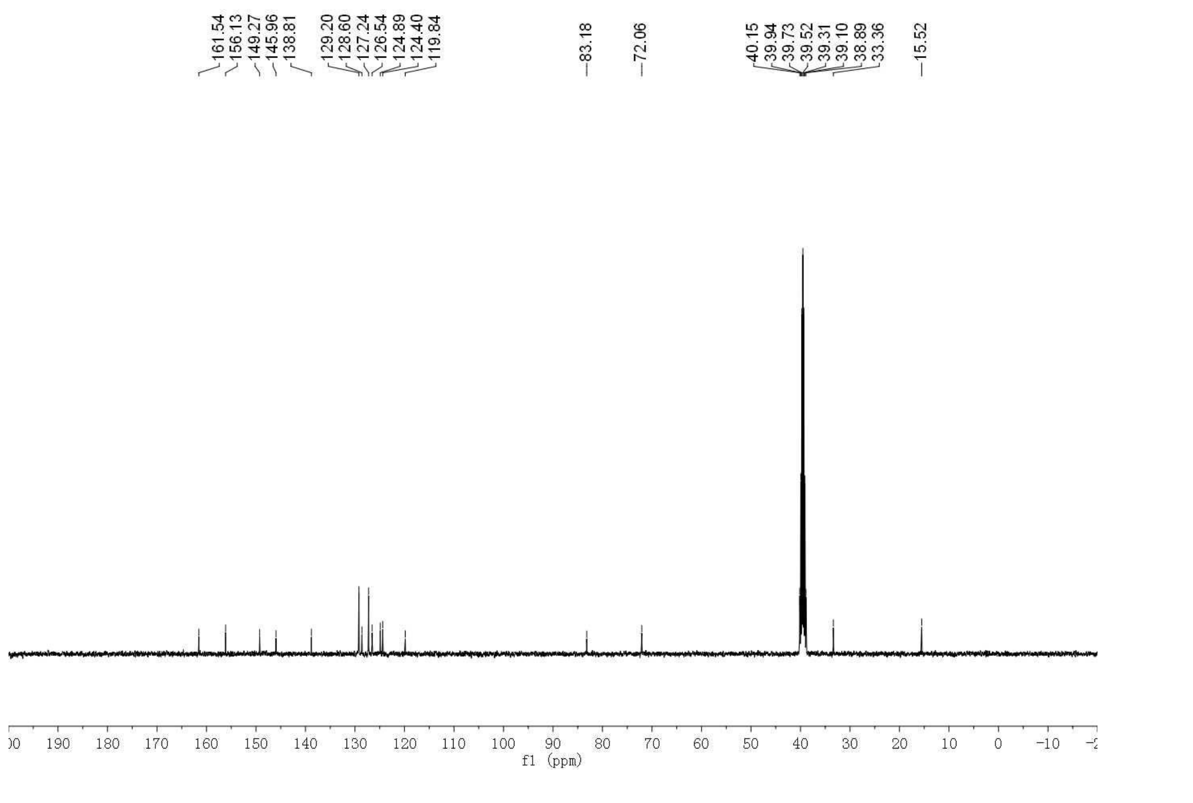

2,3-Dihydro-1-methylene-6-phenyl -pyrrolo[2,1-*b*]quinazolin-9(1*H*)-one (9K)

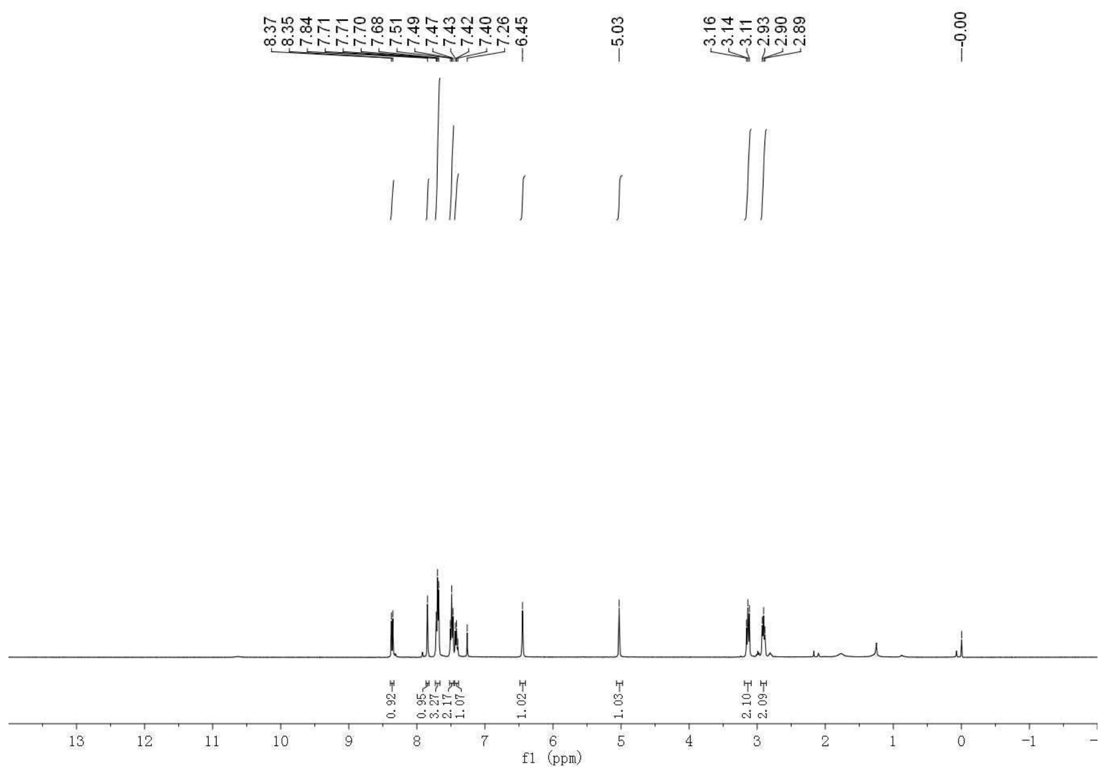

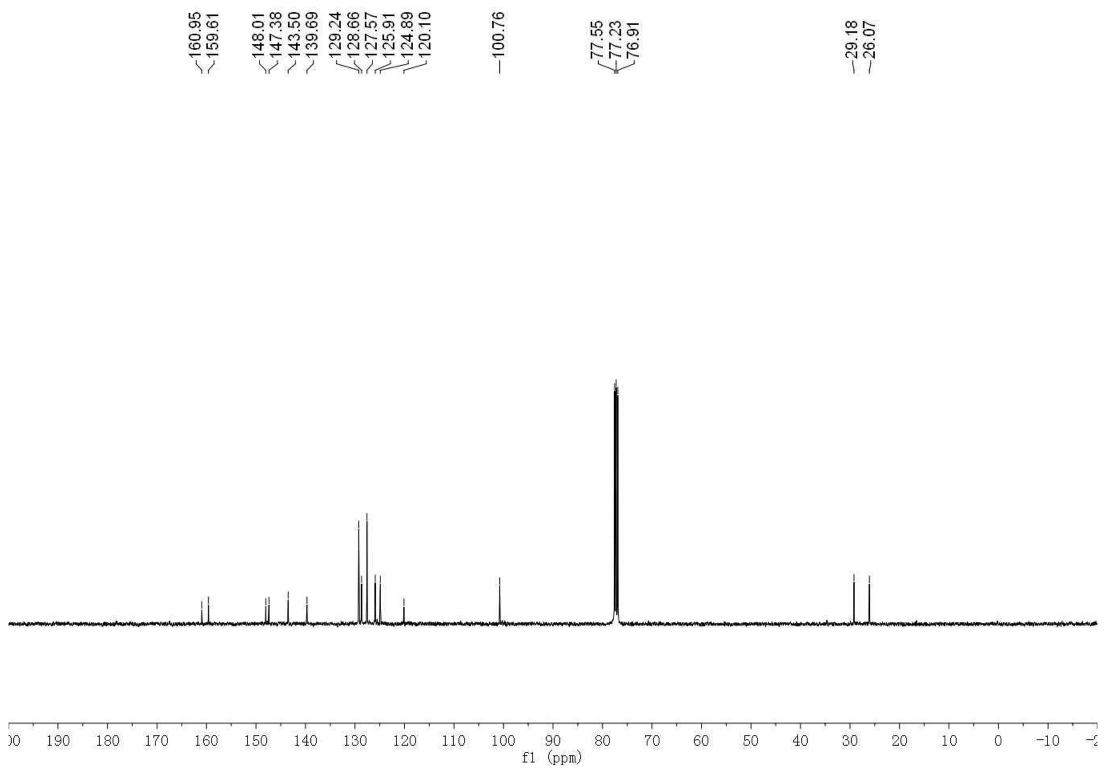

2-(3-Butynyl)-4(3*H*)-benzo[*g*]quinazolinone (8L)

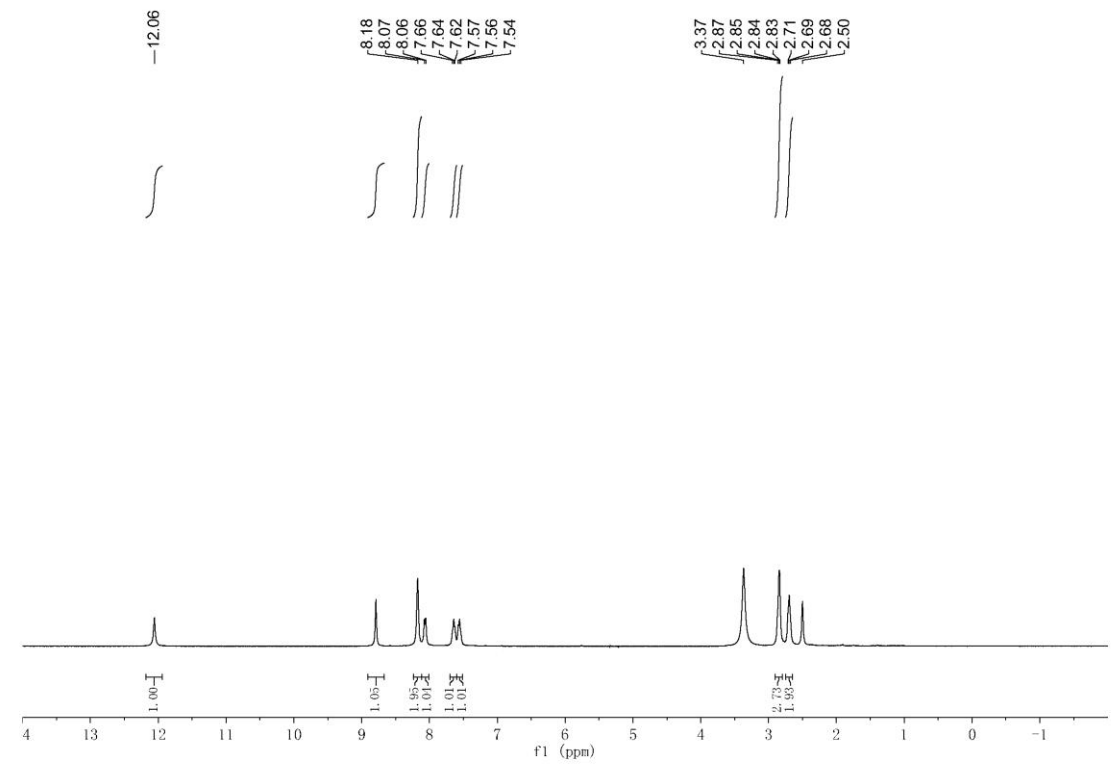

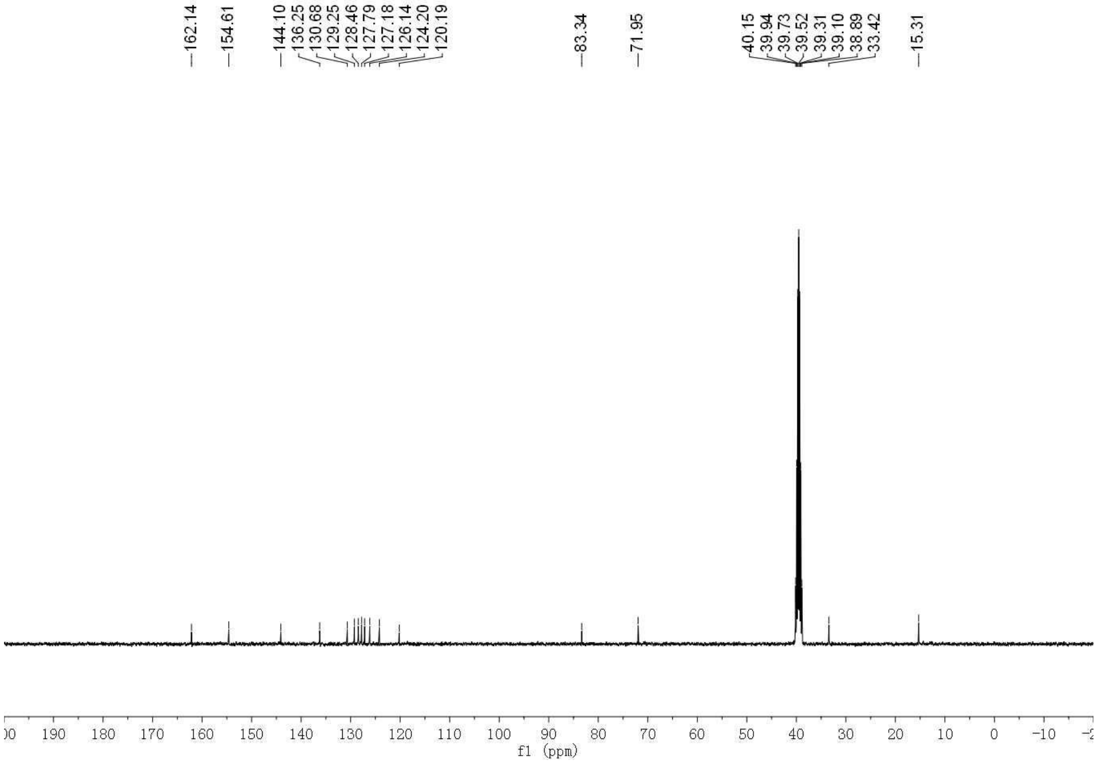

2,3-Dihydro-1-methylene-benzo[*g*]pyrrolo[2,1-*b*]quinazolin-11(1*H*)-one (9L)

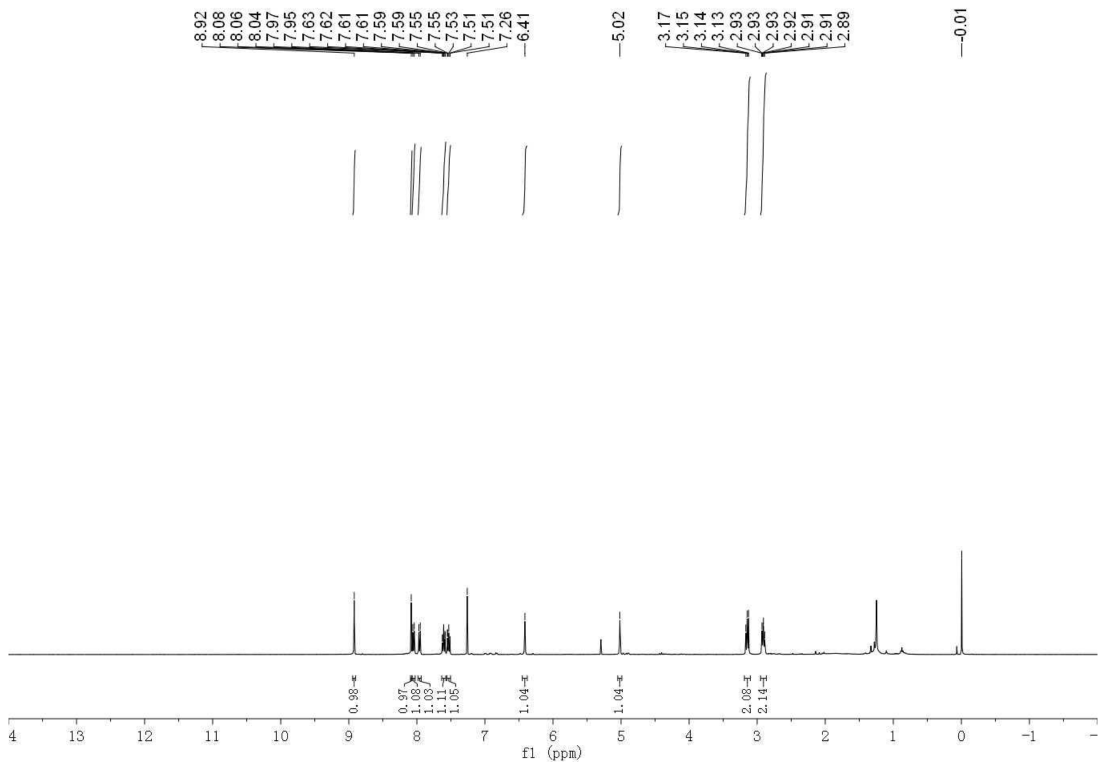

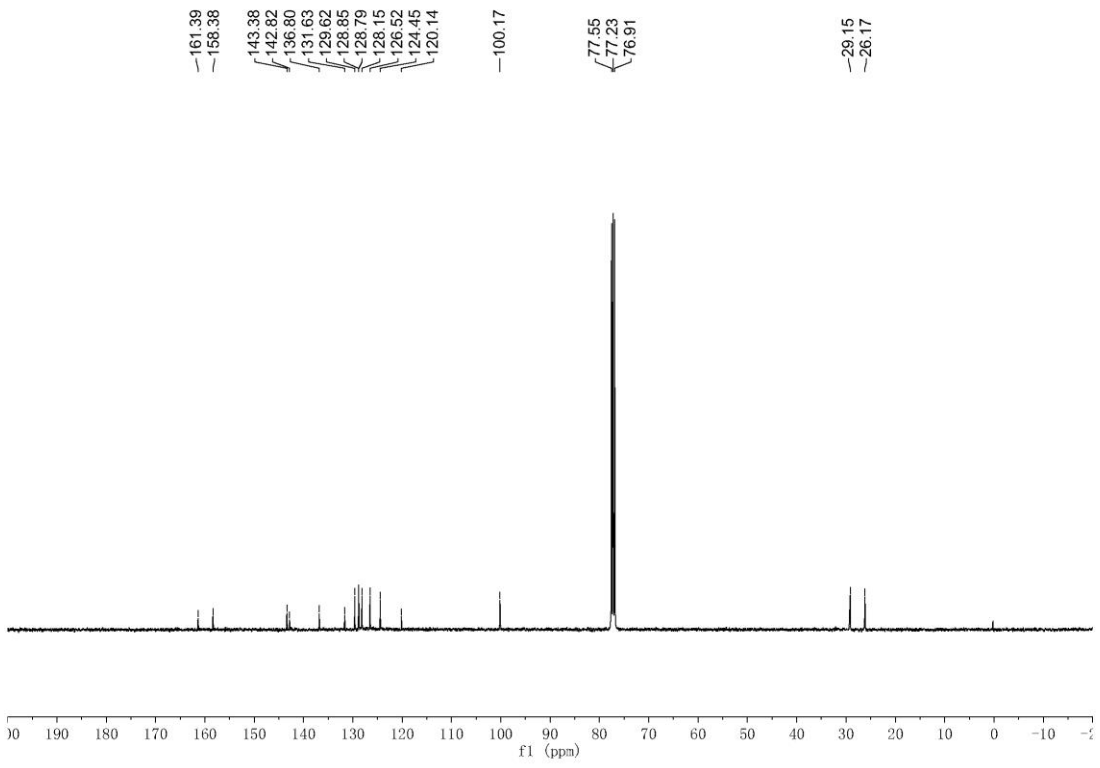

Supplement: File 1 — Detailed experimental procedures for all compounds and precursors, copies of 1H/13C NMR spectra for all compounds. [file Beilstein_J_Org_Chem-11-416-s001.pdf]
